# Supplementary material for: Bioactive Platinum(IV) Complexes Incorporating Halogenated Phenylacetates
Source: Molecules. 2022 Oct 21;27(20):7120. doi: 10.3390/molecules27207120 (PMC9611758; doi:10.3390/molecules27207120)
Supplement: Supplementary file 1 [file molecules-27-07120-s001.zip › molecules-1965556-supplementary.pdf]

SUPPLEMENTARY MATERIALS

Bioactive Platinum(IV) Complexes Incorporating Halogenated Phenylacetates

Angelico D. Aputen [1], Maria George Elias [1,2], Jayne Gilbert [3], Jennette A. Sakoff [3], Christopher P. Gordon [1], Kieran F. Scott [2] and Janice R. Aldrich-Wright [1,*]

[1] School of Science, Western Sydney University, Locked Bag 1797, Penrith South, Sydney, NSW 2751, Australia

[2] Ingham Institute, Liverpool, Sydney, NSW 2170, Australia

[3] Calvary Mater Newcastle Hospital, Waratah, Newcastle, NSW 2298, Australia

[*] Correspondence: j.aldrich-wright@westernsydney.edu.au (J.R.A.-W.);
Tel.: +61-246203218

Table of contents

HPLC chromatograms of NHS esters		
**Figure S1:**	HPLC chromatogram of NHS-4-CPA within the collection wavelength of 254 nm obtained at 298 K, by an Agilent ZORBAX RX-C_{18} column (100 × 4.6 mm, 3.5 μm pore size) using a 0 – 100% linear gradient over 15 min with a flow rate of 1 $mL.min^{-1}$.	**11**
**Figure S2:**	HPLC chromatogram of NHS-4-FPA within the collection wavelength of 254 nm obtained at 298 K, by an Agilent ZORBAX RX-C_{18} column (100 × 4.6 mm, 3.5 μm pore size) using a 0 – 100% linear gradient over 15 min with a flow rate of 1 $mL.min^{-1}$.	**11**
**Figure S3:**	HPLC chromatogram of NHS-4-BPA within the collection wavelength of 254 nm obtained at 298 K, by an Agilent ZORBAX RX-C_{18} column (100 × 4.6 mm, 3.5 μm pore size) using a 0 – 100% linear gradient over 15 min with a flow rate of 1 $mL.min^{-1}$.	**11**
**Figure S4:**	HPLC chromatogram of NHS-4-IPA within the collection wavelength of 254 nm obtained at 298 K, by an Agilent ZORBAX RX-C_{18} column (100 × 4.6 mm, 3.5 μm pore size) using a 0 – 100% linear gradient over 15 min with a flow rate of 1 $mL.min^{-1}$.	**12**
**^{1}H-NMR spectra of NHS esters**		
**Figure S5:**	^{1}H-NMR spectrum of NHS-4-CPA in CD_3CN obtained at 298 K. Inset: structure of NHS-4-CPA with proton labelling system.	**13**
**Figure S6:**	^{1}H-NMR spectrum of NHS-4-FPA in CD_3CN obtained at 298 K. Inset: expanded ^{19}F-NMR and structure of NHS-4-FPA with proton labelling system.	**14**
**Figure S7:**	^{1}H-NMR spectrum of NHS-4-BPA in CD_3CN obtained at 298 K. Inset: expanded ^{19}F-NMR and structure of NHS-4-BPA with proton labelling system.	**15**
**Figure S8:**	^{1}H-NMR spectrum of NHS-4-IPA in CD_3CN obtained at 298 K. Inset: expanded ^{19}F-NMR and structure of NHS-4-IPA with proton labelling system.	**16**

**ESI-MS spectra of NHS esters**		
**Figure S9:**	ESI-MS spectrum of NHS-4-CPA obtained at 298 K, using a Waters SYNAPT G2-Si quadruple time-of-flight (QTOF) HDMS. Inset: structure of NHS-4-CPA.	**17**
**Figure S10:**	ESI-MS spectrum of NHS-4-FPA obtained at 298 K, using a Waters SYNAPT G2-Si quadruple time-of-flight (QTOF) HDMS. Inset: structure of NHS-4-FPA.	**17**
**Figure S11:**	ESI-MS spectrum of NHS-4-BPA obtained at 298 K, using a Waters SYNAPT G2-Si quadruple time-of-flight (QTOF) HDMS. Inset: structure of NHS-4-BPA.	**18**
**Figure S12:**	ESI-MS spectrum of NHS-4-IPA obtained at 298 K, using a Waters SYNAPT G2-Si quadruple time-of-flight (QTOF) HDMS. Inset: structure of NHS-4-IPA.	**18**
**HPLC chromatograms of platinum(IV) complexes, 1–8**		
**Figure S13:**	HPLC chromatogram of **1** within the collection wavelength of 254 nm obtained at 298 K, by a Phenomenex Onyx^{TM} Monolithic C18-reverse phase column (100 × 4.6 mm, 5 μm pore size) using a 0 – 100% linear gradient over 15 min with a flow rate of 1 mL.min^{-1}.	**19**
**Figure S14:**	HPLC chromatogram of **2** within the collection wavelength of 254 nm obtained at 298 K, by a Phenomenex Onyx^{TM} Monolithic C18-reverse phase column (100 × 4.6 mm, 5 μm pore size) using a 0 – 100% linear gradient over 15 min with a flow rate of 1 mL.min^{-1}.	**19**
**Figure S15:**	HPLC chromatogram of **3** within the collection wavelength of 254 nm obtained at 298 K, by a Phenomenex Onyx^{TM} Monolithic C18-reverse phase column (100 × 4.6 mm, 5 μm pore size) using a 0 – 100% linear gradient over 15 min with a flow rate of 1 mL.min^{-1}.	**20**
**Figure S16:**	HPLC chromatogram of **4** within the collection wavelength of 254 nm obtained at 298 K, by a Phenomenex Onyx^{TM} Monolithic C18-reverse phase column (100 × 4.6 mm, 5 μm pore size) using a 0 – 100% linear gradient over 15 min with a flow rate of 1 mL.min^{-1}.	**20**
**Figure S17:**	HPLC chromatogram of **5** within the collection wavelength of 254 nm obtained at 298 K, by a Phenomenex Onyx^{TM} Monolithic C18-reverse	**21**

	phase column (100 × 4.6 mm, 5 µm pore size) using a 0 – 100% linear gradient over 15 min with a flow rate of 1 $mL.min^{-1}$.	
**Figure S18:**	HPLC chromatogram of **6** within the collection wavelength of 254 nm obtained at 298 K, by a Phenomenex Onyx™ Monolithic C18-reverse phase column (100 × 4.6 mm, 5 µm pore size) using a 0 – 100% linear gradient over 15 min with a flow rate of 1 $mL.min^{-1}$.	**21**
**Figure S19:**	HPLC chromatogram of **7** within the collection wavelength of 254 nm obtained at 298 K, by a Phenomenex Onyx™ Monolithic C18-reverse phase column (100 × 4.6 mm, 5 µm pore size) using a 0 – 100% linear gradient over 15 min with a flow rate of 1 $mL.min^{-1}$.	**22**
**Figure S20:**	HPLC chromatogram of **8** within the collection wavelength of 254 nm obtained at 298 K, by a Phenomenex Onyx™ Monolithic C18-reverse phase column (100 × 4.6 mm, 5 µm pore size) using a 0 – 100% linear gradient over 15 min with a flow rate of 1 $mL.min^{-1}$.	**22**
**^{1}H-NMR, 2D-COSY and ^{1}H-^{195}Pt-HMQC spectra of 1–8**		
**Figure S21:**	^{1}H-NMR spectrum of **1** in D_2O obtained at 298 K Inset: structure of **1** with proton labelling system.	**23**
**Figure S22:**	2D-COSY spectrum of **1** in D_2O obtained at 298 K. Inset: structure of **1** with proton labelling system.	**24**
**Figure S23:**	^{1}H-^{195}Pt-HMQC spectrum of **1** in D_2O obtained at 298 K. Region: 400 ppm. Inset: structure of **1** with proton labelling system and arrows that indicate coupling.	**25**
**Figure S24:**	^{1}H-^{195}Pt-HMQC spectrum of **1** in D_2O obtained at 298 K. Region: -2800 ppm. Absence of platinum(II) resonance.	**26**
**Figure S25:**	^{1}H-NMR spectrum of **2** in D_2O obtained at 298 K Inset: structure of **2** with proton labelling system.	**27**
**Figure S26:**	2D-COSY spectrum of **2** in D_2O obtained at 298 K. Inset: structure of **2** with proton labelling system.	**28**
**Figure S27:**	^{1}H-^{195}Pt-HMQC spectrum of **2** in D_2O obtained at 298 K. Region: 400 ppm. Inset: structure of **2** with proton labelling system and arrows that indicate coupling.	**29**
**Figure S28:**	^{1}H-^{195}Pt-HMQC spectrum of **2** in D_2O obtained at 298 K. Region: -2800 ppm. Absence of platinum(II) resonance.	**30**

**Figure S29:**	^{19}F-NMR spectrum of **3** in D_2O obtained at 298 K.	**31**
**Figure S30:**	^{1}H-NMR spectrum of **3** in D_2O obtained at 298 K Inset: structure of **3** with proton labelling system.	**32**
**Figure S31:**	2D-COSY spectrum of **3** in D_2O obtained at 298 K. Inset: structure of **3** with proton labelling system.	**33**
**Figure S32:**	^{1}H-^{195}Pt-HMQC spectrum of **3** in D_2O obtained at 298 K. Region: 400 ppm. Inset: structure of **3** with proton labelling system and arrows that indicate coupling.	**34**
**Figure S33:**	^{1}H-^{195}Pt-HMQC spectrum of **3** in D_2O obtained at 298 K. Region: -2800 ppm. Absence of platinum(II) resonance.	**35**
**Figure S34:**	^{19}F-NMR spectrum of **4** in D_2O obtained at 298 K.	**35**
**Figure S35:**	^{1}H-NMR spectrum of **4** in D_2O obtained at 298 K Inset: structure of **4** with proton labelling system.	**36**
**Figure S36:**	2D-COSY spectrum of **4** in D_2O obtained at 298 K. Inset: structure of **4** with proton labelling system.	**37**
**Figure S37:**	^{1}H-^{195}Pt-HMQC spectrum of **4** in D_2O obtained at 298 K. Region: 400 ppm. Inset: structure of **4** with proton labelling system and arrows that indicate coupling.	**38**
**Figure S38:**	^{1}H-^{195}Pt-HMQC spectrum of **4** in D_2O obtained at 298 K. Region: -2800 ppm. Absence of platinum(II) resonance.	**39**
**Figure S39:**	^{1}H-NMR spectrum of **5** in D_2O obtained at 298 K Inset: structure of **5** with proton labelling system.	**40**
**Figure S40:**	2D-COSY spectrum of **5** in D_2O obtained at 298 K. Inset: structure of **5** with proton labelling system.	**41**
**Figure S41:**	^{1}H-^{195}Pt-HMQC spectrum of **5** in D_2O obtained at 298 K. Region: 400 ppm. Inset: structure of **5** with proton labelling system and arrows that indicate coupling.	**42**
**Figure S42:**	^{1}H-^{195}Pt-HMQC spectrum of **5** in D_2O obtained at 298 K. Region: -2800 ppm. Absence of platinum(II) resonance.	**43**
**Figure S43:**	^{1}H-NMR spectrum of **6** in D_2O obtained at 298 K Inset: structure of **6** with proton labelling system.	**44**
**Figure S44:**	2D-COSY spectrum of **6** in D_2O obtained at 298 K. Inset: structure of **6** with proton labelling system.	**45**

**Figure S45:**	^{1}H-^{195}Pt-HMQC spectrum of **6** in D_2O obtained at 298 K. Region: 400 ppm. Inset: structure of **6** with proton labelling system and arrows that indicate coupling.	**46**
**Figure S46:**	^{1}H-^{195}Pt-HMQC spectrum of **6** in D_2O obtained at 298 K. Region: -2800 ppm. Absence of platinum(II) resonance.	**47**
**Figure S47:**	^{1}H-NMR spectrum of **7** in D_2O obtained at 298 K Inset: structure of **7** with proton labelling system.	**48**
**Figure S48:**	2D-COSY spectrum of **7** in D_2O obtained at 298 K. Inset: structure of **7** with proton labelling system.	**49**
**Figure S49:**	^{1}H-^{195}Pt-HMQC spectrum of **7** in D_2O obtained at 298 K. Region: 400 ppm. Inset: structure of **7** with proton labelling system and arrows that indicate coupling.	**50**
**Figure S50:**	^{1}H-^{195}Pt-HMQC spectrum of **7** in D_2O obtained at 298 K. Region: -2800 ppm. Absence of platinum(II) resonance.	**51**
**Figure S51:**	^{1}H-NMR spectrum of **8** in D_2O obtained at 298 K Inset: structure of **8** with proton labelling system.	**52**
**Figure S52:**	2D-COSY spectrum of **8** in D_2O obtained at 298 K. Inset: structure of **8** with proton labelling system.	**53**
**Figure S53:**	^{1}H-^{195}Pt-HMQC spectrum of **8** in D_2O obtained at 298 K. Region: 400 ppm. Inset: structure of **8** with proton labelling system and arrows that indicate coupling.	**54**
**Figure S54:**	^{1}H-^{195}Pt-HMQC spectrum of **8** in D_2O obtained at 298 K. Region: -2800 ppm. Absence of platinum(II) resonance.	**55**
**UV spectra of 1–8**		
**Figure S55:**	UV spectra of **1** obtained at 298 K. Inset: structure of **1** and generated plot curves within the wavelengths 279 and 304 nm.	**56**
**Figure S56:**	UV spectra of **2** obtained at 298 K. Inset: structure of **2** and generated plot curves within the wavelengths 239, 287 and 317 nm.	**57**
**Figure S57:**	UV spectra of **3** at 298 K. Inset: structure of **3** and generated plot curves within the wavelengths 204 and 279 nm.	**57**
**Figure S58:**	UV spectra of **4** obtained at 298 K. Inset: structure of **4** and generated plot curves within the wavelengths 248, 293 and 317 nm.	**58**

**Figure S59:**	UV spectra of **5** obtained at 298 K. Inset: structure of **5** and generated plot curves within the wavelengths 279 and 307 nm.	**58**
**Figure S60:**	UV spectra of **6** obtained at 298 K. Inset: structure of **6** and generated plot curves within the wavelengths 248, 291 and 317 nm.	**59**
**Figure S61:**	UV spectra of **7** obtained at 298 K. Inset: structure of **7** and generated plot curves within the wavelengths 280 and 306 nm.	**59**
**Figure S62:**	UV spectra of **8** obtained at 298 K. Inset: structure of **8** and generated plot curves within the wavelengths 230, 290 and 317 nm.	**60**
**CD spectra of 1–8**		
**Figure S63:**	CD spectrum of **1** obtained at 298 K. Inset: structure of **1**.	**61**
**Figure S64:**	CD spectrum of **2** obtained at 298 K. Inset: structure of **2**.	**61**
**Figure S65:**	CD spectrum of **3** obtained at 298 K. Inset: structure of **3**.	**62**
**Figure S66:**	CD spectrum of **4** obtained at 298 K. Inset: structure of **4**.	**62**
**Figure S67:**	CD spectrum of **5** obtained at 298 K. Inset: structure of **5**.	**63**
**Figure S68:**	CD spectrum of **6** obtained at 298 K. Inset: structure of **6**.	**63**
**Figure S69:**	CD spectrum of **7** obtained at 298 K. Inset: structure of **7**.	**64**
**Figure S70:**	CD spectrum of **8** obtained at 298 K. Inset: structure of **8**.	**64**
**ESI-MS spectra of 1–8**		
**Figure S71:**	ESI-MS spectrum of **1** obtained at 298 K, using a Waters SYNAPT G2-Si quadruple time-of-flight (QTOF) HDMS. Inset: structure of **1**.	**65**
**Figure S72:**	ESI-MS spectrum of **2** obtained at 298 K, using a Waters SYNAPT G2-Si quadruple time-of-flight (QTOF) HDMS. Inset: structure of **2**.	**66**
**Figure S73:**	ESI-MS spectrum of **3** obtained at 298 K, using a Waters SYNAPT G2-Si quadruple time-of-flight (QTOF) HDMS. Inset: structure of **3**.	**67**
**Figure S74:**	ESI-MS spectrum of **4** obtained at 298 K, using a Waters SYNAPT G2-Si quadruple time-of-flight (QTOF) HDMS. Inset: structure of **4**.	**68**
**Figure S75:**	ESI-MS spectrum of **5** obtained at 298 K, using a Waters SYNAPT G2-Si quadruple time-of-flight (QTOF) HDMS. Inset: structure of **5**.	**69**
**Figure S76:**	ESI-MS spectrum of **6** obtained at 298 K, using a Waters SYNAPT G2-Si quadruple time-of-flight (QTOF) HDMS. Inset: structure of **6**.	**70**

**Figure S77:**	ESI-MS spectrum of **7** obtained at 298 K, using a Waters SYNAPT G2-Si quadruple time-of-flight (QTOF) HDMS. Inset: structure of **7**.	**71**
**Figure S78:**	ESI-MS spectrum of **8** obtained at 298 K, using a Waters SYNAPT G2-Si quadruple time-of-flight (QTOF) HDMS. Inset: structure of **8**.	**72**
**Stability studies**		
**Figure S79:**	HPLC chromatograms of **1–8** in 10 mM PBS (~7.4 pH) pre- and post-incubation (36 h) obtained at 298 K. Inset: structures of **1–8**.	**73**
**Lipophilicity measurements**		
**Figure S80:**	Generated plot curves of log k versus concentration of organic solvent, CH_3CN to determine the chromatographic lipophilicity index, log kw of **1–8**.	**74**
**Reduction reaction experiments**		
**Figure S81:**	Preliminary 1D-^{195}Pt-NMR spectra of **1**, **2**, **3** and **4** in 10 mM PBS (~7.4 pH) within the regions of -2800 and 400 ppm at 37 °C.	**75**
**Figure S82:**	Preliminary 1D-^{195}Pt-NMR spectra of **5**, **6**, **7** and **8** in 10 mM PBS (~7.4 pH) within the regions of -2800 and 400 ppm at 37 °C.	**76**
**Figure S83:**	^{1}H-NMR spectra of **3** with 10 mM PBS (~7.4 pH) and AsA in D_2O at 310.15 K, in different time intervals, highlighting the movement of resonances from the phenanthroline protons and the aromatic protons of the 4-FPA ligand as indicated by the red arrows. **T** represents time in min.	**77**
**Figure S84:**	1D-^{195}Pt-NMR spectra of **3** with 10 mM PBS (~7.4 pH) and AsA in D_2O, within the regions of -2800 and 400 ppm at 310.15 K, highlighting its complete reduction after 1 h from the final ^{1}H-NMR experiment.	**77**
**Figure S85:**	^{1}H-NMR spectra of **4** with 10 mM PBS (~7.4 pH) and AsA in D_2O at 310.15 K, in different time intervals, highlighting the movement of resonances from the phenanthroline protons and the aromatic protons of the 4-FPA ligand as indicated by the red arrows. **T** represents time in min.	**78**
**Figure S86:**	1D-^{195}Pt-NMR spectra of **4** with 10 mM PBS (~7.4 pH) and AsA in D_2O, within the regions of -2800 and 400 ppm at 310.15 K, highlighting	**78**

	its partial reduction after 1 h from the final ^{1}H-NMR experiment as indicated by *.	
**Figure S87:**	^{1}H-NMR spectra of **5** with 10 mM PBS (~7.4 pH) and AsA in D_2O at 310.15 K, in different time intervals, highlighting the movement of resonances from the phenanthroline protons and the aromatic protons of the 4-FPA ligand as indicated by the red arrows. **T** represents time in min.	**79**
**Figure S88:**	1D-^{195}Pt-NMR spectra of **5** with 10 mM PBS (~7.4 pH) and AsA in D_2O, within the regions of -2800 and 400 ppm at 310.15 K, highlighting its partial reduction after 1 h from the final ^{1}H-NMR experiment as indicated by *.	**79**
**Figure S89:**	^{1}H-NMR spectra of **6** with 10 mM PBS (~7.4 pH) and AsA in D_2O at 310.15 K, in different time intervals, highlighting the movement of resonances from the phenanthroline protons and the aromatic protons of the 4-FPA ligand as indicated by the red arrows. **T** represents time in min.	**80**
**Figure S90:**	1D-^{195}Pt-NMR spectra of **6** with 10 mM PBS (~7.4 pH) and AsA in D_2O, within the regions of -2800 and 400 ppm at 310.15 K, highlighting its partial reduction after 1 h from the final ^{1}H-NMR experiment as indicated by *.	**80**
**Figure S91:**	^{1}H-NMR spectra of **7** with 10 mM PBS (~7.4 pH) and AsA in D_2O at 310.15 K, in different time intervals, highlighting the movement of resonances from the phenanthroline protons and the aromatic protons of the 4-FPA ligand as indicated by the red arrows. **T** represents time in min.	**81**
**Figure S92:**	1D-^{195}Pt-NMR spectra of **7** with 10 mM PBS (~7.4 pH) and AsA in D_2O, within the regions of -2800 and 400 ppm at 310.15 K, highlighting its partial reduction after 1 h from the final ^{1}H-NMR experiment as indicated by *.	**81**
**Figure S93:**	^{1}H-NMR spectra of **8** with 10 mM PBS (~7.4 pH) and AsA in D_2O at 310.15 K, in different time intervals, highlighting the movement of resonances from the phenanthroline protons and the aromatic protons	**82**

	of the 4-FPA ligand as indicated by the red arrows. **T** represents time in min.	
**Figure S94:**	1D-^{195}Pt-NMR spectra of **8** with 10 mM PBS (~7.4 pH) and AsA in D_2O, within the regions of -2800 and 400 ppm at 310.15 K, highlighting its complete reduction after 1 h from the final ^{1}H-NMR experiment.	**82**
**ROS experiments**		
**Figure S95:**	ROS production upon treatment with **1–8**, 4-CPA, 4-FPA, 4-BPA, 4-IPA and cisplatin in HT29 colon cells at 0, 0.25, 0.5, 1, 3, 6, 12, 24, 48 and 72 h. **PHEN*SS*(IV)-4CPA** (**1**), **56ME*SS*(IV)-4CPA** (**2**), **PHEN*SS*(IV)-4FPA** (**3**), **56ME*SS*(IV)-4FPA** (**4**), **PHEN*SS*(IV)-4BPA** (**5**), **56ME*SS*(IV)-4BPA** (**6**), **PHEN*SS*(IV)-4IPA** (**7**), **56ME*SS*(IV)-4IPA** (**8**). TBHP: t-butyl hydroperoxide, NAC: *N*-acetylcysteine. Data points denote mean ± SEM. *n* = 3 from three independent experiments where samples were run in triplicates.	**83**

<u>**HPLC chromatograms of NHS esters**</u>

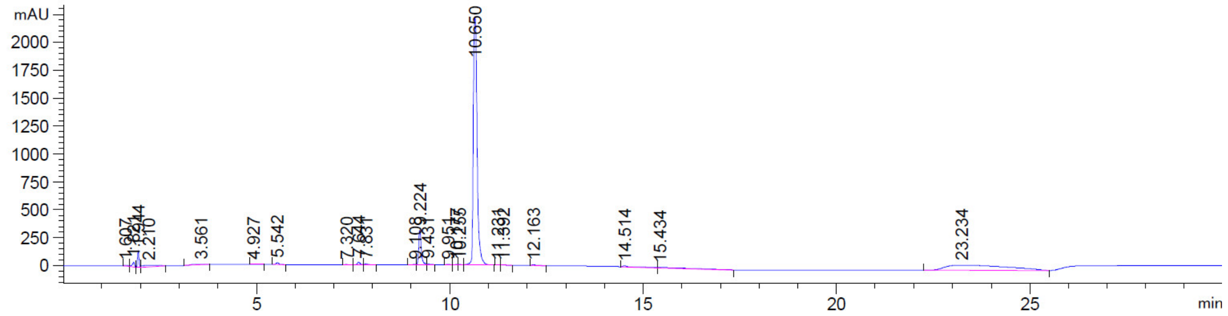

Figure S1: HPLC chromatogram of NHS-4-CPA within the collection wavelength of 254 nm obtained at 298 K, by an Agilent ZORBAX RX-C_{18} column (100 × 4.6 mm, 3.5 μm pore size) using a 0 – 100% linear gradient over 15 min with a flow rate of 1 mL.min^{-1}.

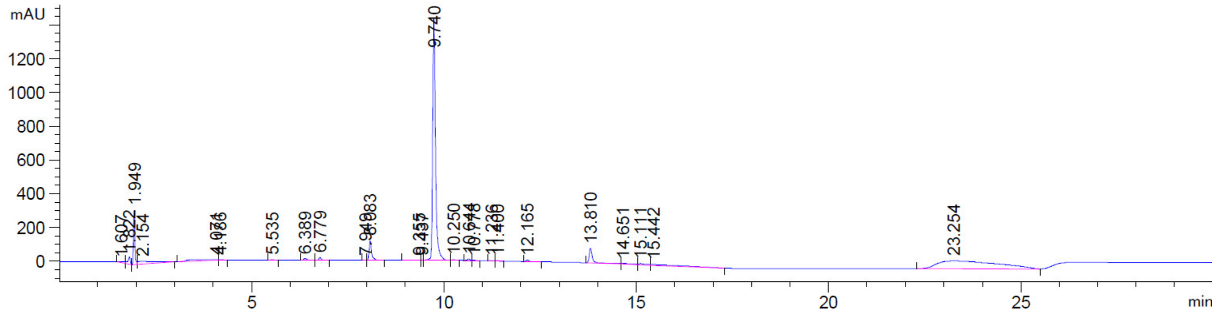

Figure S2: HPLC chromatogram of NHS-4-FPA within the collection wavelength of 254 nm obtained at 298 K, by an Agilent ZORBAX RX-C_{18} column (100 × 4.6 mm, 3.5 μm pore size) using a 0 – 100% linear gradient over 15 min with a flow rate of 1 mL.min^{-1}.

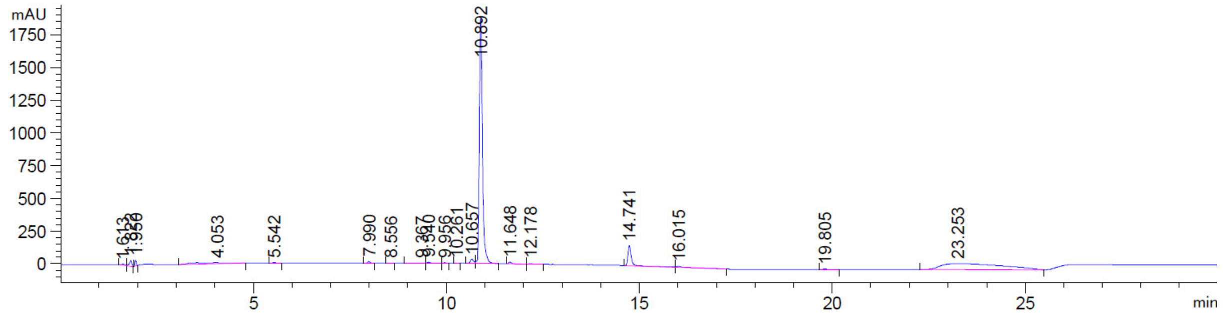

Figure S3: HPLC chromatogram of NHS-4-BPA within the collection wavelength of 254 nm obtained at 298 K, by an Agilent ZORBAX RX-C_{18} column (100 × 4.6 mm, 3.5 μm pore size) using a 0 – 100% linear gradient over 15 min with a flow rate of 1 mL.min^{-1}.

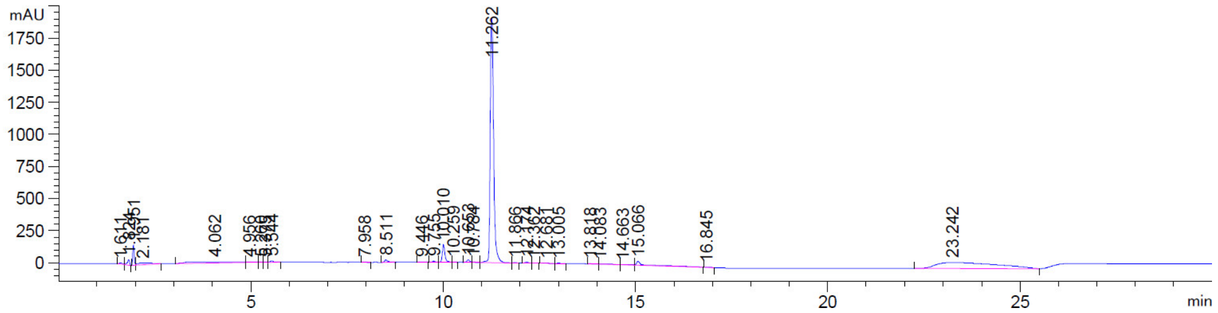

Figure S4: HPLC chromatogram of NHS-4-IPA within the collection wavelength of 254 nm obtained at 298 K, by an Agilent ZORBAX RX-C_{18} column (100 × 4.6 mm, 3.5 µm pore size) using a 0 – 100% linear gradient over 15 min with a flow rate of 1 mL.min^{-1}.

^{1}H-NMR spectra of NHS esters

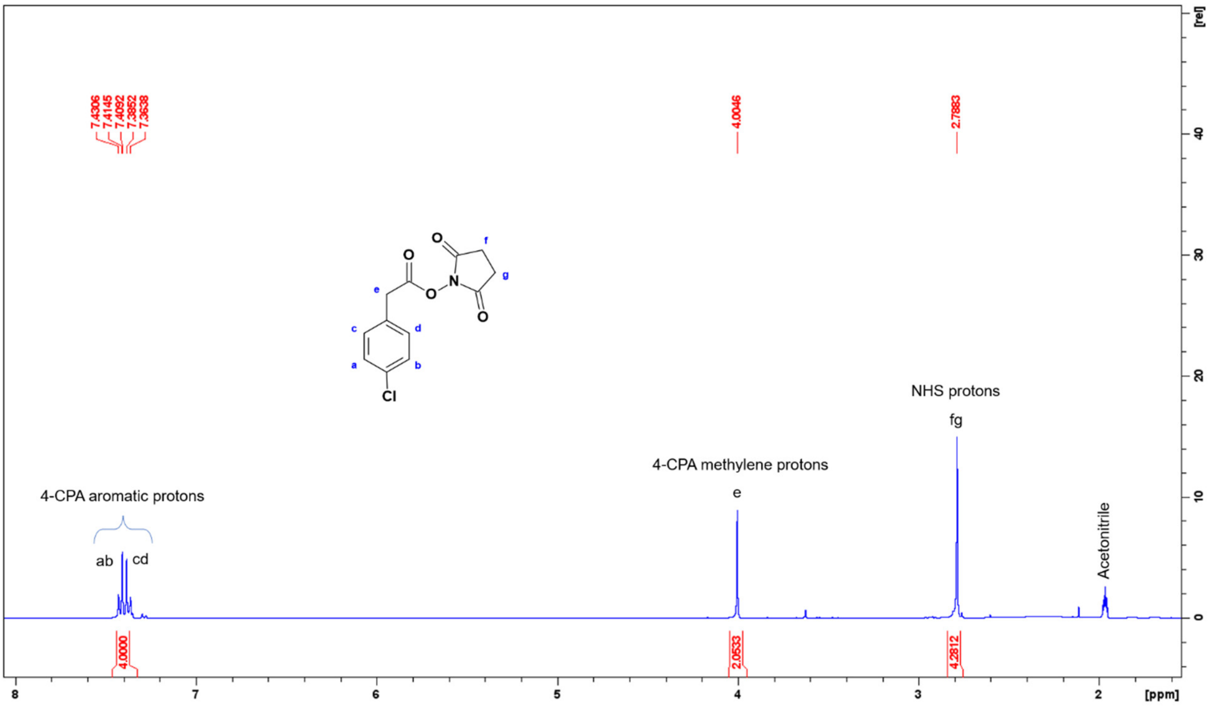

Figure S5: ^{1}H-NMR spectrum of NHS-4-CPA in CD_3CN obtained at 298 K. Inset: structure of NHS-4-CPA with proton labelling system.

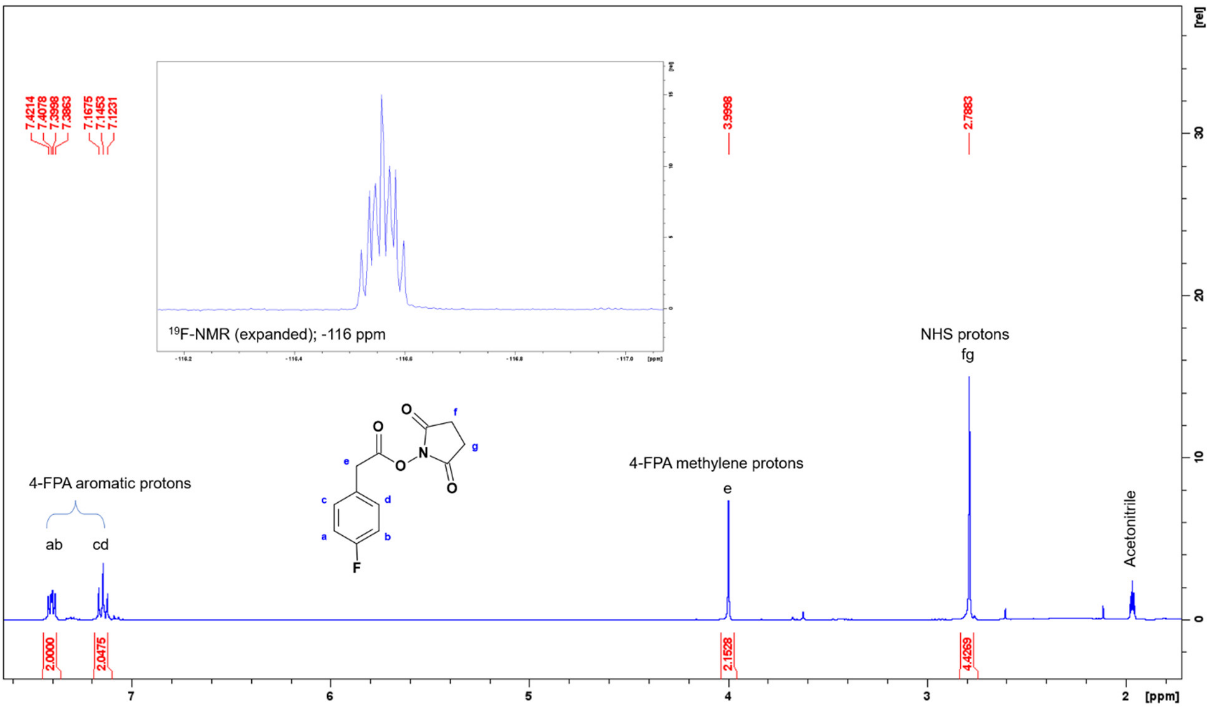

Figure S6: ^{1}H-NMR spectrum of NHS-4-FPA in CD_3CN obtained at 298 K. Inset: expanded ^{19}F-NMR and structure of NHS-4-FPA with proton labelling system.

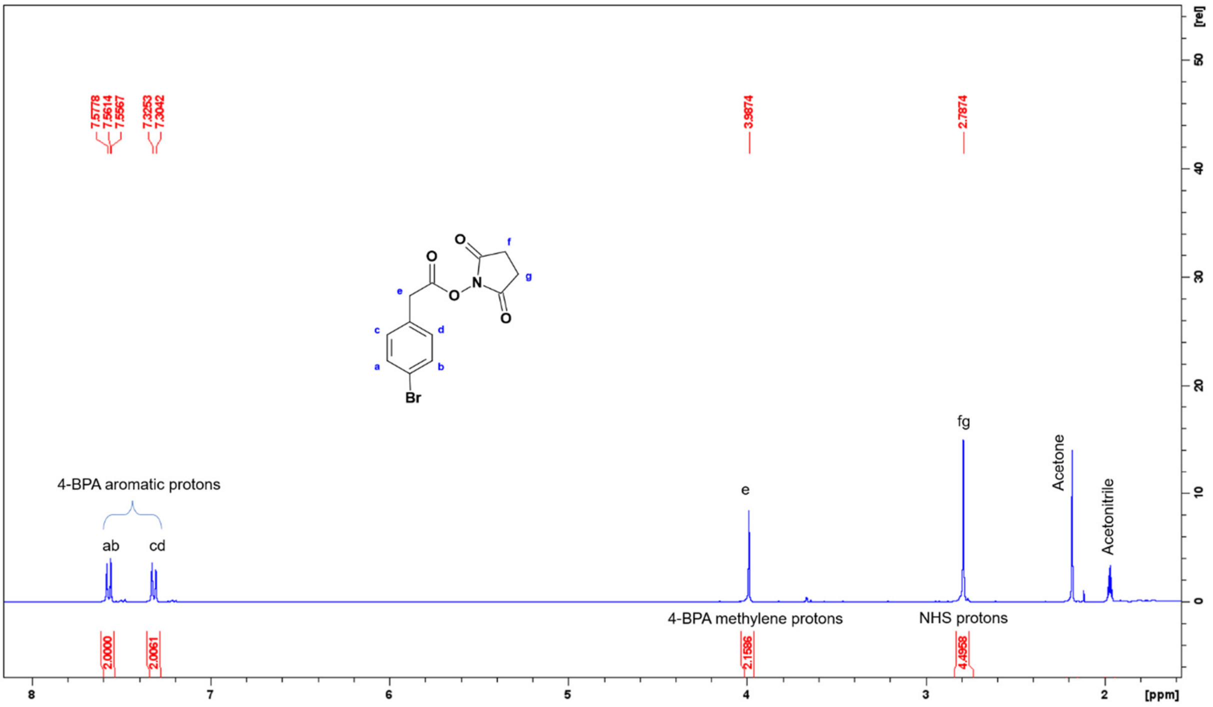

Figure S7: ^{1}H-NMR spectrum of NHS-4-BPA in CD_3CN obtained at 298 K. Inset: structure of NHS-4-BPA with proton labelling system.

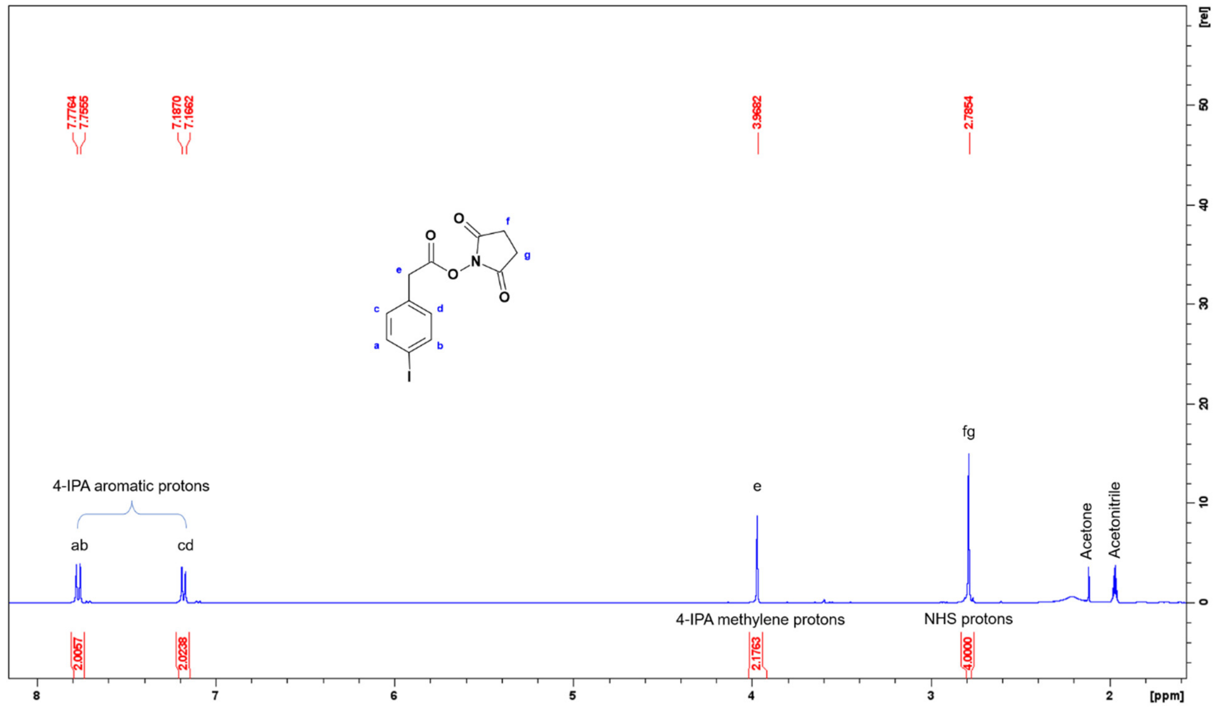

Figure S8: ^{1}H-NMR spectrum of NHS-4-IPA in CD_3CN obtained at 298 K. Inset: structure of NHS-4-IPA with proton labelling system.

ESI-MS spectra of NHS esters

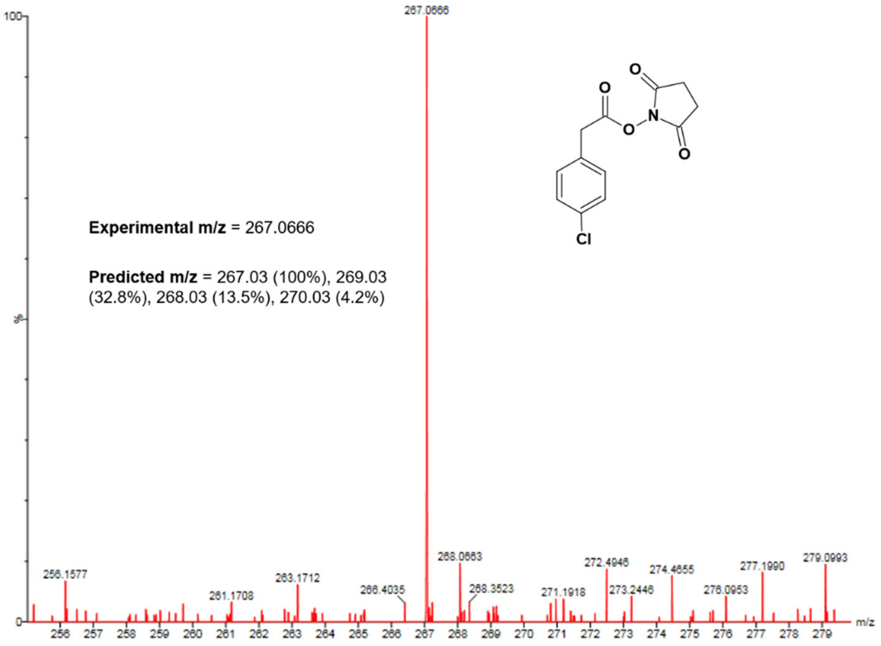

Figure S9: ESI-MS spectrum of NHS-4-CPA obtained at 298 K, using a Waters SYNAPT G2-Si quadruple time-of-flight (QTOF) HDMS. Inset: structure of NHS-4-CPA.

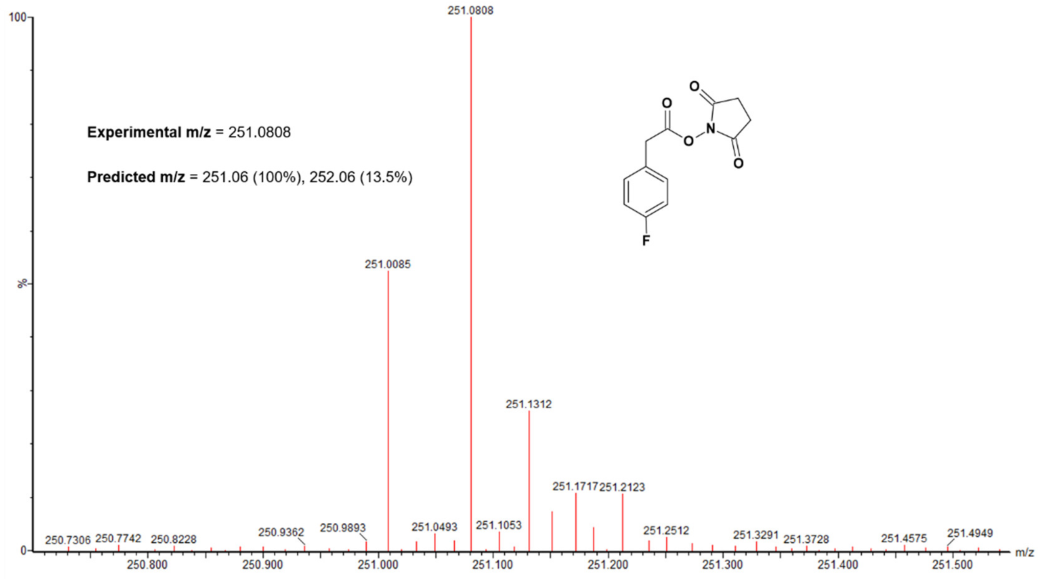

Figure S10: ESI-MS spectrum of NHS-4-FPA obtained at 298 K, using a Waters SYNAPT G2-Si quadruple time-of-flight (QTOF) HDMS. Inset: structure of NHS-4-FPA.

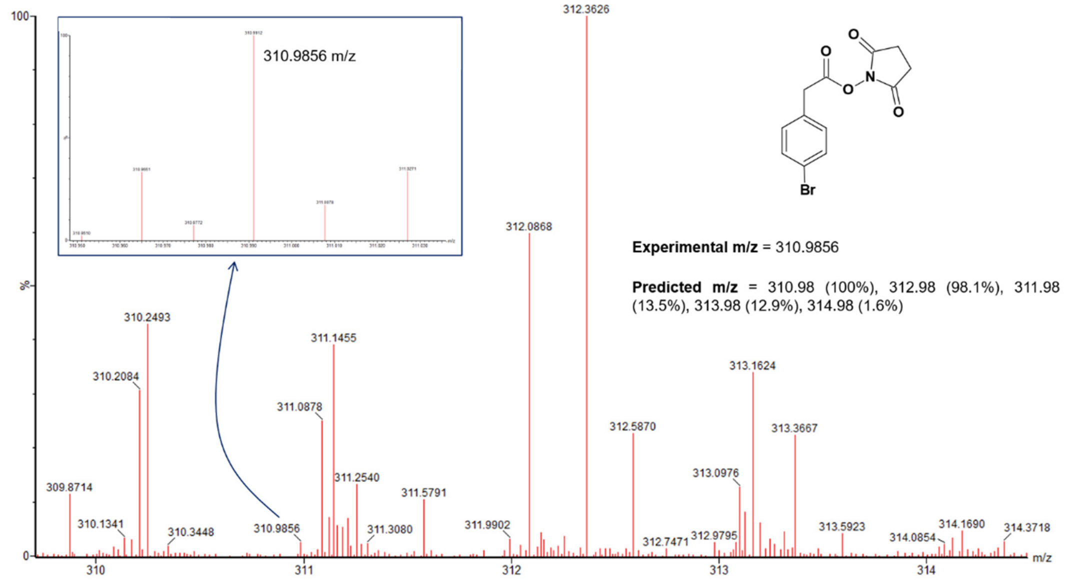

Figure S11: ESI-MS spectrum of NHS-4-BPA obtained at 298 K, using a Waters SYNAPT G2-Si quadruple time-of-flight (QTOF) HDMS. Inset: structure of NHS-4-BPA.

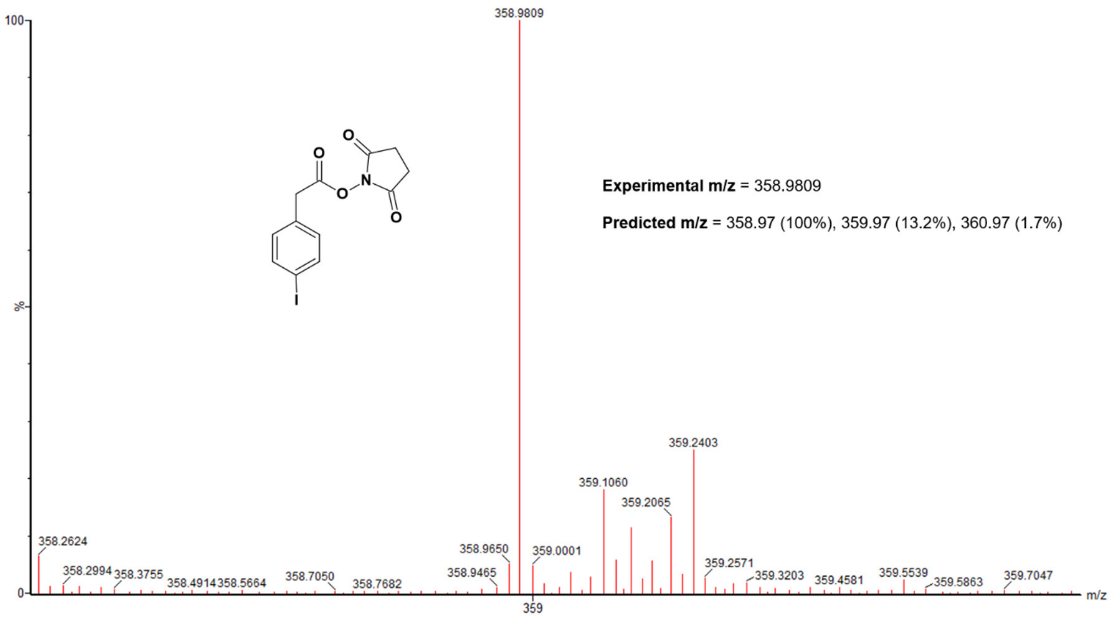

Figure S12: ESI-MS spectrum of NHS-4-IPA obtained at 298 K, using a Waters SYNAPT G2-Si quadruple time-of-flight (QTOF) HDMS. Inset: structure of NHS-4-IPA.

HPLC chromatograms of platinum(IV) complexes, 1 – 8

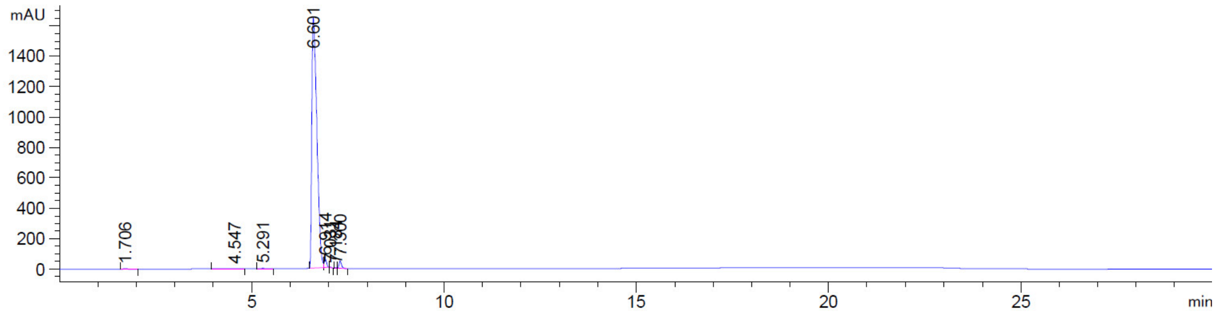

Figure S13: HPLC chromatogram of **1** within the collection wavelength of 254 nm obtained at 298 K, by a Phenomenex Onyx™ Monolithic C_{18}-reverse phase column (100 × 4.6 mm, 5 µm pore size) using a 0 – 100% linear gradient over 15 min with a flow rate of 1 mL.min^{-1}.

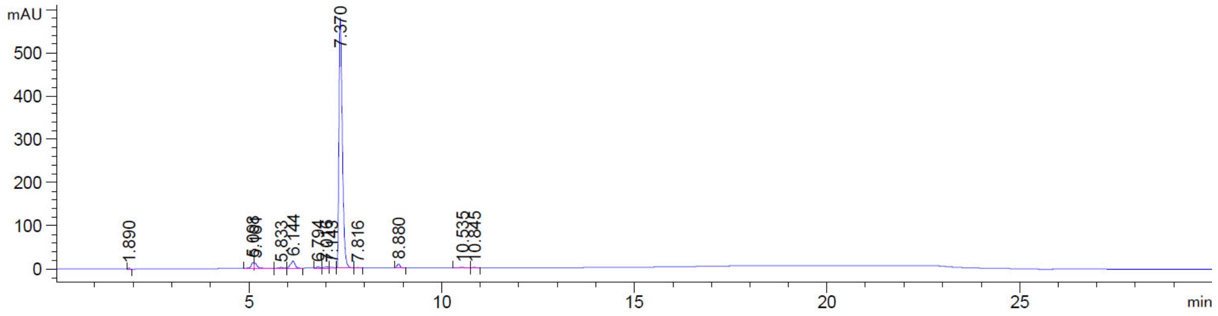

Figure S14: HPLC chromatogram of **2** within the collection wavelength of 254 nm obtained at 298 K, by a Phenomenex Onyx™ Monolithic C_{18}-reverse phase column (100 × 4.6 mm, 5 µm pore size) using a 0 – 100% linear gradient over 15 min with a flow rate of 1 mL.min^{-1}.

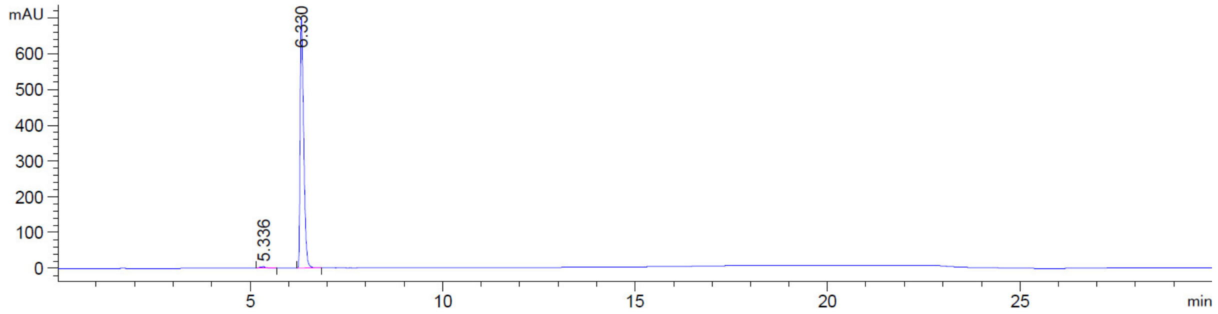

Figure S15: HPLC chromatogram of **3** within the collection wavelength of 254 nm obtained at 298 K, by a Phenomenex Onyx™ Monolithic C_{18}-reverse phase column (100 × 4.6 mm, 5 µm pore size) using a 0 – 100% linear gradient over 15 min with a flow rate of 1 $mL.min^{-1}$.

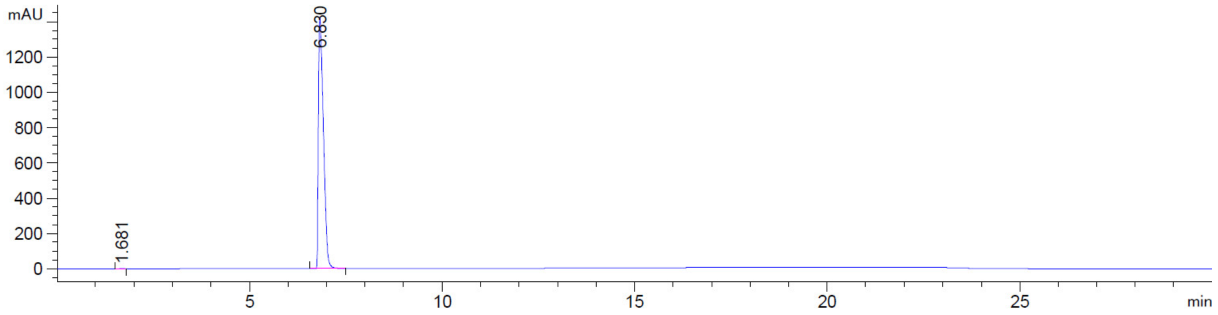

Figure S16: HPLC chromatogram of **4** within the collection wavelength of 254 nm obtained at 298 K, by a Phenomenx Onyx™ Monolithic C_{18}-reverse phase column (100 × 4.6 mm, 5 µm pore size) using a 0 – 100% linear gradient over 15 min with a flow rate of 1 $mL.min^{-1}$.

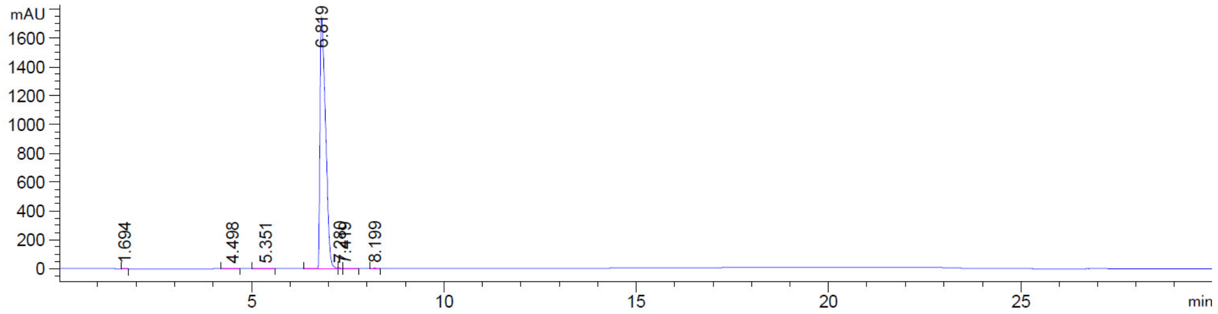

Figure S17: HPLC chromatogram of **5** within the collection wavelength of 254 nm obtained at 298 K, by a Phenomenx Onyx™ Monolithic C_{18}-reverse phase column (100 × 4.6 mm, 5 μm pore size) using a 0 – 100% linear gradient over 15 min with a flow rate of 1 mL.min^{-1}.

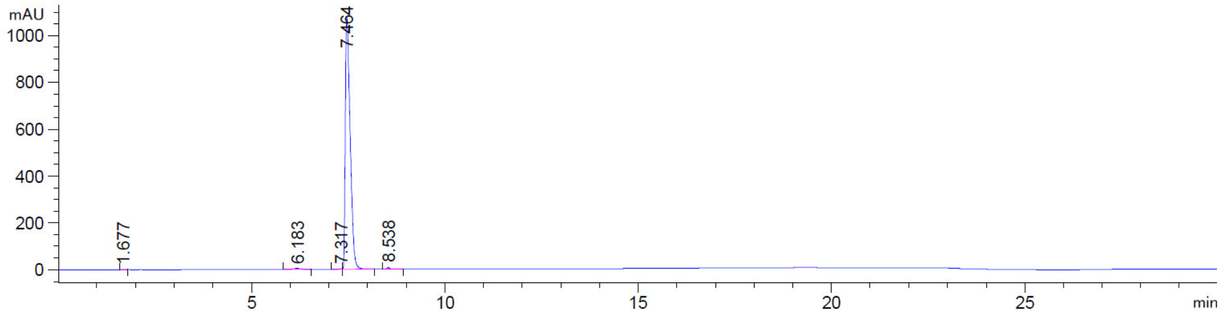

Figure S18: HPLC chromatogram of **6** within the collection wavelength of 254 nm obtained at 298 K, by a Phenomenx Onyx™ Monolithic C_{18}-reverse phase column (100 × 4.6 mm, 5 μm pore size) using a 0 – 100% linear gradient over 15 min with a flow rate of 1 mL.min^{-1}.

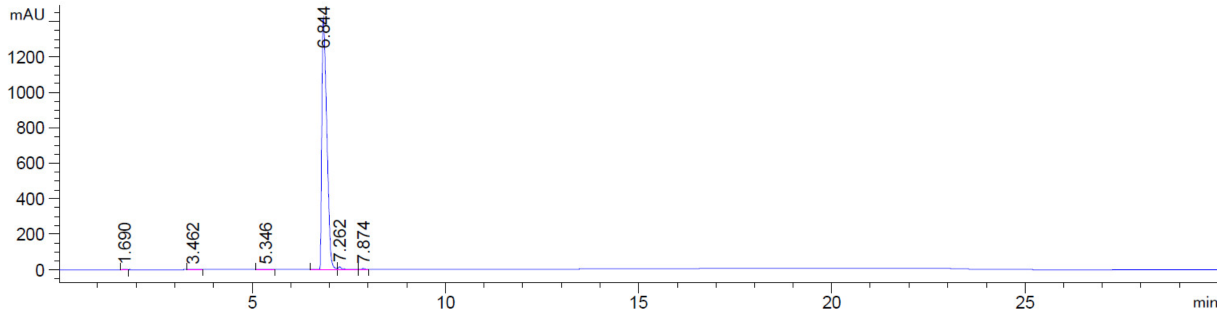

Figure S19: HPLC chromatogram of **7** within the collection wavelength of 254 nm obtained at 298 K, by a Phenomenx Onyx™ Monolithic C_{18}-reverse phase column (100 × 4.6 mm, 5 μm pore size) using a 0 – 100% linear gradient over 15 min with a flow rate of 1 $mL.min^{-1}$.

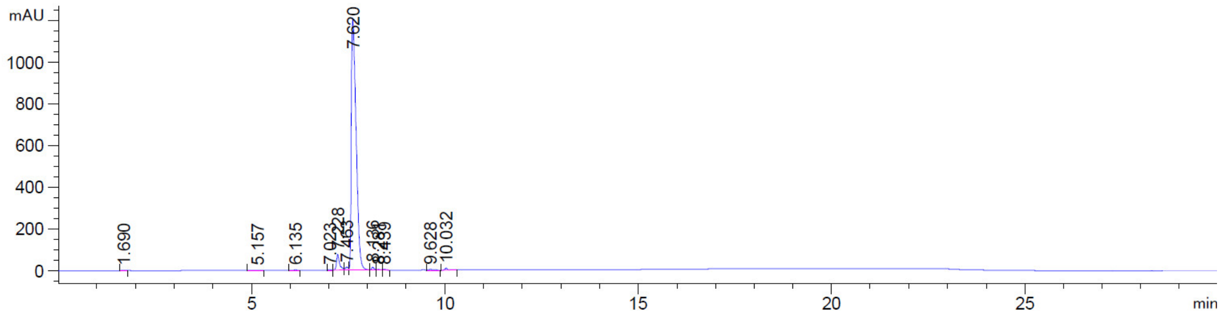

Figure S20: HPLC chromatogram of **8** within the collection wavelength of 254 nm obtained at 298 K, by a Phenomenx Onyx™ Monolithic C_{18}-reverse phase column (100 × 4.6 mm, 5 μm pore size) using a 0 – 100% linear gradient over 15 min with a flow rate of 1 $mL.min^{-1}$.

^{1}H-NMR, 2D-COSY and ^{1}H-^{195}Pt-HMQC spectra of 1 – 8

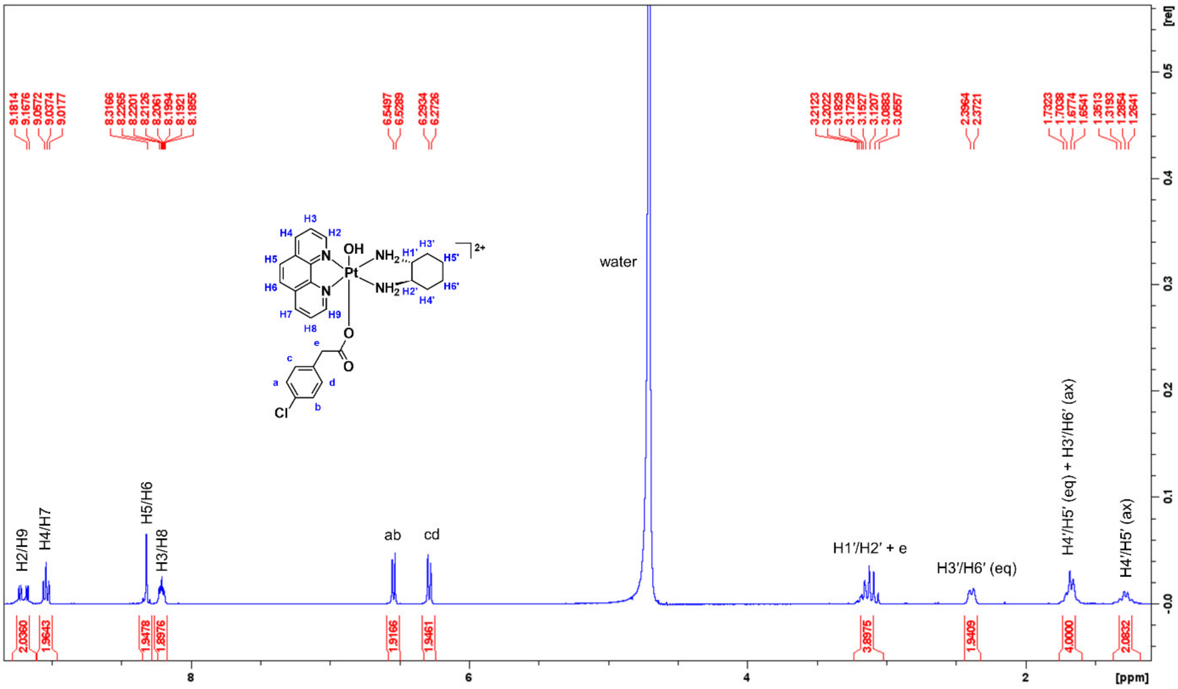

Figure S21: ^{1}H-NMR spectrum of **1** in D_2O obtained at 298 K Inset: structure of **1** with proton labelling system.

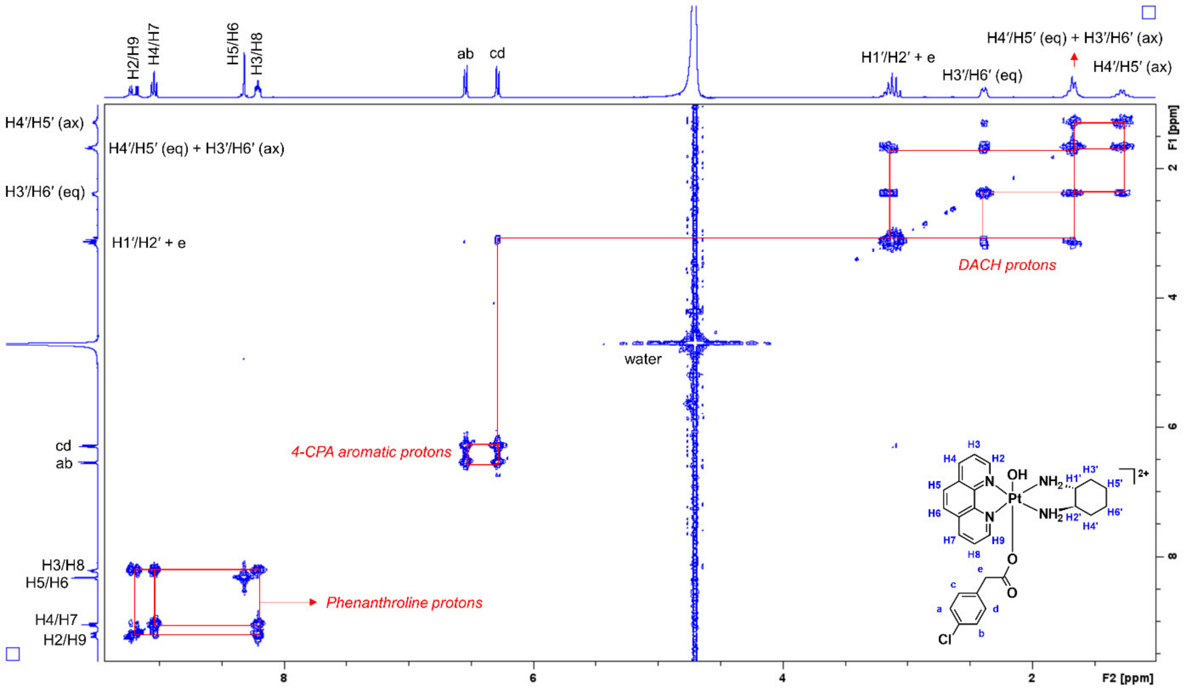

Figure S22: 2D-COSY spectrum of **1** in D2O obtained at 298 K. Inset: structure of **1** with proton labelling system.

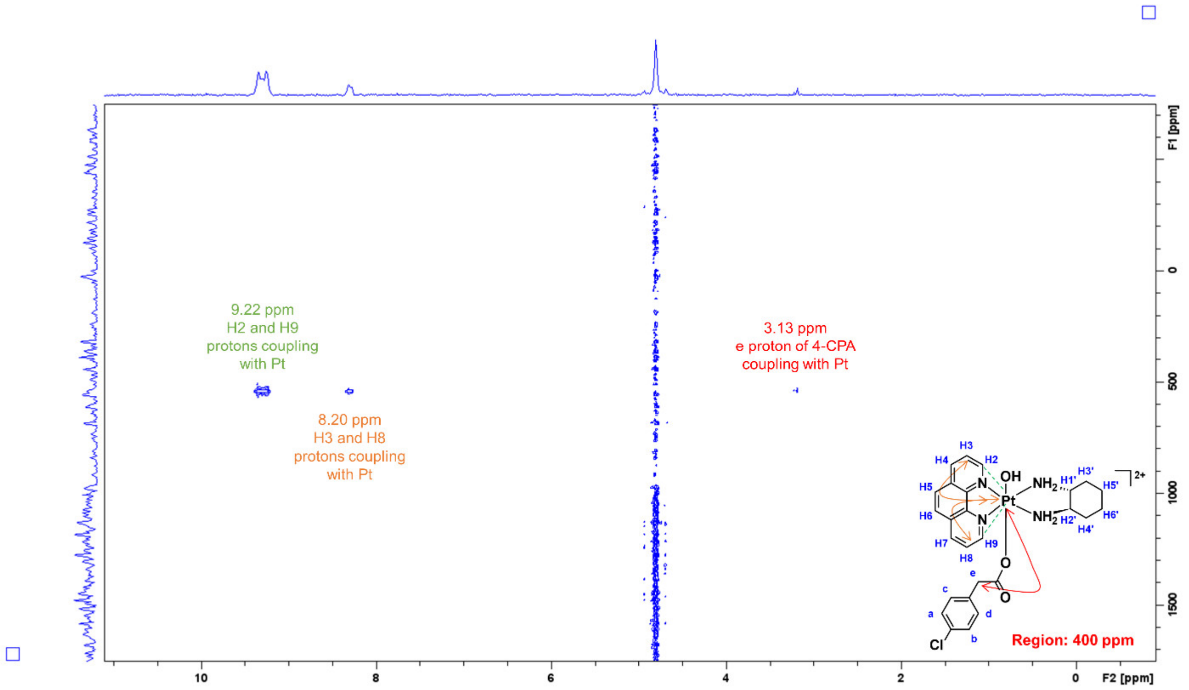

Figure S23: ^{1}H-^{195}Pt-HMQC spectrum of **1** in D_2O obtained at 298 K. Region: 400 ppm. Inset: structure of **1** with proton labelling system and arrows that indicate coupling.

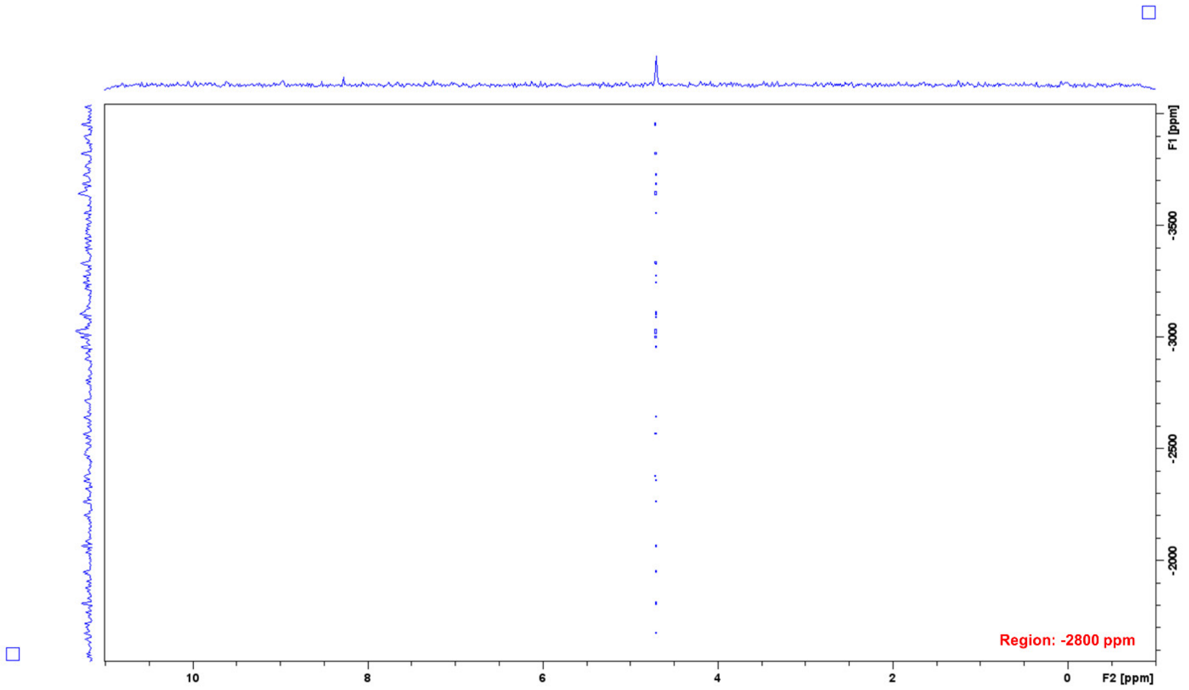

Figure S24: ^{1}H-^{195}Pt-HMQC spectrum of **1** in D_2O obtained at 298 K. Region: -2800 ppm. Absence of platinum(II) resonance.

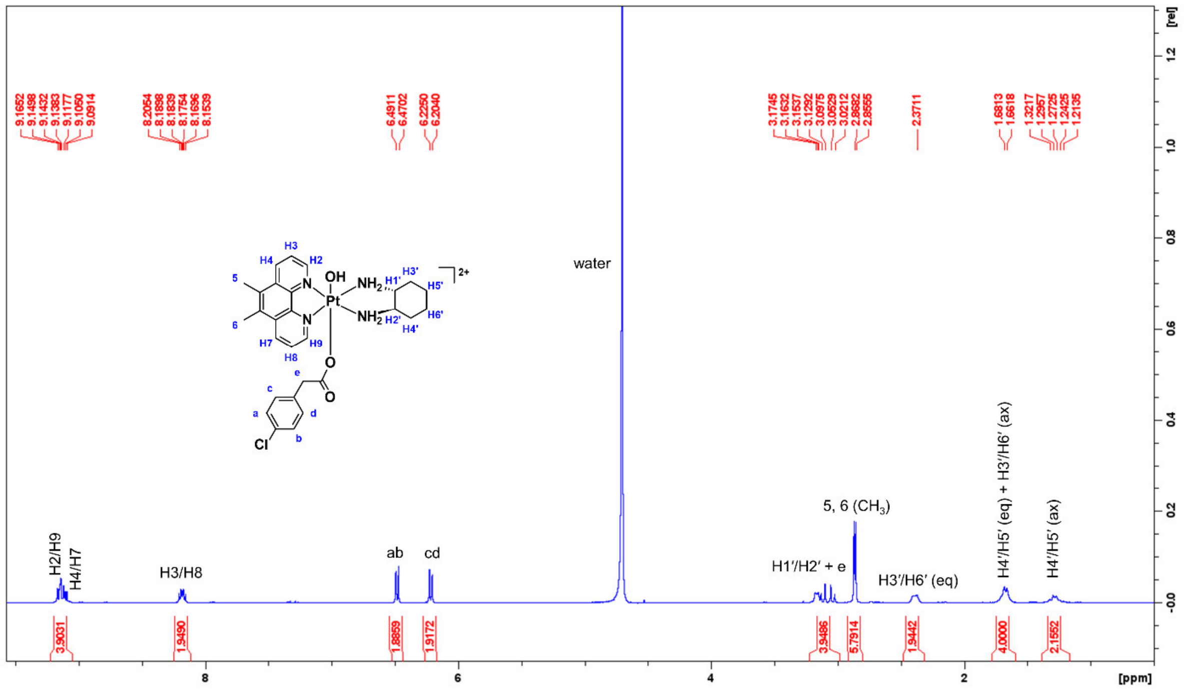

Figure S25: ^{1}H-NMR spectrum of **2** in D_2O obtained at 298 K Inset: structure of **2** with proton labelling system.

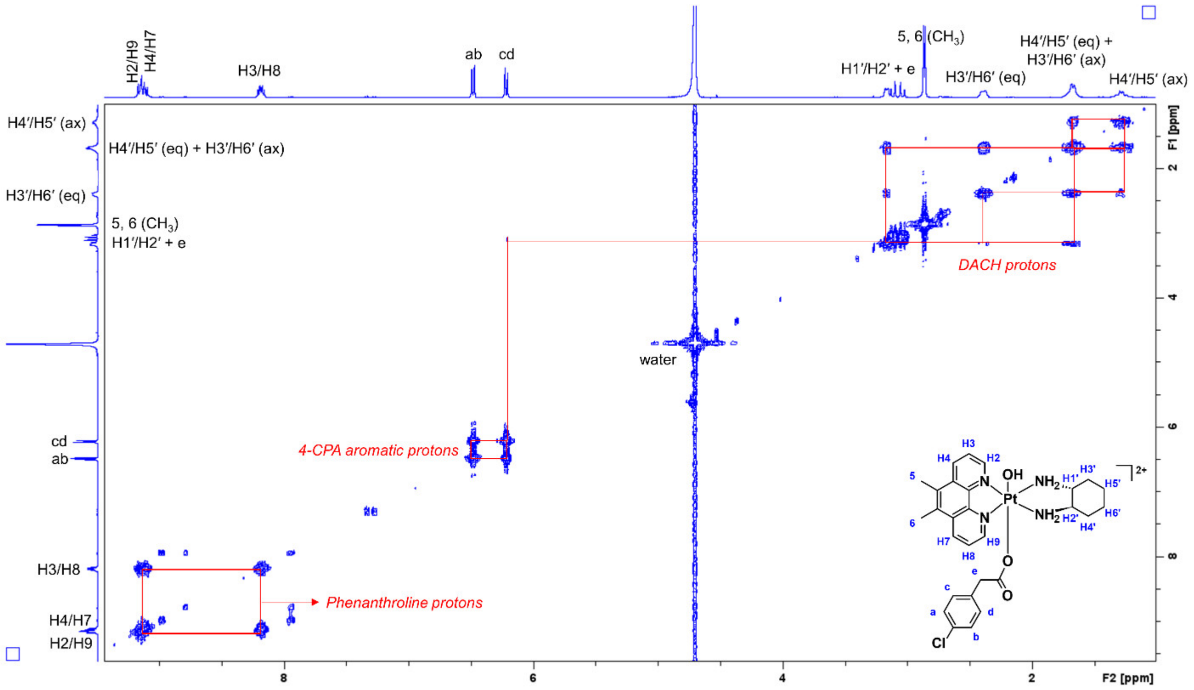

Figure S26: 2D-COSY spectrum of **2** in D_2O obtained at 298 K. Inset: structure of **2** with proton labelling system.

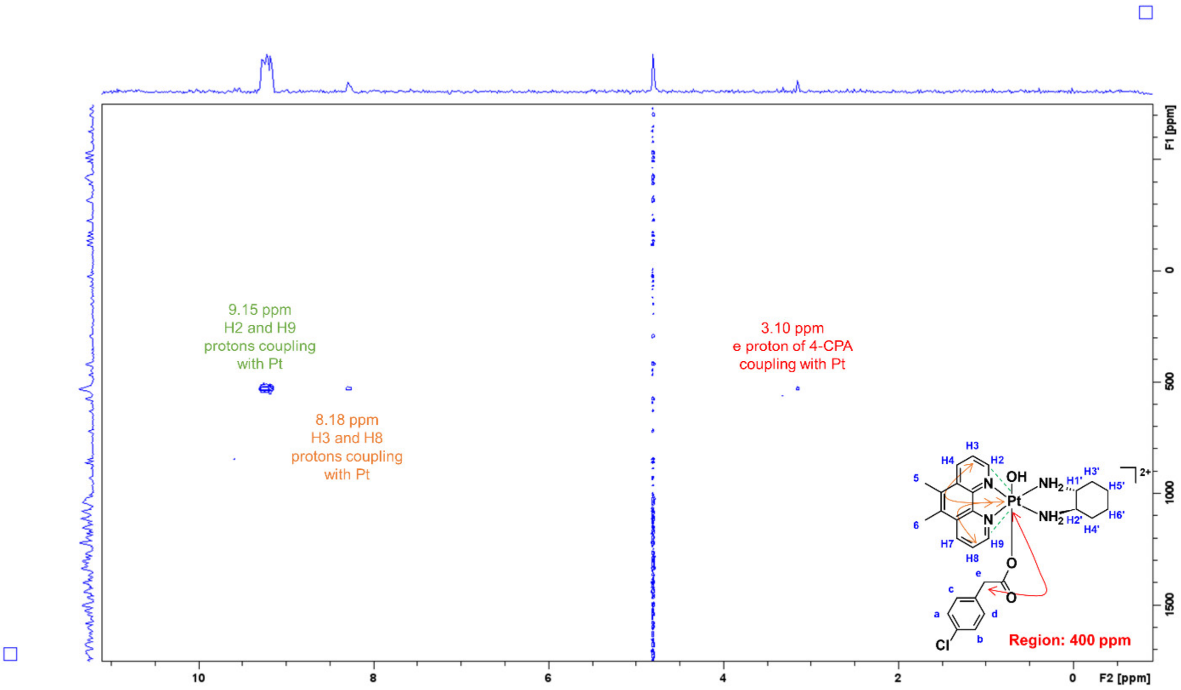

Figure S27: ^{1}H-^{195}Pt-HMQC spectrum of **2** in D_2O obtained at 298 K. Region: 400 ppm. Inset: structure of **2** with proton labelling system and arrows that indicate coupling.

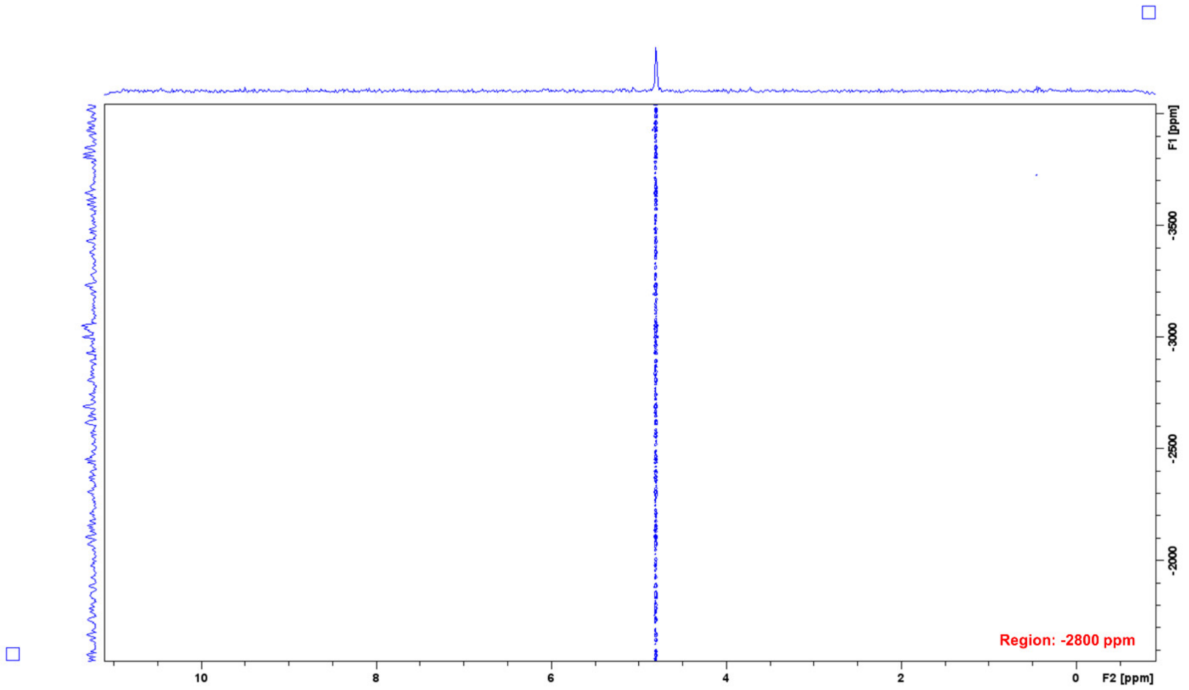

Figure S28: ^{1}H-^{195}Pt-HMQC spectrum of **2** in D_2O obtained at 298 K. Region: -2800 ppm. Absence of platinum(II) resonance.

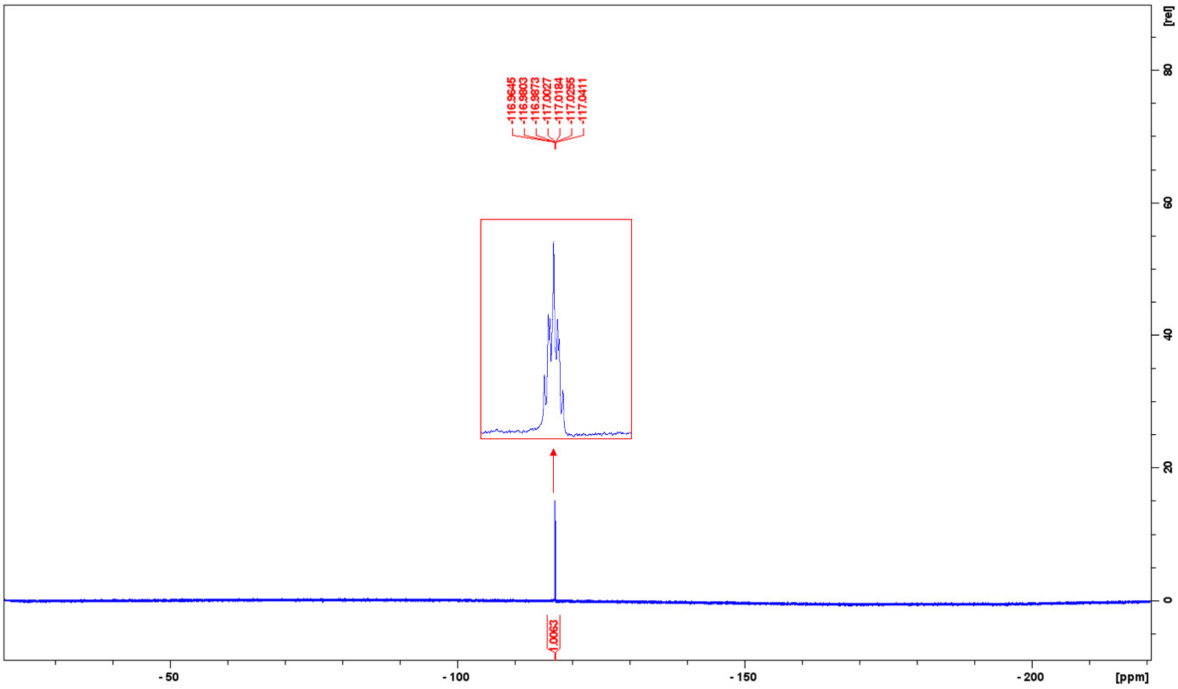

Figure S29: ^{19}F-NMR spectrum of **3** in D_2O obtained at 298 K.

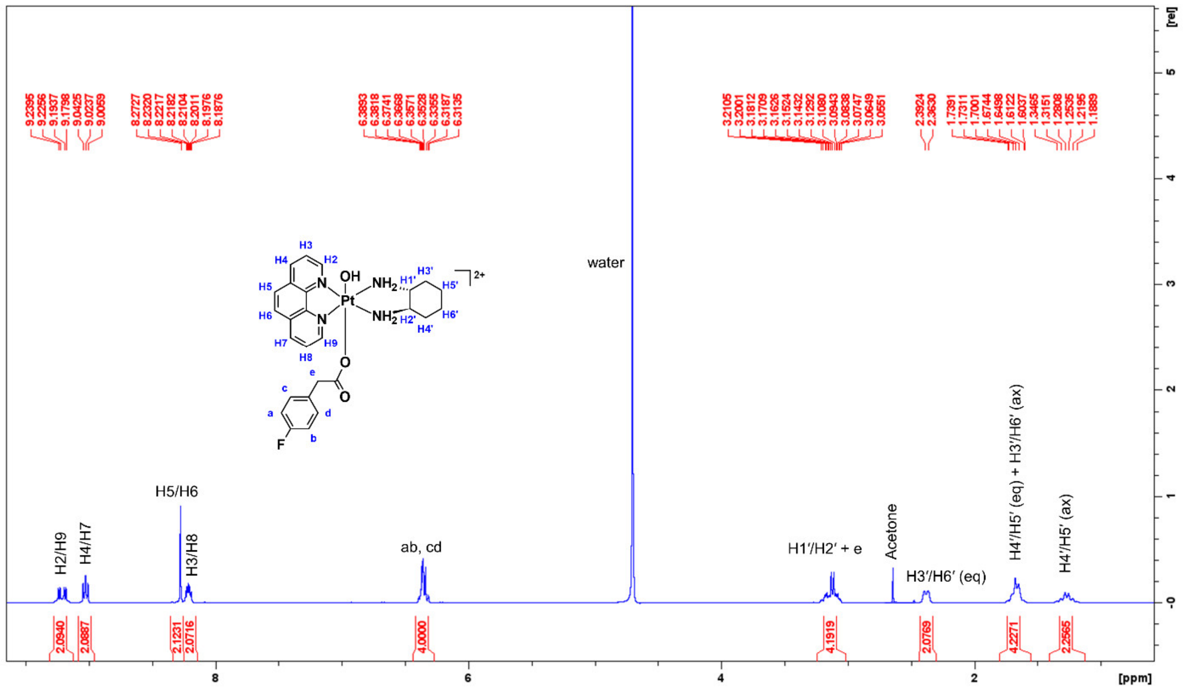

Figure S30: ^{1}H-NMR spectrum of **3** in D_2O obtained at 298 K Inset: structure of **3** with proton labelling system.

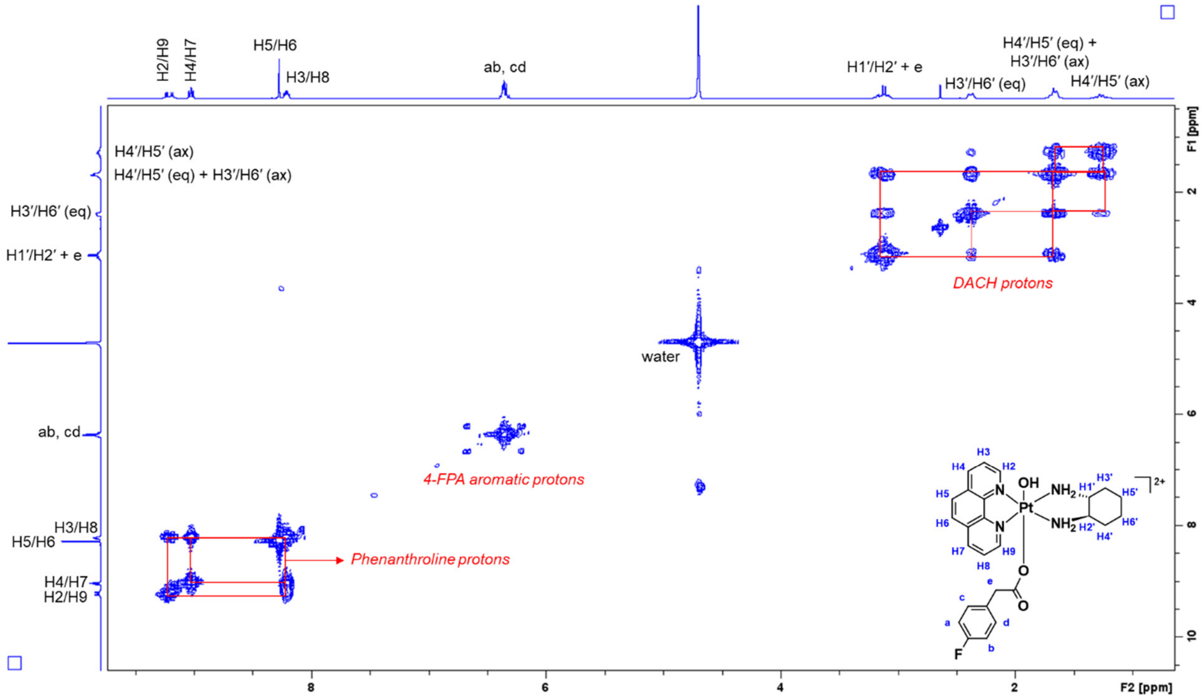

Figure S31: 2D-COSY spectrum of **3** in D_2O obtained at 298 K. Inset: structure of **3** with proton labelling system.

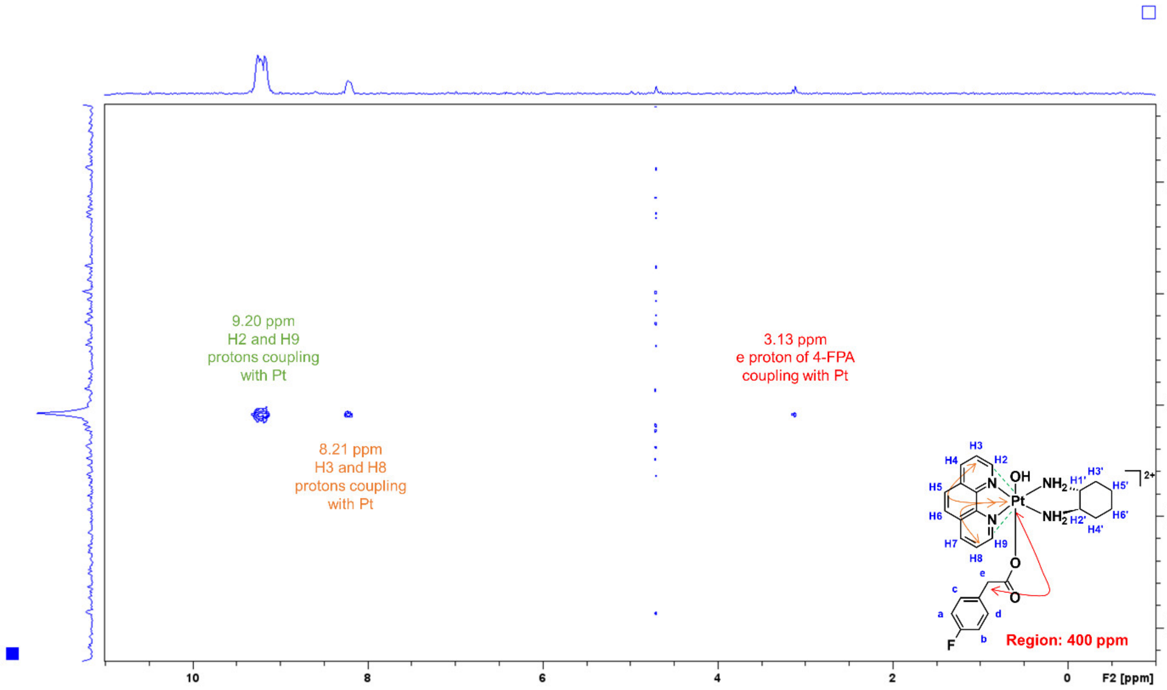

Figure S32: ^{1}H-^{195}Pt-HMQC spectrum of **3** in D_2O obtained at 298 K. Region: 400 ppm. Inset: structure of **3** with proton labelling system and arrows that indicate coupling.

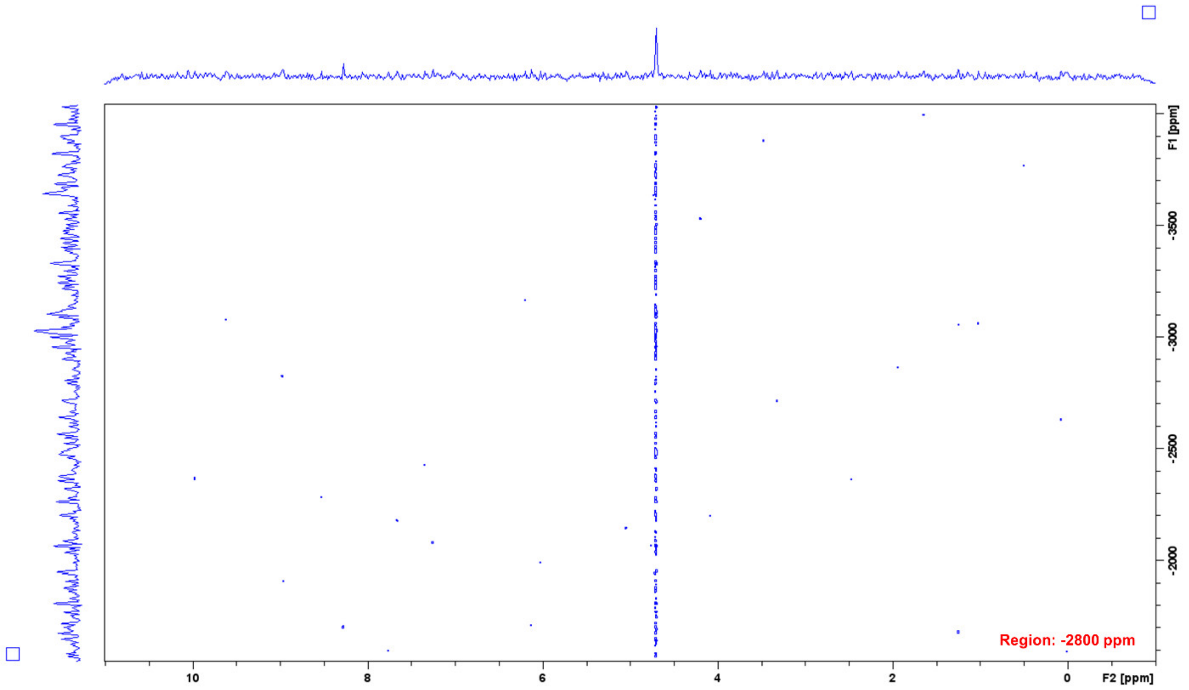

Figure S33: ^{1}H-^{195}Pt-HMQC spectrum of **3** in D_2O obtained at 298 K. Region: -2800 ppm. Absence of platinum(II) resonance.

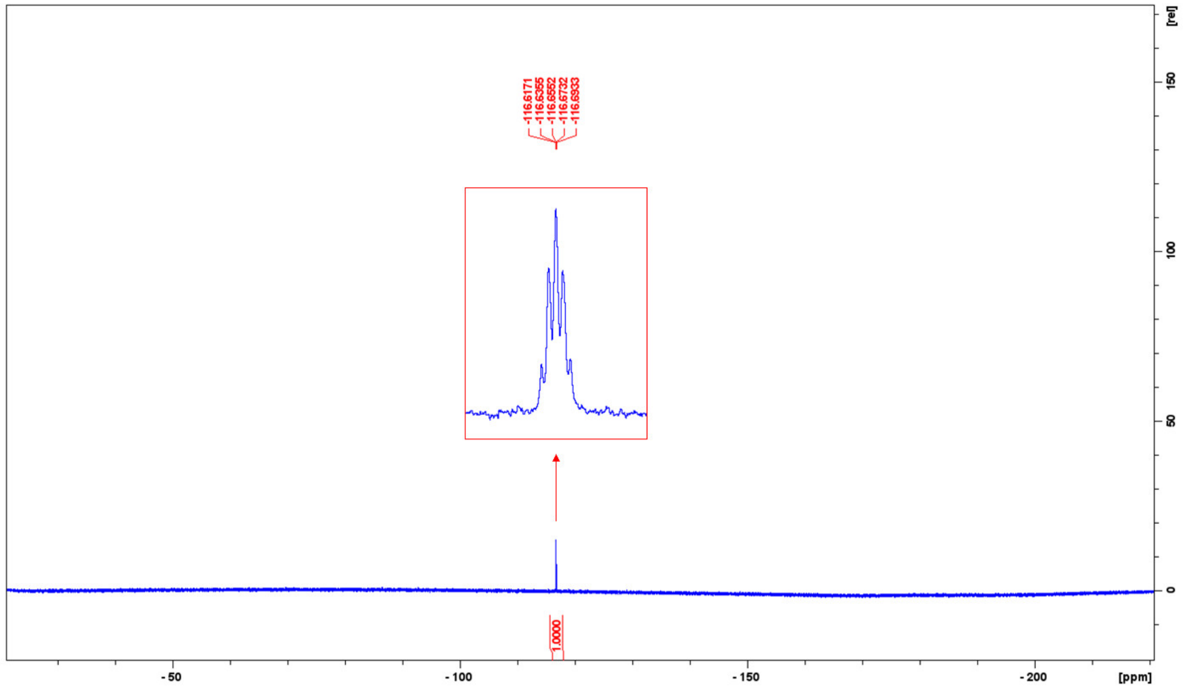

Figure S34: ^{19}F-NMR spectrum of **4** in D_2O obtained at 298 K.

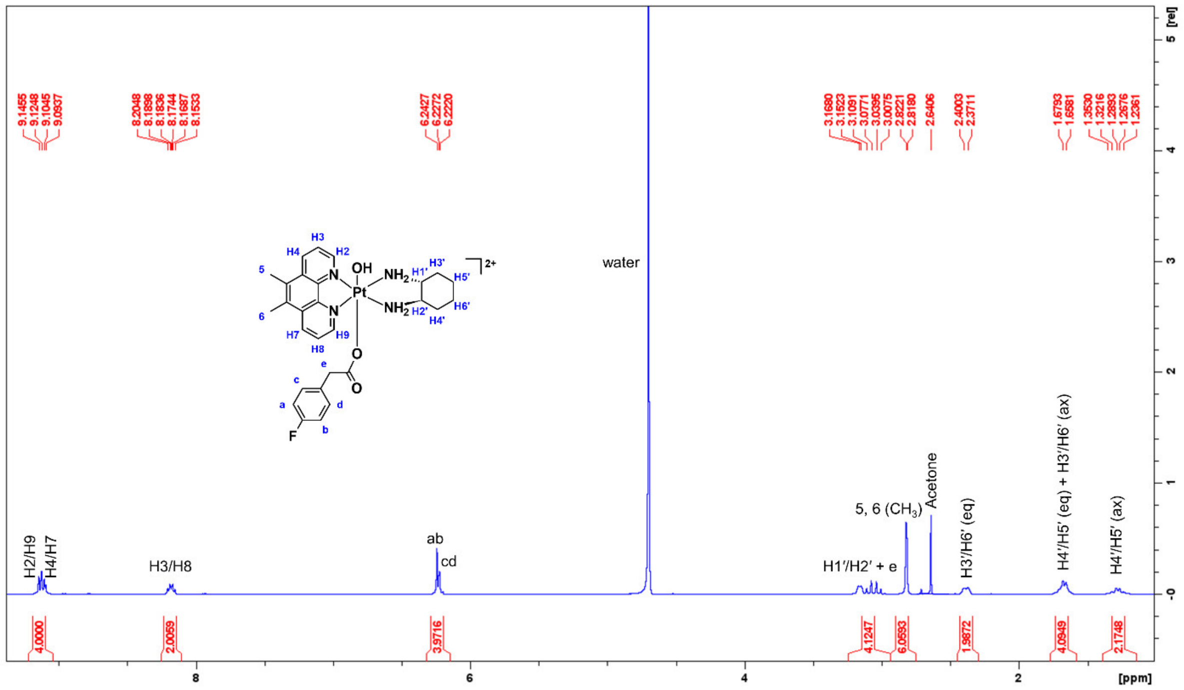

Figure S35: ^{1}H-NMR spectrum of **4** in D_2O obtained at 298 K Inset: structure of **4** with proton labelling system.

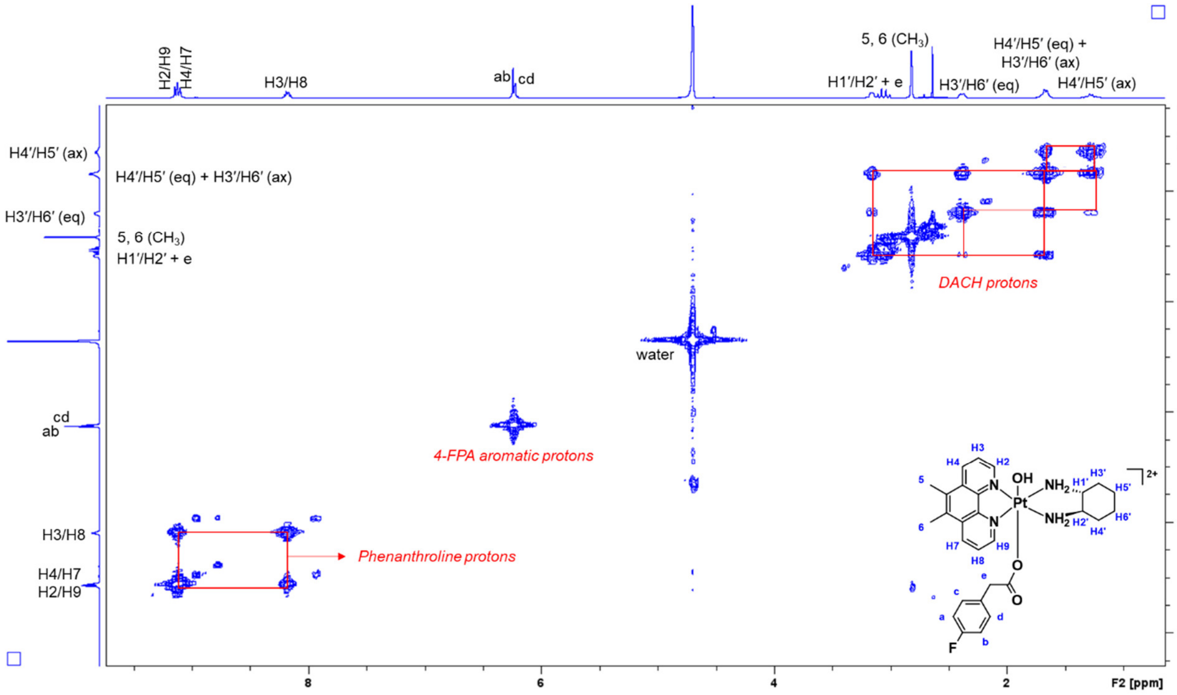

Figure S36: 2D-COSY spectrum of **4** in D_2O obtained at 298 K. Inset: structure of **4** with proton labelling system.

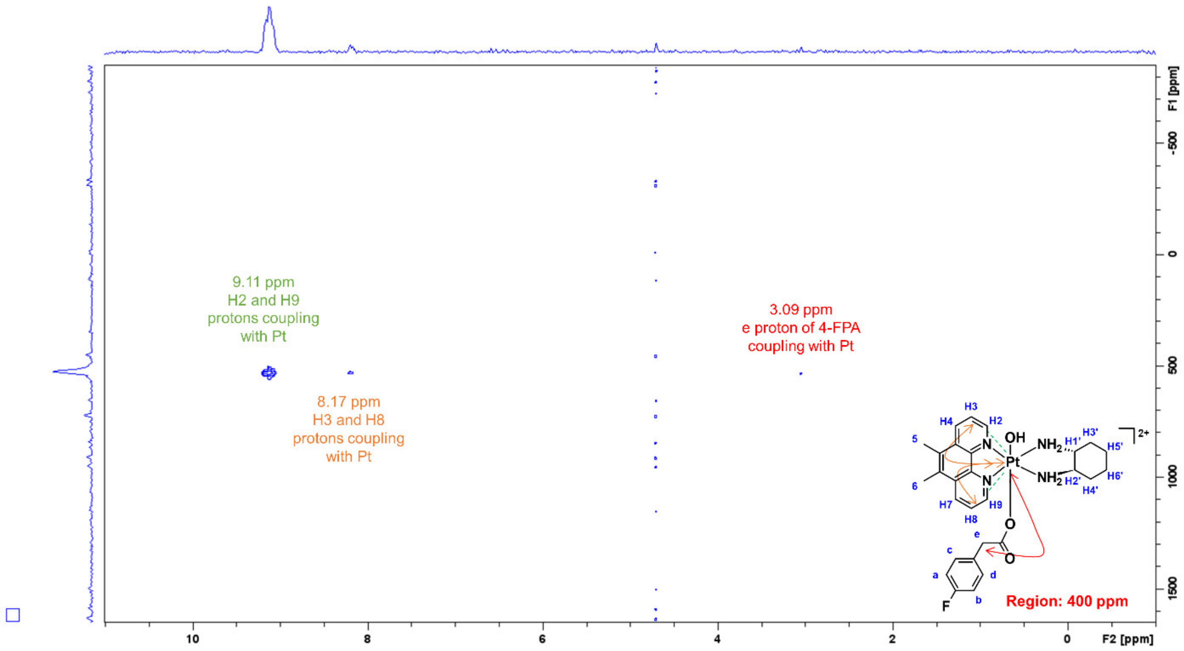

Figure S37: ^{1}H-^{195}Pt-HMQC spectrum of **4** in D_2O obtained at 298 K. Region: 400 ppm. Inset: structure of **4** with proton labelling system and arrows that indicate coupling.

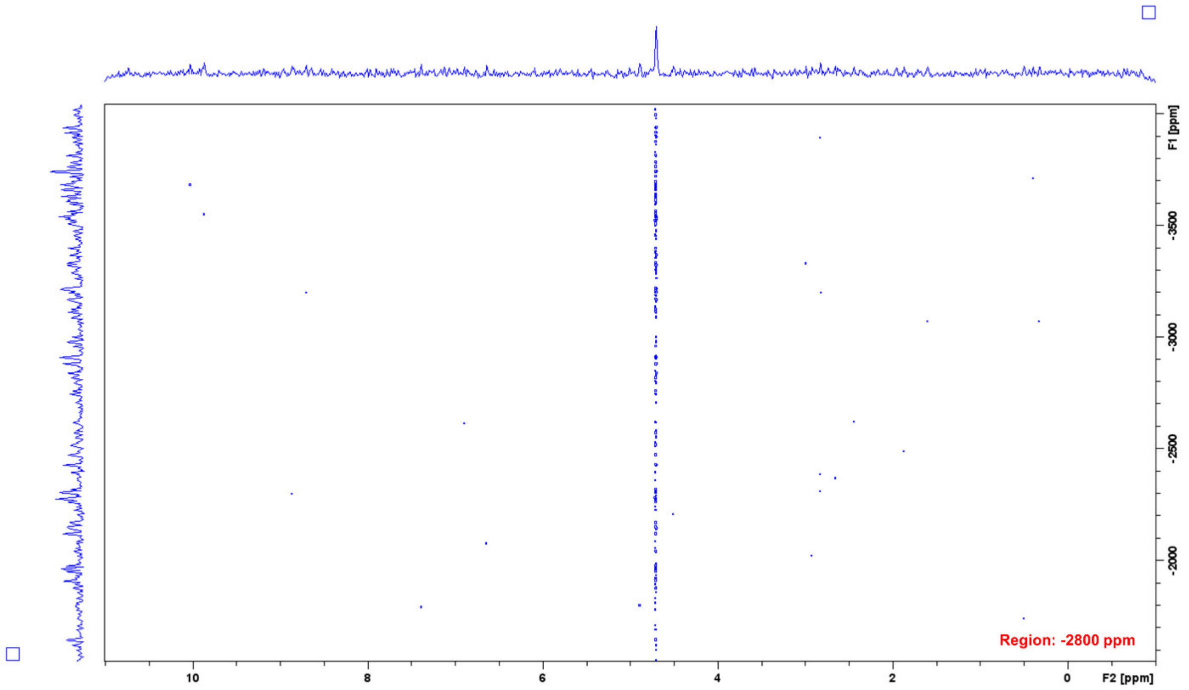

Figure S38: ^{1}H-^{195}Pt-HMQC spectrum of **4** in D_2O obtained at 298 K. Region: -2800 ppm. Absence of platinum(II) resonance.

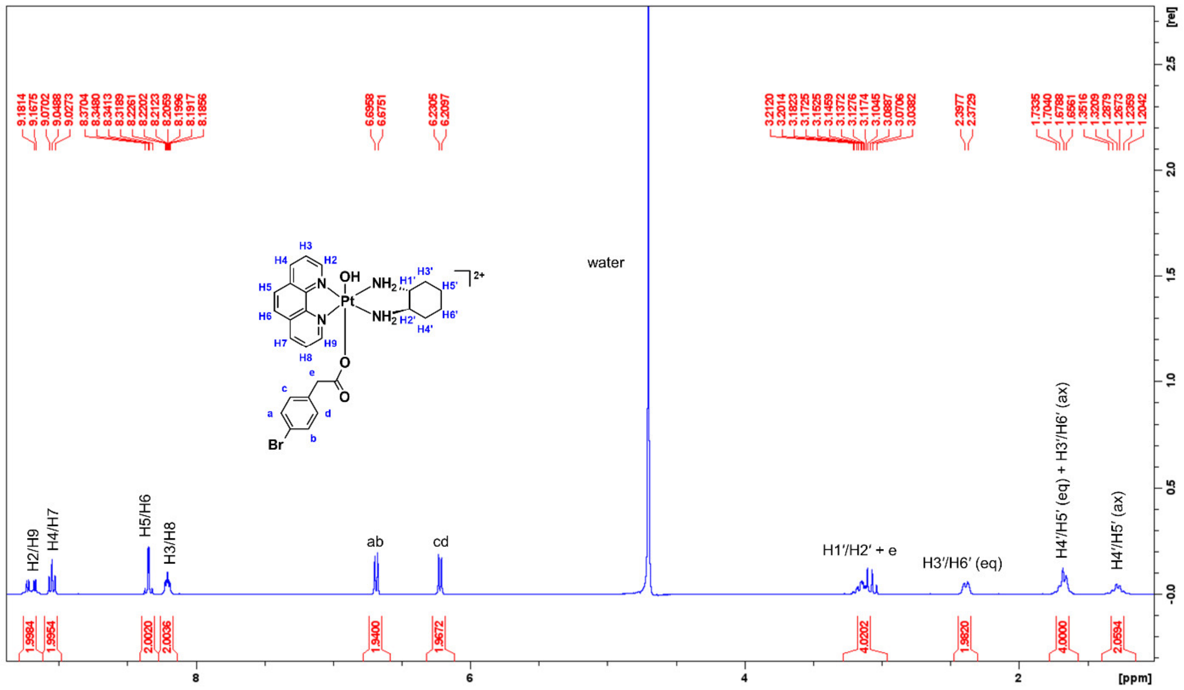

Figure S39: ^{1}H-NMR spectrum of **5** in D_2O obtained at 298 K Inset: structure of **5** with proton labelling system.

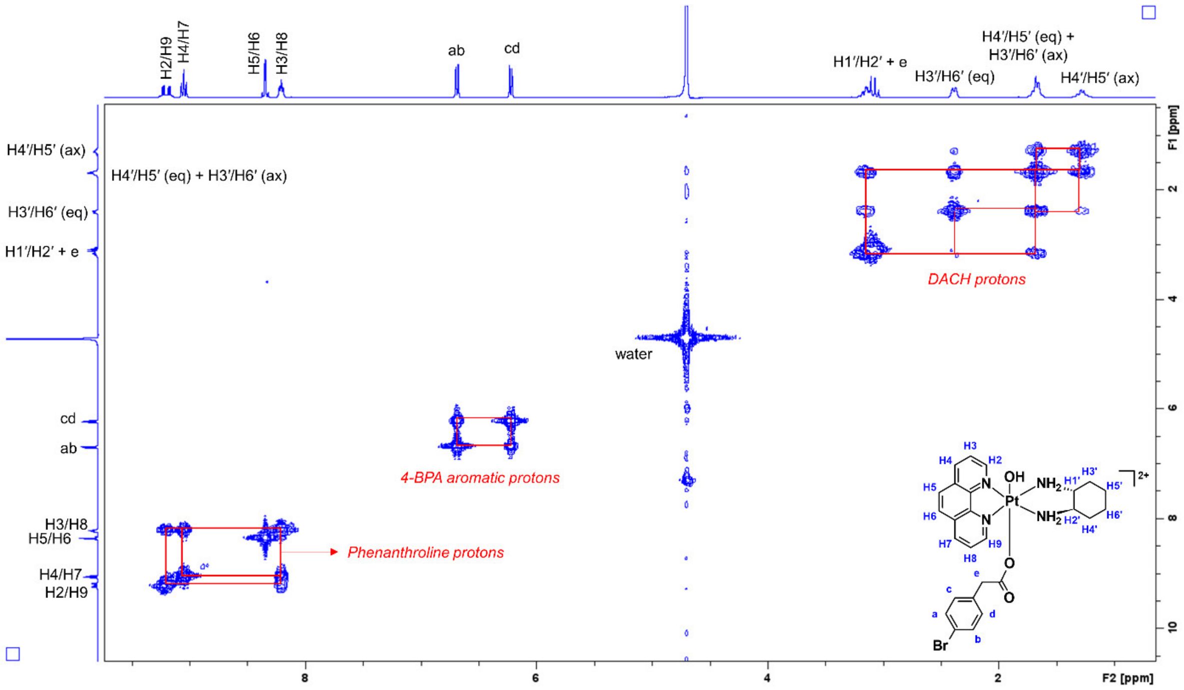

Figure S40: 2D-COSY spectrum of **5** in D_2O obtained at 298 K. Inset: structure of **5** with proton labelling system.

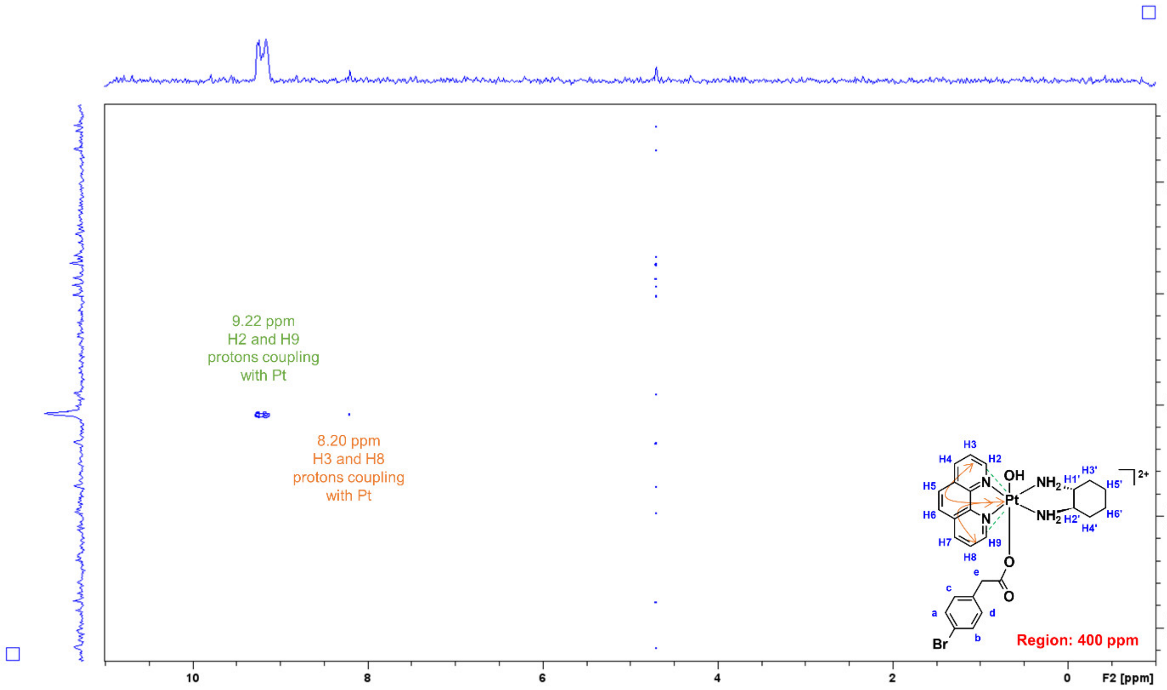

Figure S41: ^{1}H-^{195}Pt-HMQC spectrum of **5** in D_2O obtained at 298 K. Region: 400 ppm. Inset: structure of **5** with proton labelling system and arrows that indicate coupling.

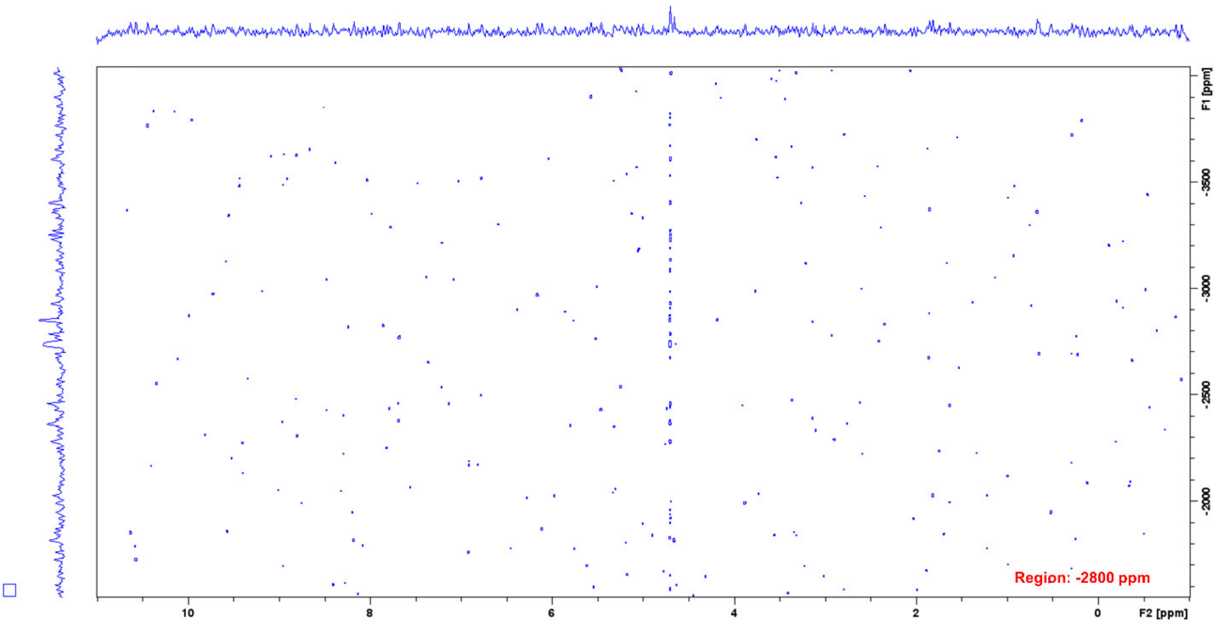

Figure S42: ^{1}H-^{195}Pt-HMQC spectrum of **5** in D_2O obtained at 298 K. Region: -2800 ppm. Absence of platinum(II) resonance.

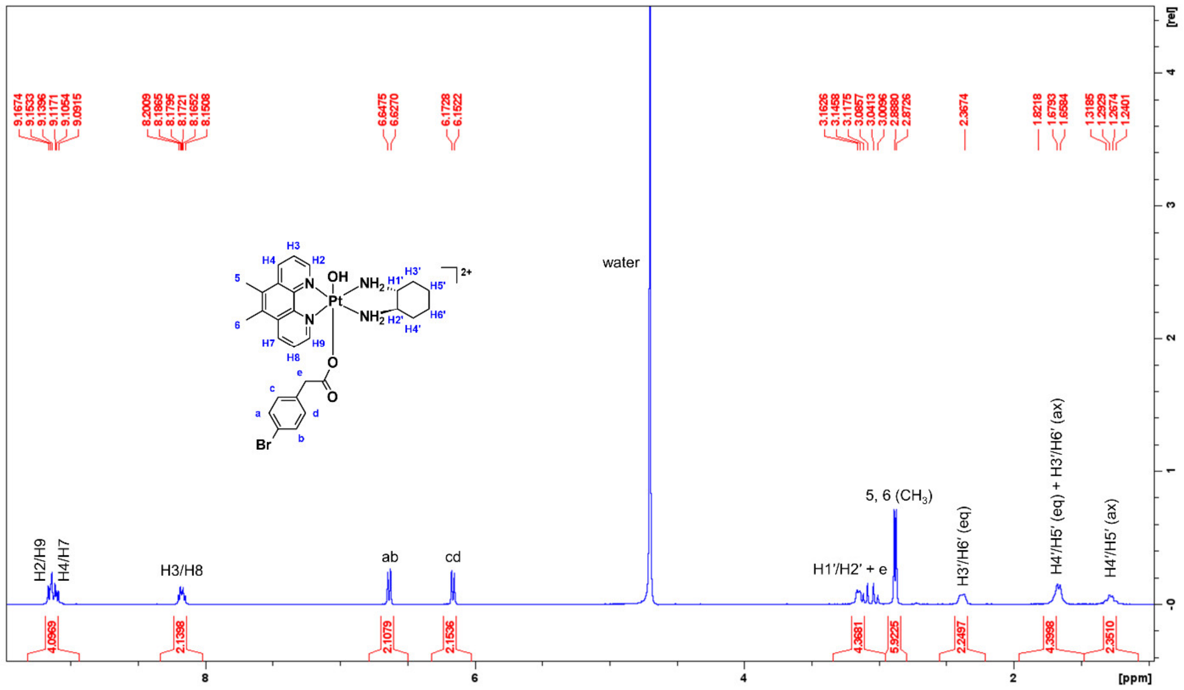

Figure S43: ^{1}H-NMR spectrum of **6** in D_2O obtained at 298 K Inset: structure of **6** with proton labelling system.

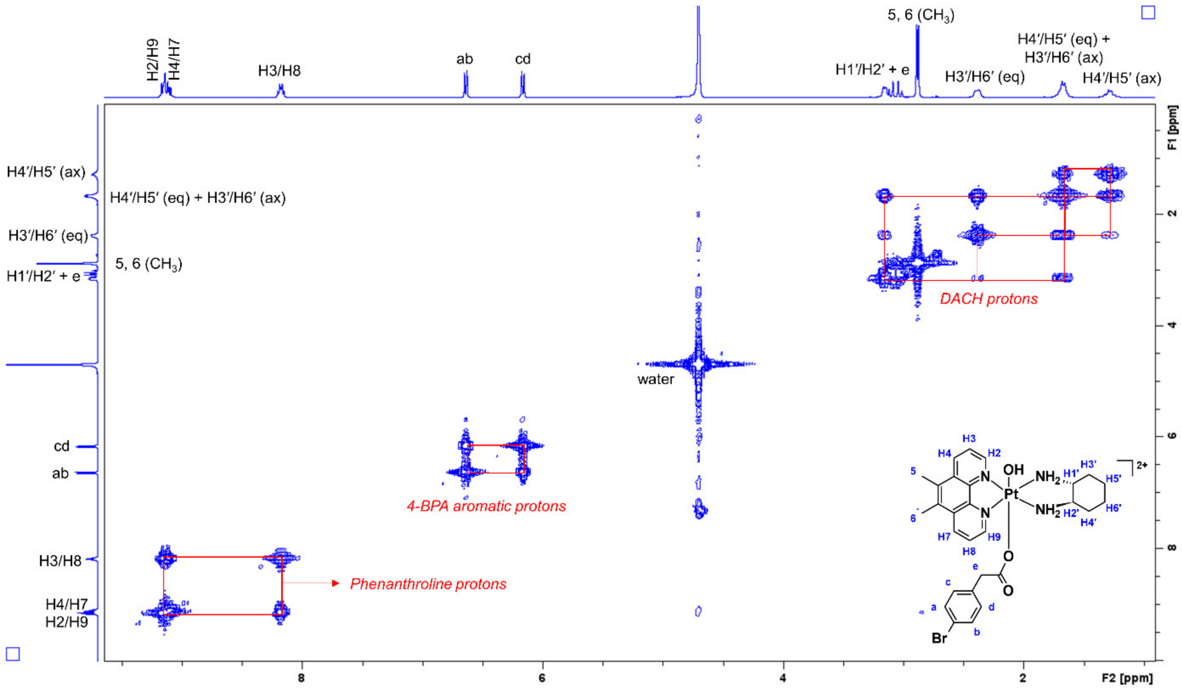

Figure S44: 2D-COSY spectrum of **6** in D_2O obtained at 298 K. Inset: structure of **6** with proton labelling system.

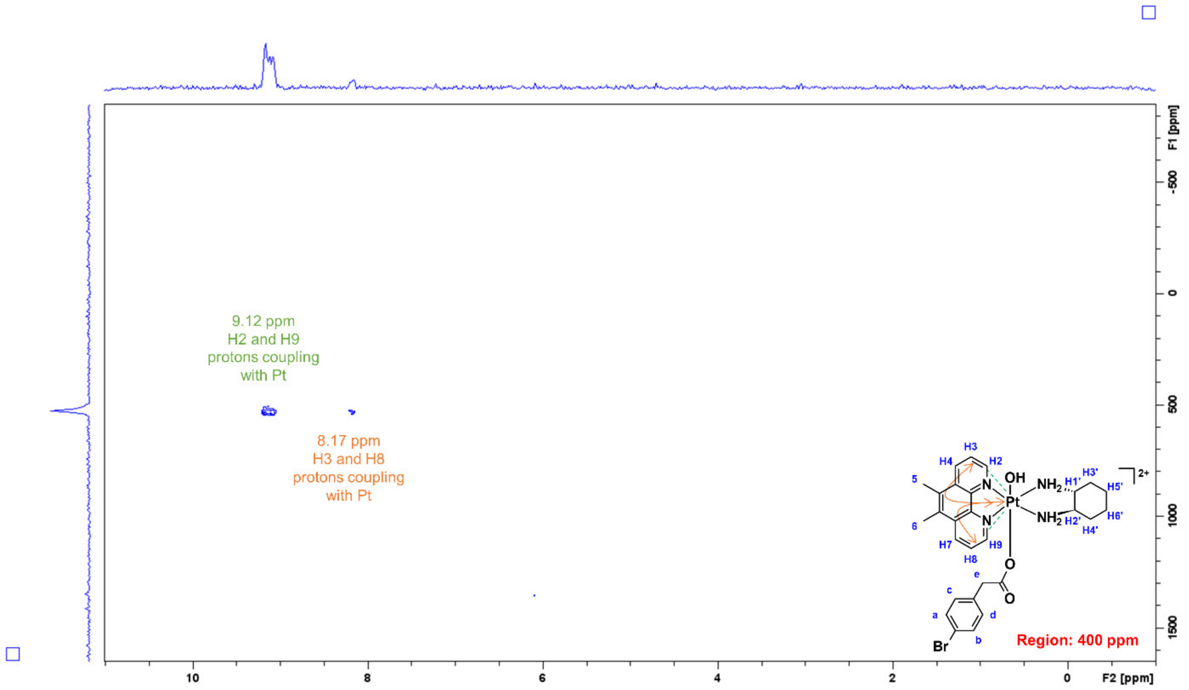

Figure S45: ^{1}H-^{195}Pt-HMQC spectrum of **6** in D_2O obtained at 298 K. Region: 400 ppm. Inset: structure of **6** with proton labelling system and arrows that indicate coupling.

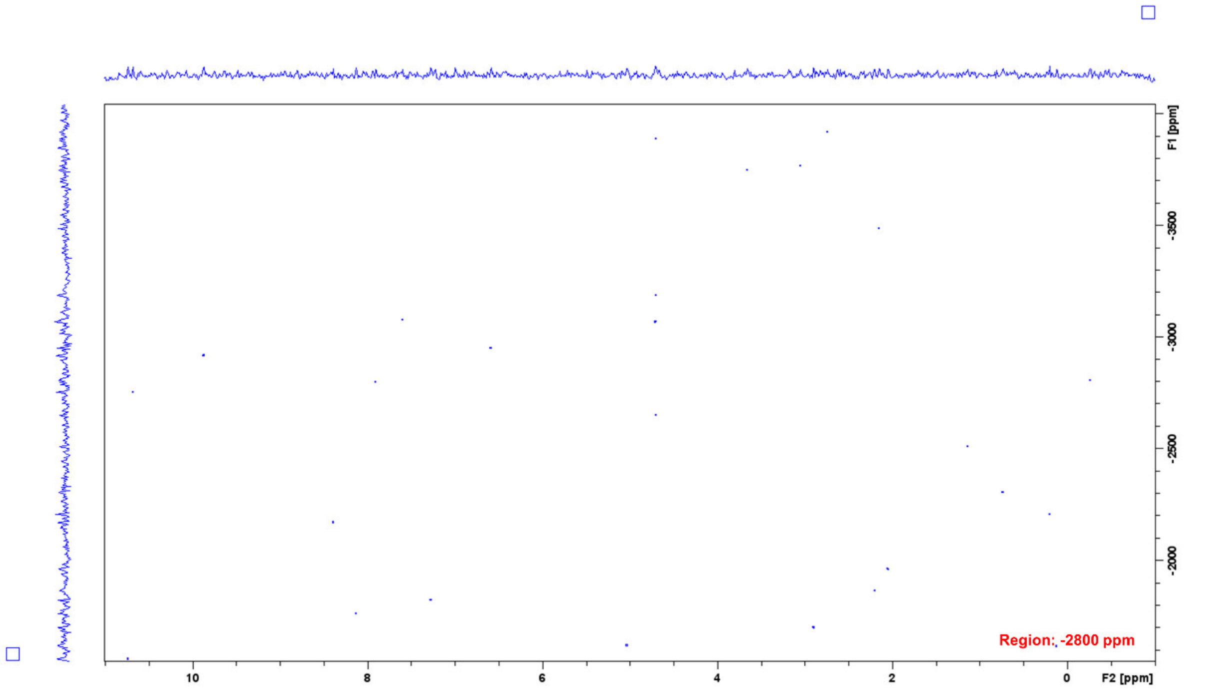

Figure S46: ^{1}H-^{195}Pt-HMQC spectrum of **6** in D_2O obtained at 298 K. Region: -2800 ppm. Absence of platinum(II) resonance.

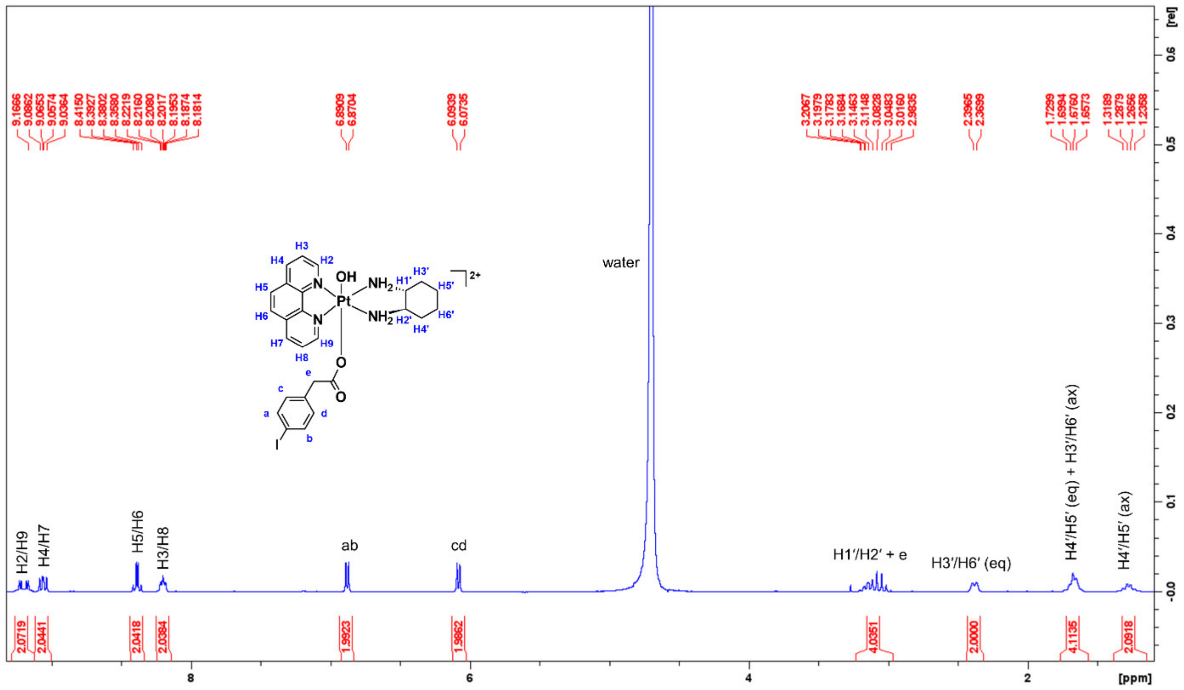

Figure S47: ^{1}H-NMR spectrum of **7** in D_2O obtained at 298 K Inset: structure of **7** with proton labelling system.

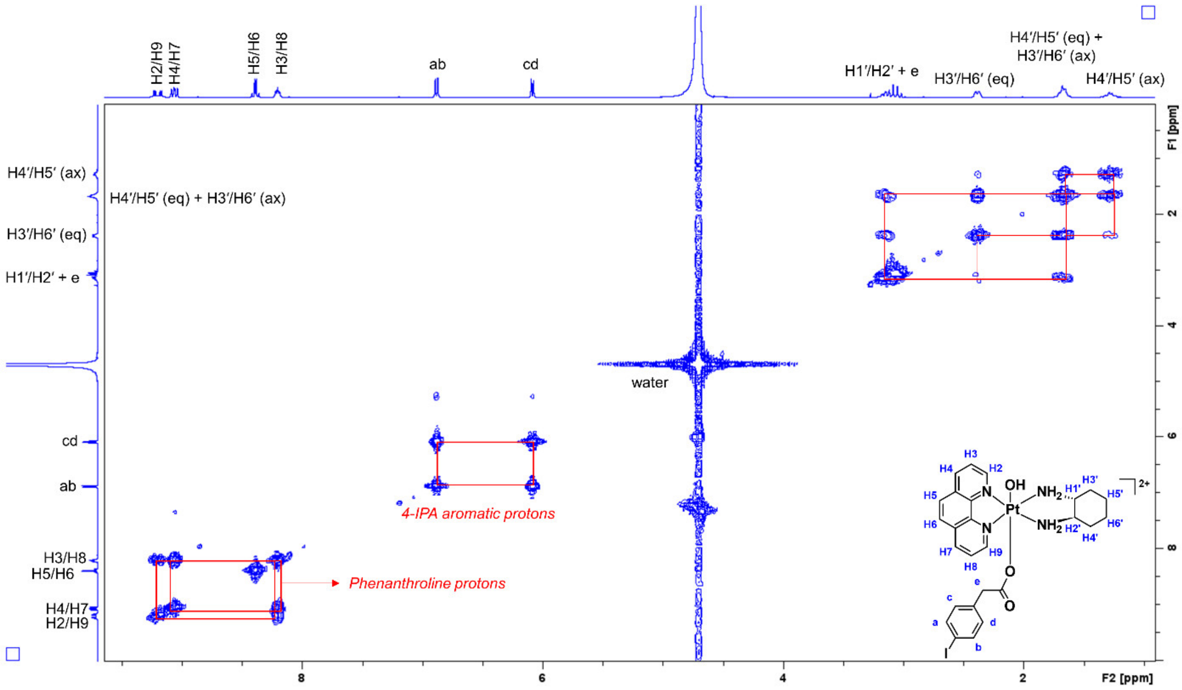

Figure S48: 2D-COSY spectrum of **7** in D_2O obtained at 298 K. Inset: structure of **7** with proton labelling system.

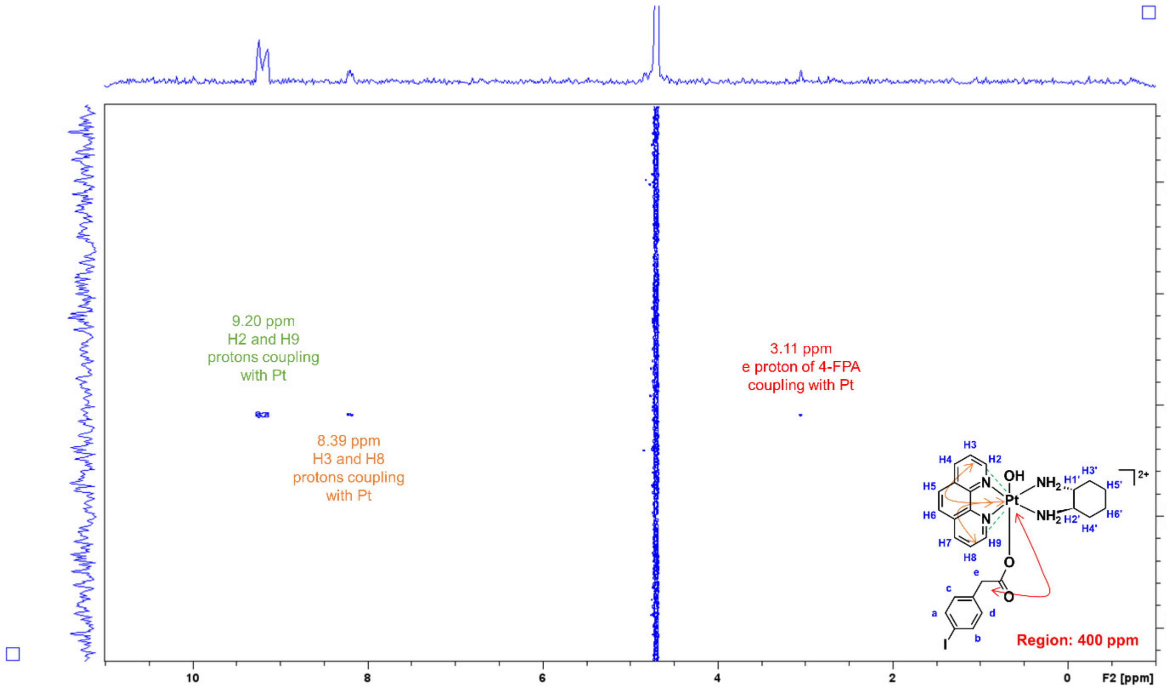

Figure S49: ^{1}H-^{195}Pt-HMQC spectrum of **7** in D_2O obtained at 298 K. Region: 400 ppm. Inset: structure of **7** with proton labelling system and arrows that indicate coupling.

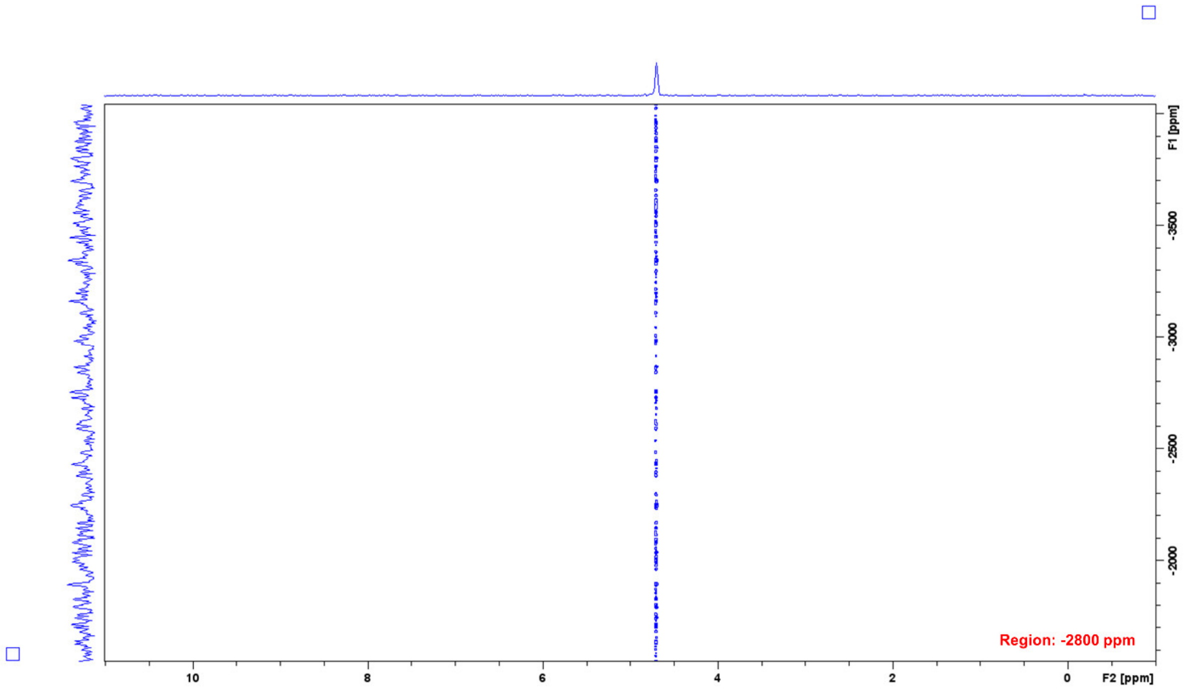

Figure S50: ^{1}H-^{195}Pt-HMQC spectrum of **7** in D_2O obtained at 298 K. Region: -2800 ppm. Absence of platinum(II) resonance.

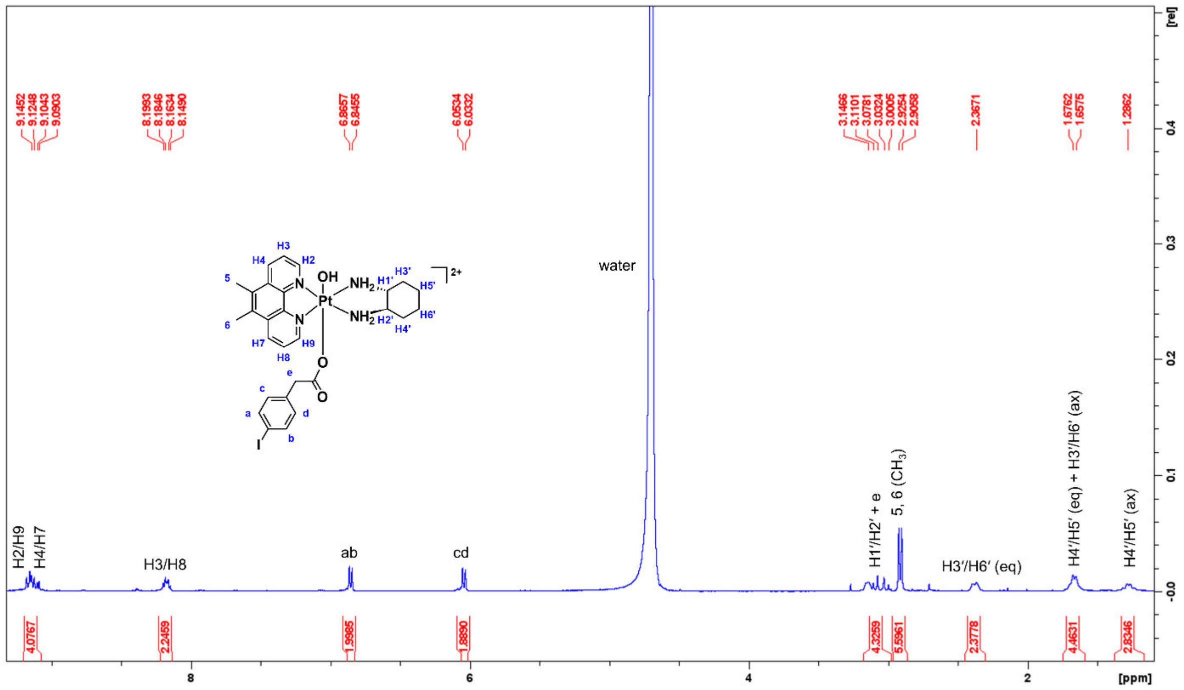

Figure S51: ^{1}H-NMR spectrum of **8** in D_2O obtained at 298 K Inset: structure of **8** with proton labelling system.

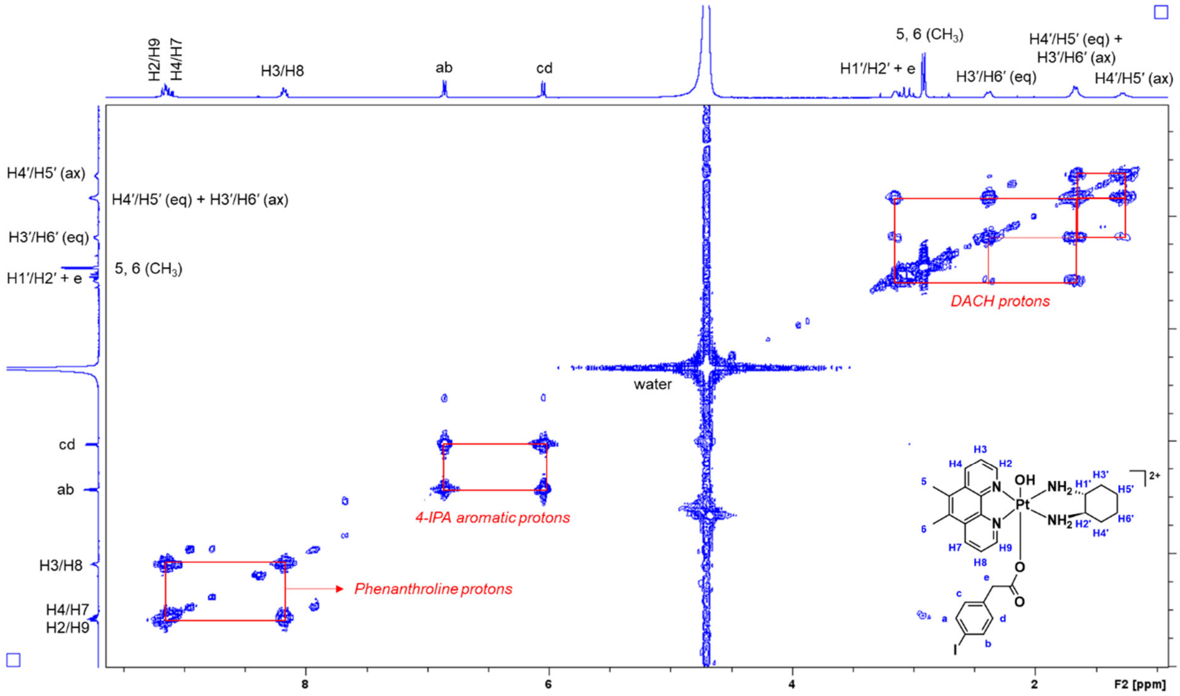

Figure S52: 2D-COSY spectrum of **8** in D_2O obtained at 298 K. Inset: structure of **8** with proton labelling system.

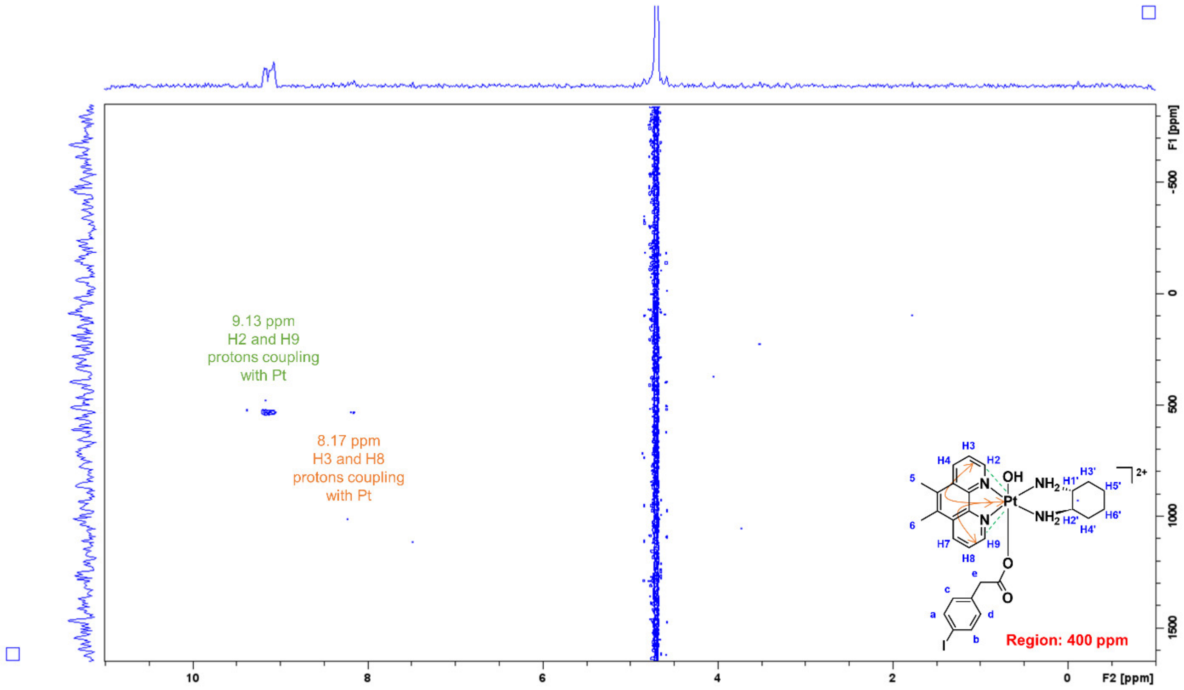

Figure S53: ^1H-^{195}Pt-HMQC spectrum of **8** in D_2O obtained at 298 K. Region: 400 ppm. Inset: structure of **8** with proton labelling system and arrows that indicate coupling.

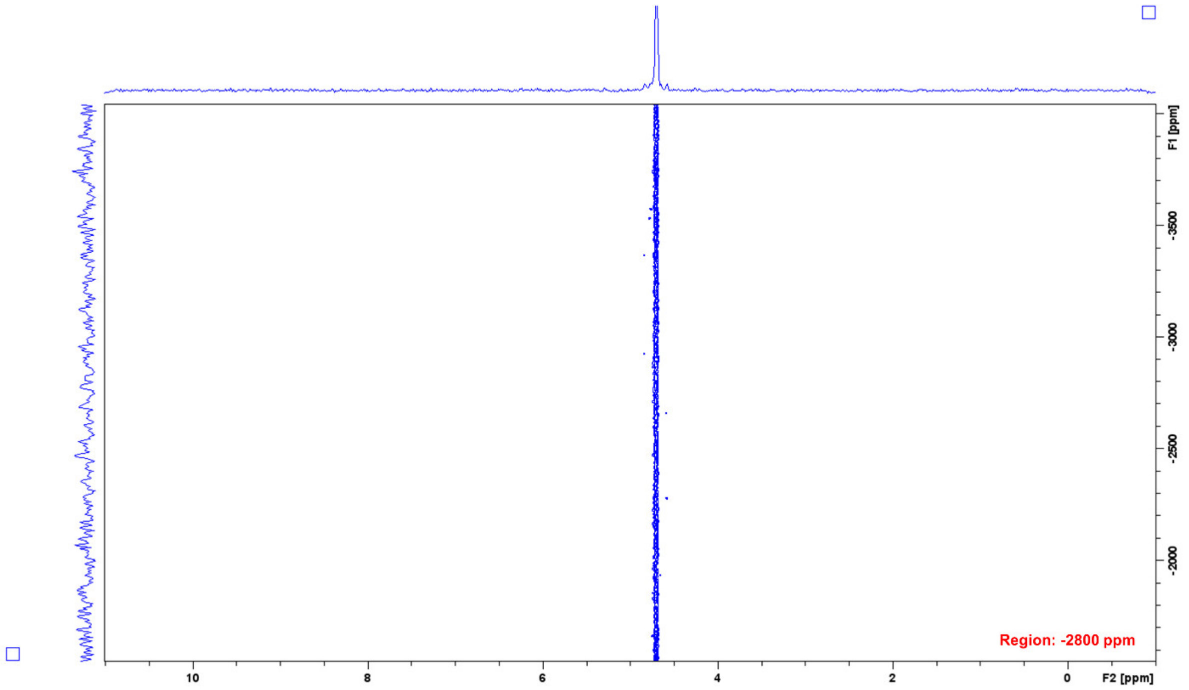

Figure S54: ^{1}H-^{195}Pt-HMQC spectrum of **8** in D_2O obtained at 298 K. Region: -2800 ppm. Absence of platinum(II) resonance.

UV spectra of platinum(IV) complexes of 1 – 8

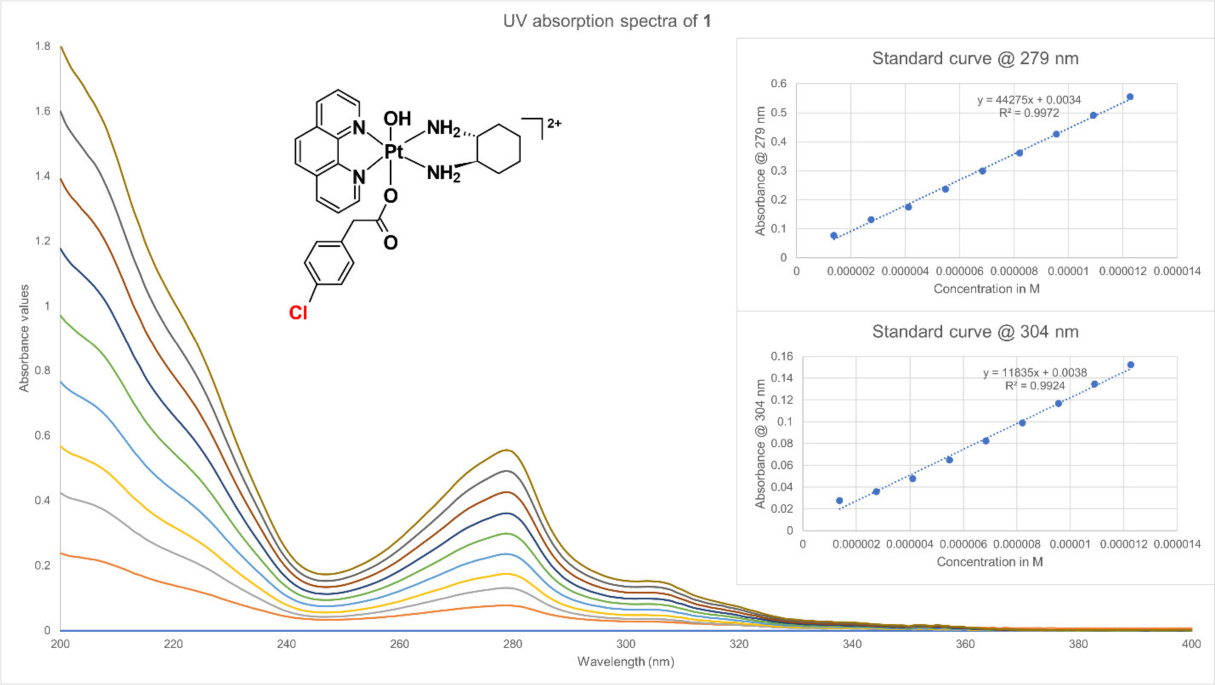

Figure S55: UV spectra of **1** obtained at 298 K. Inset: structure of **1** and generated plot curves within the wavelengths 279 and 304 nm.

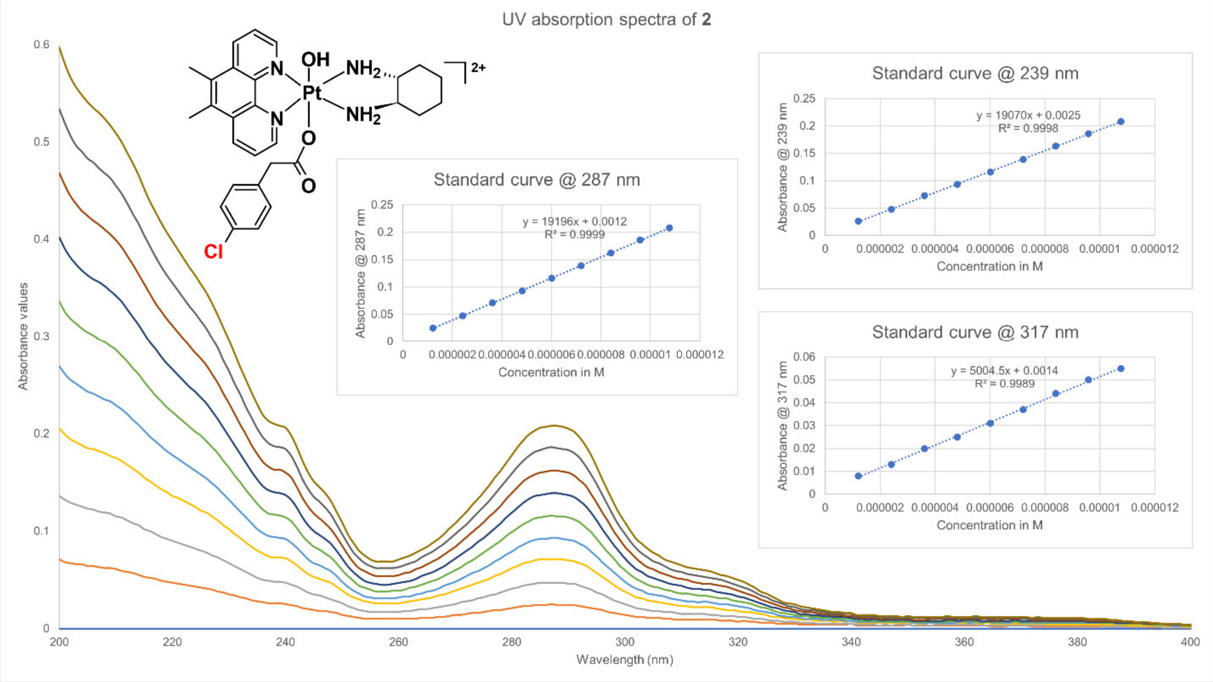

Figure S56: UV spectra of **2** obtained at 298 K. Inset: structure of **2** and generated plot curves within the wavelengths 239, 287 and 317 nm.

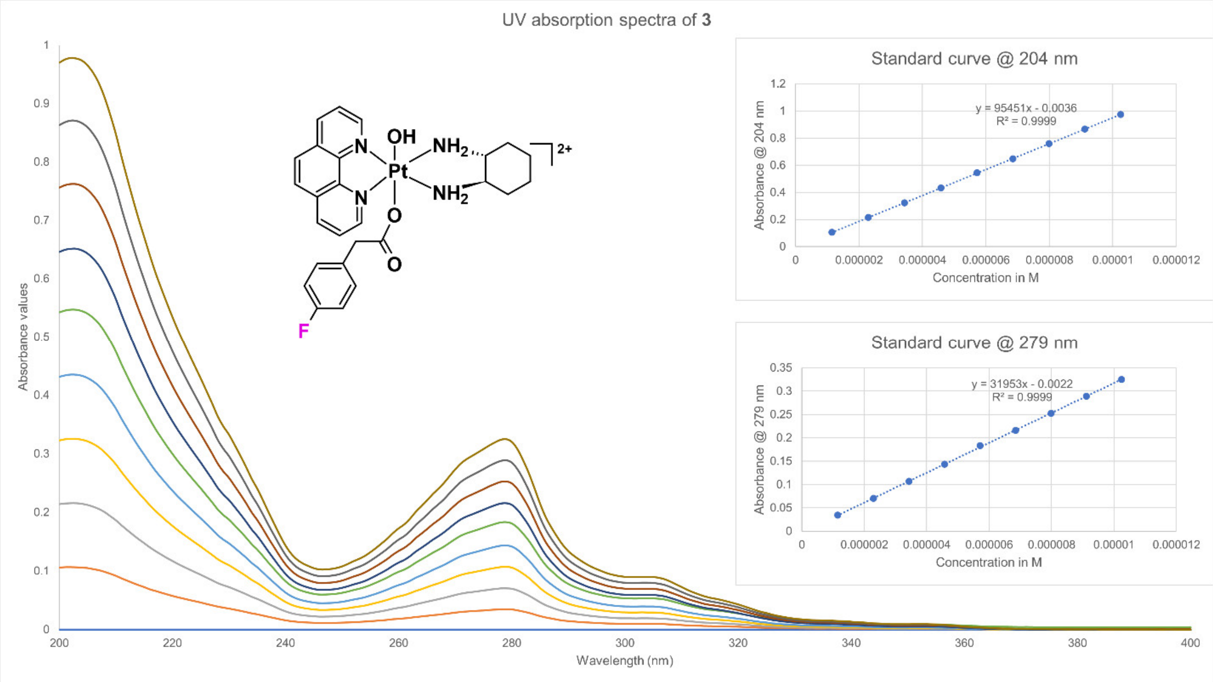

Figure S57: UV spectra of **3** at 298 K. Inset: structure of **3** and generated plot curves within the wavelengths 204 and 279 nm.

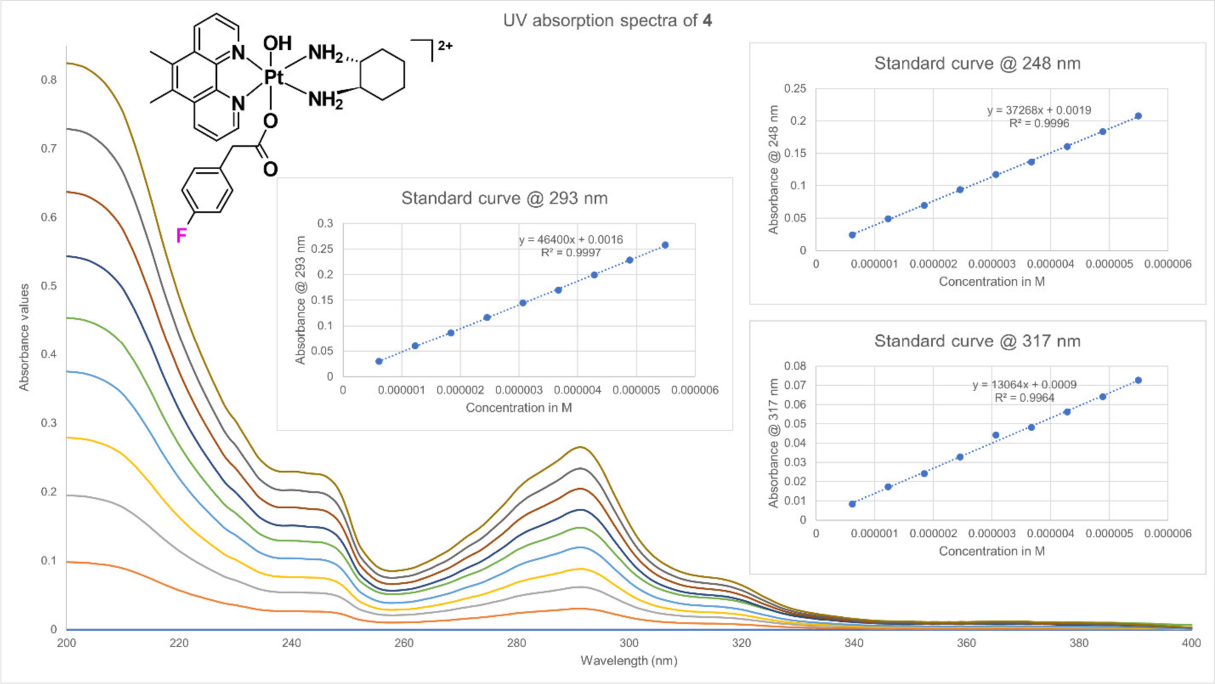

Figure S58: UV spectra of **4** obtained at 298 K. Inset: structure of **4** and generated plot curves within the wavelengths 248, 293 and 317 nm.

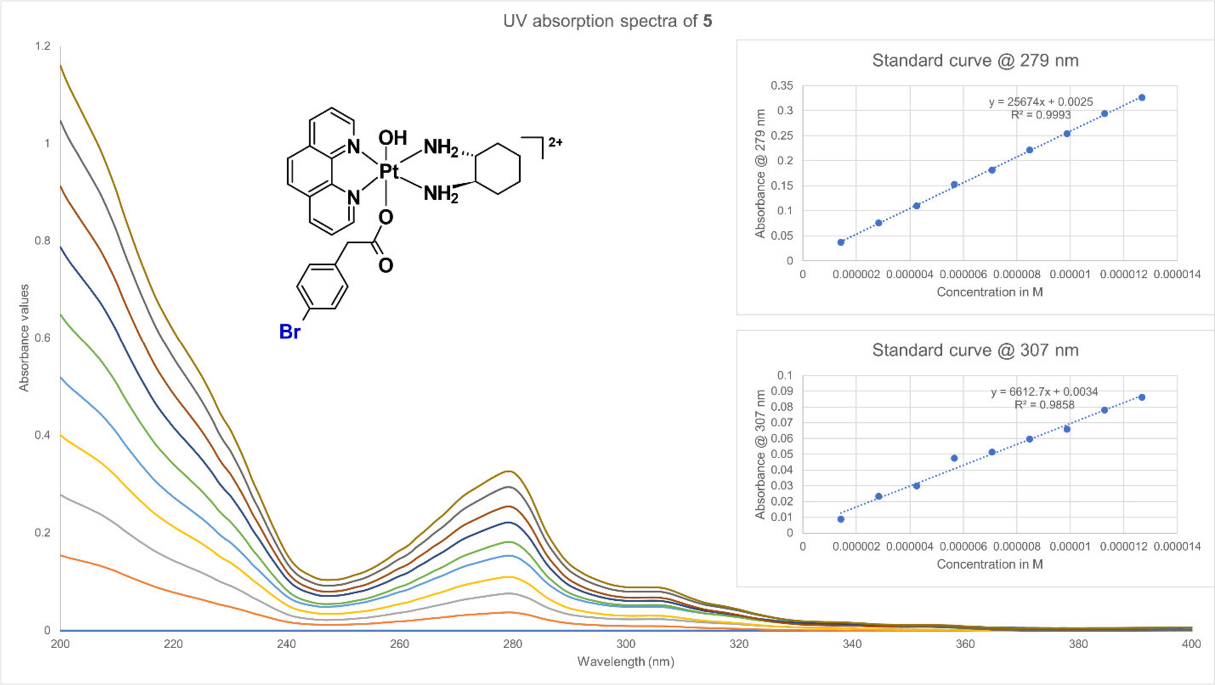

Figure S59: UV spectra of **5** obtained at 298 K. Inset: structure of **5** and generated plot curves within the wavelengths 279 and 307 nm.

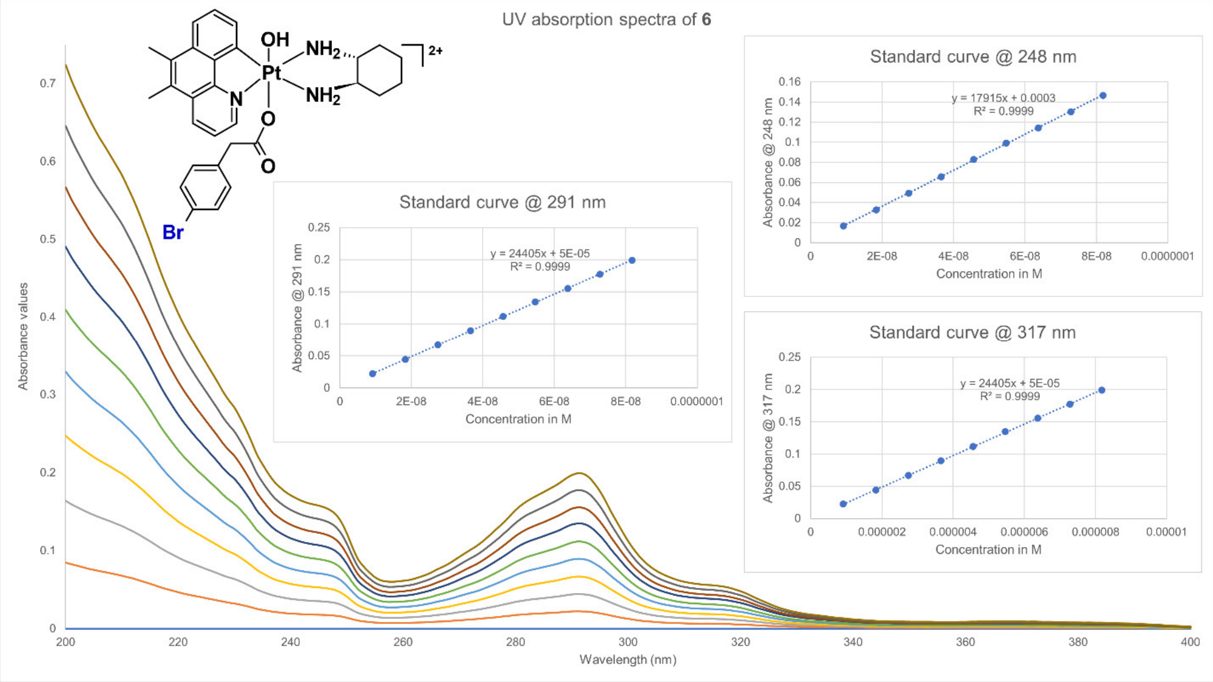

Figure S60: UV spectra of **6** obtained at 298 K. Inset: structure of **6** and generated plot curves within the wavelengths 248, 291 and 317 nm.

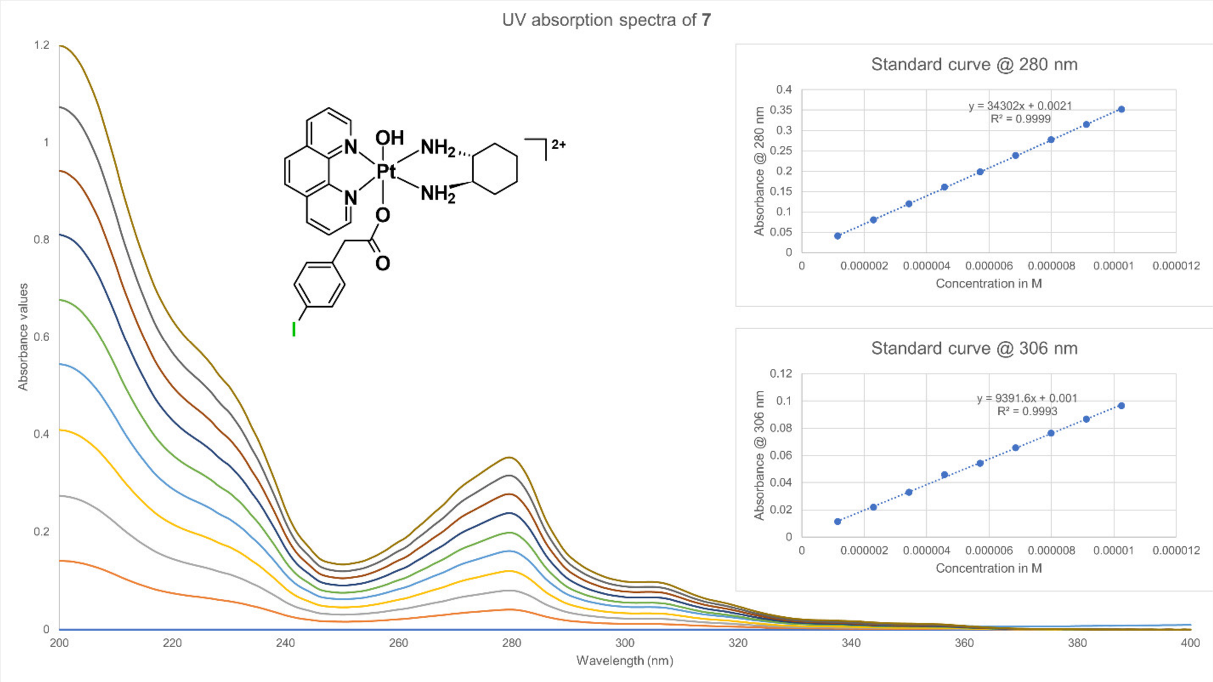

Figure S61: UV spectra of **7** obtained at 298 K. Inset: structure of **7** and generated plot curves within the wavelengths 280 and 306 nm.

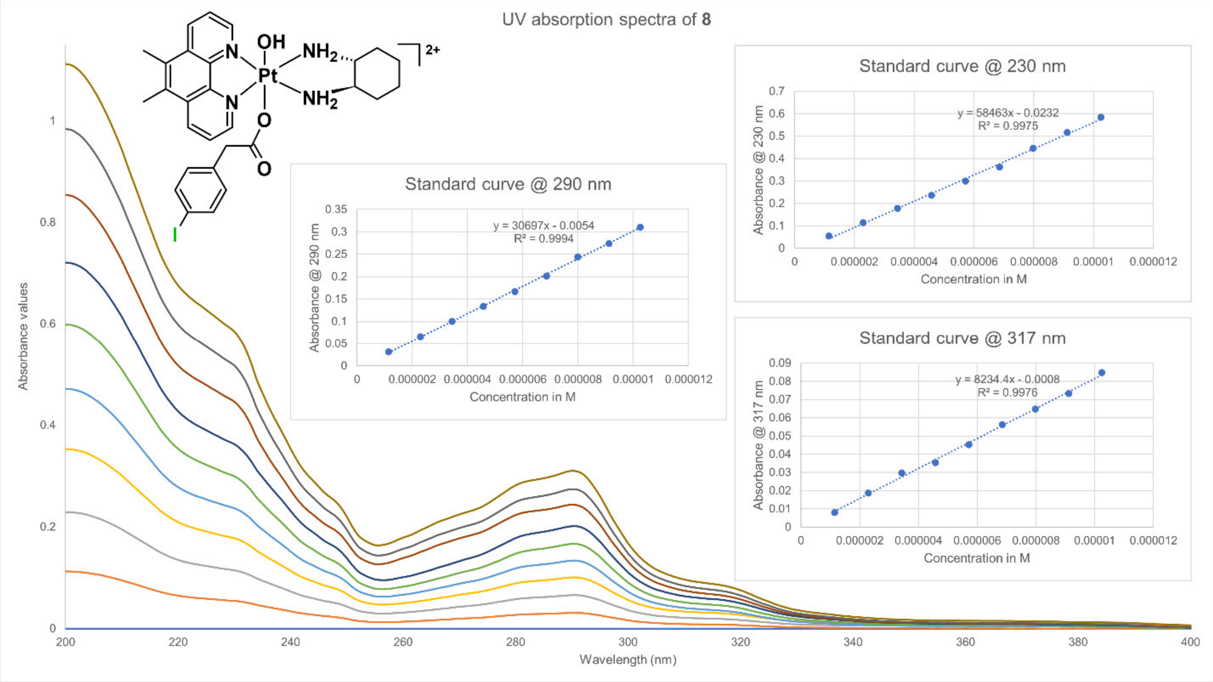

Figure S62: UV spectra of **8** obtained at 298 K. Inset: structure of **8** and generated plot curves within the wavelengths 230, 290 and 317 nm.

CD spectra of 1 – 8

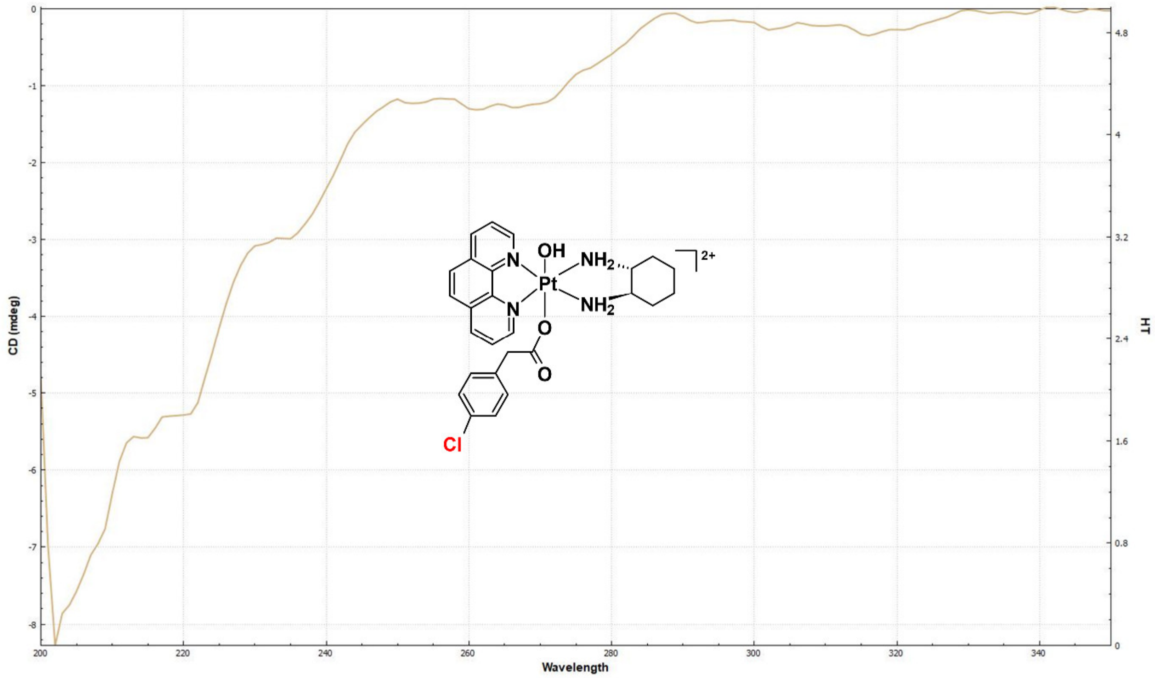

Figure S63: CD spectrum of **1** obtained at 298 K. Inset: structure of **1**.

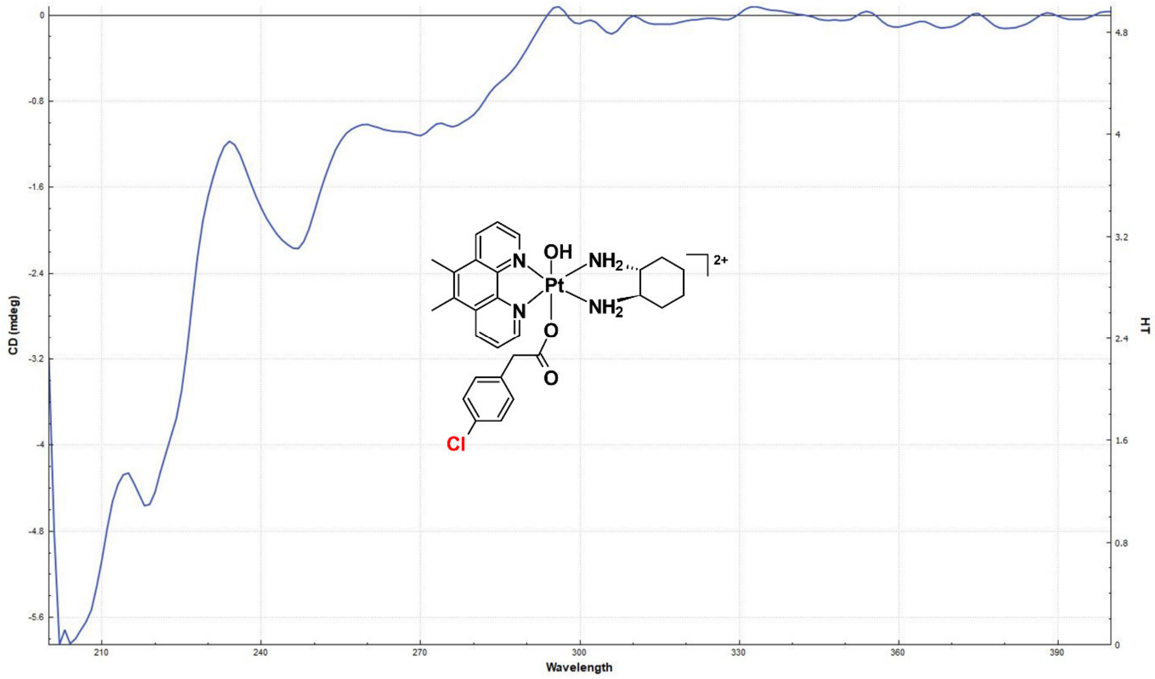

Figure S64: CD spectrum of **2** obtained at 298 K. Inset: structure of **2**.

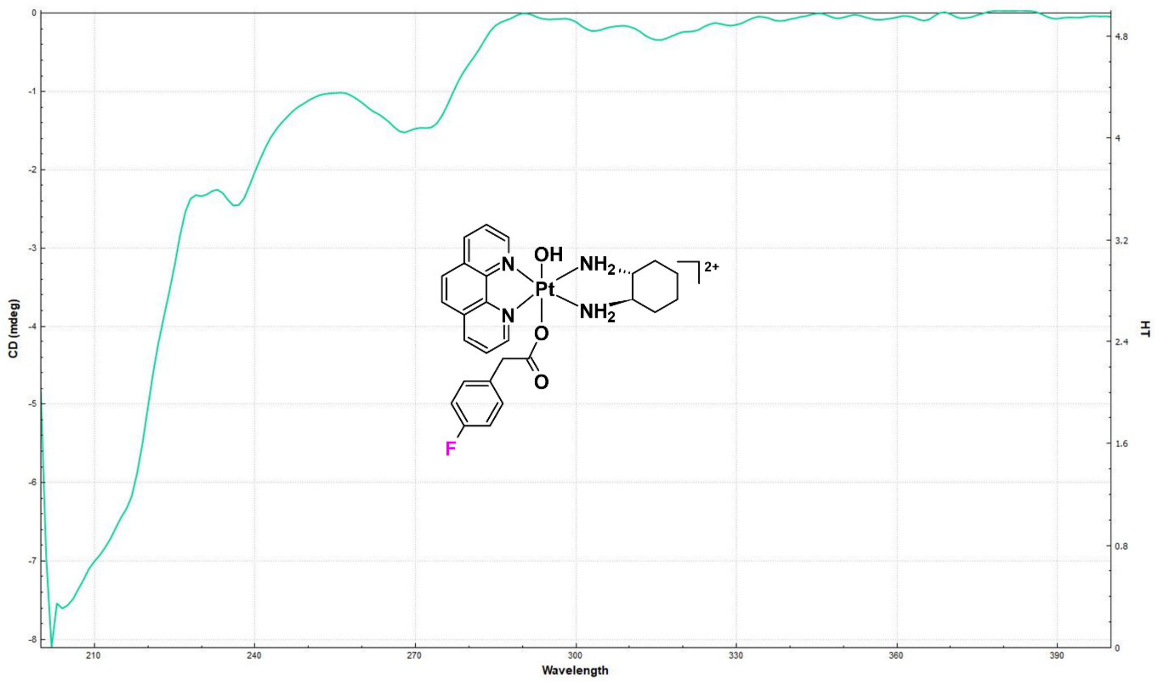

Figure S65: CD spectrum of **3** obtained at 298 K. Inset: structure of **3**.

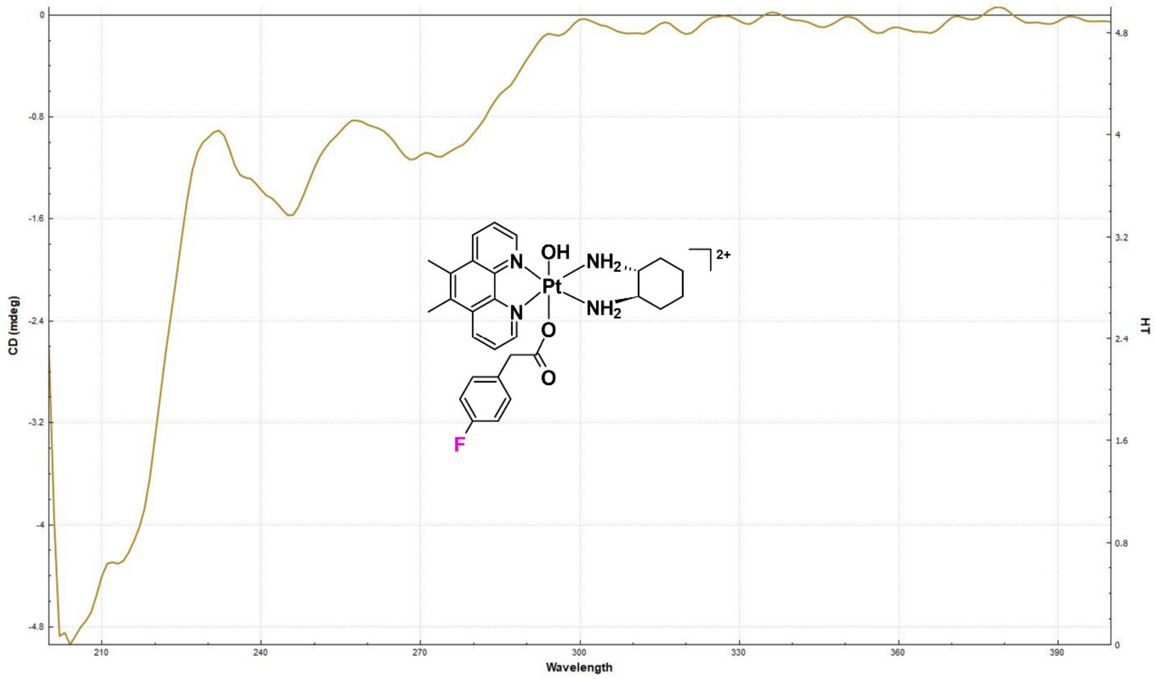

Figure S66: CD spectrum of **4** obtained at 298 K. Inset: structure of **4**.

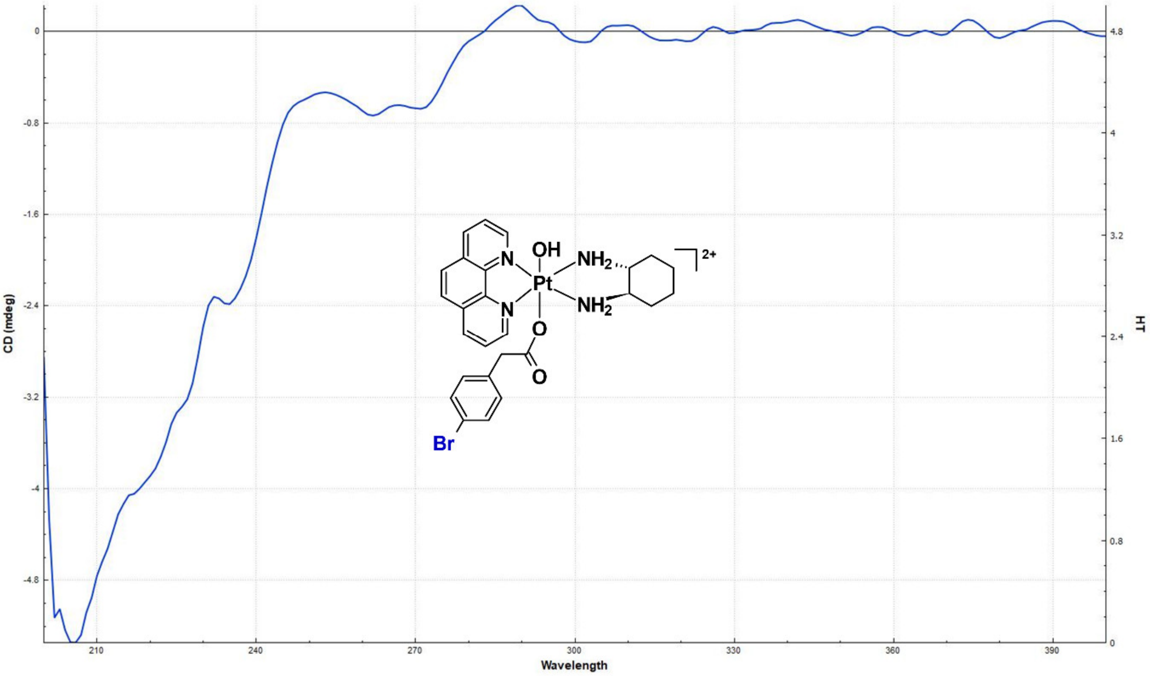

Figure S67: CD spectrum of **5** obtained at 298 K. Inset: structure of **5**.

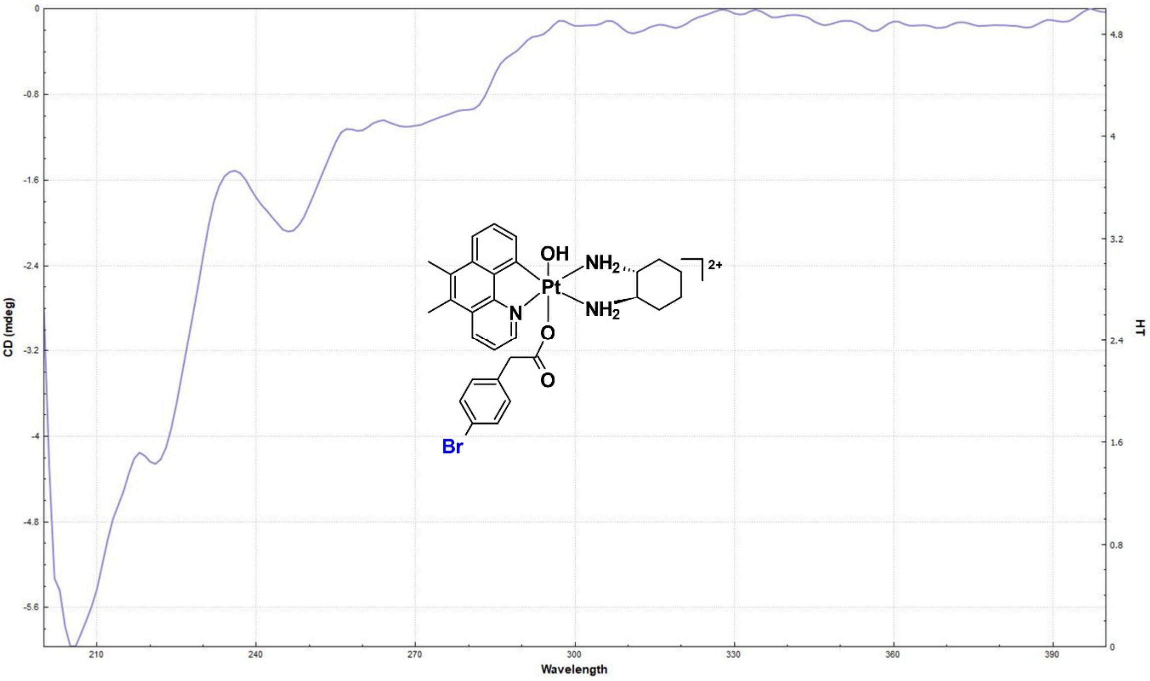

Figure S68: CD spectrum of **6** obtained at 298 K. Inset: structure of **6**.

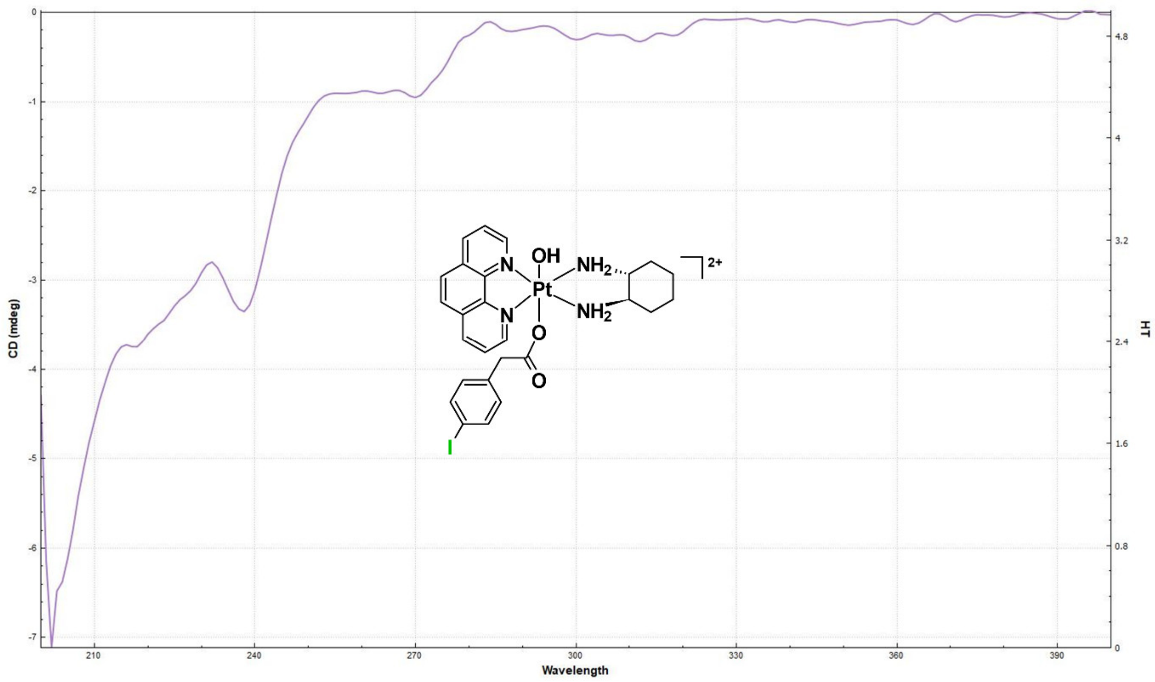

Figure S69: CD spectrum of **7** obtained at 298 K. Inset: structure of **7**.

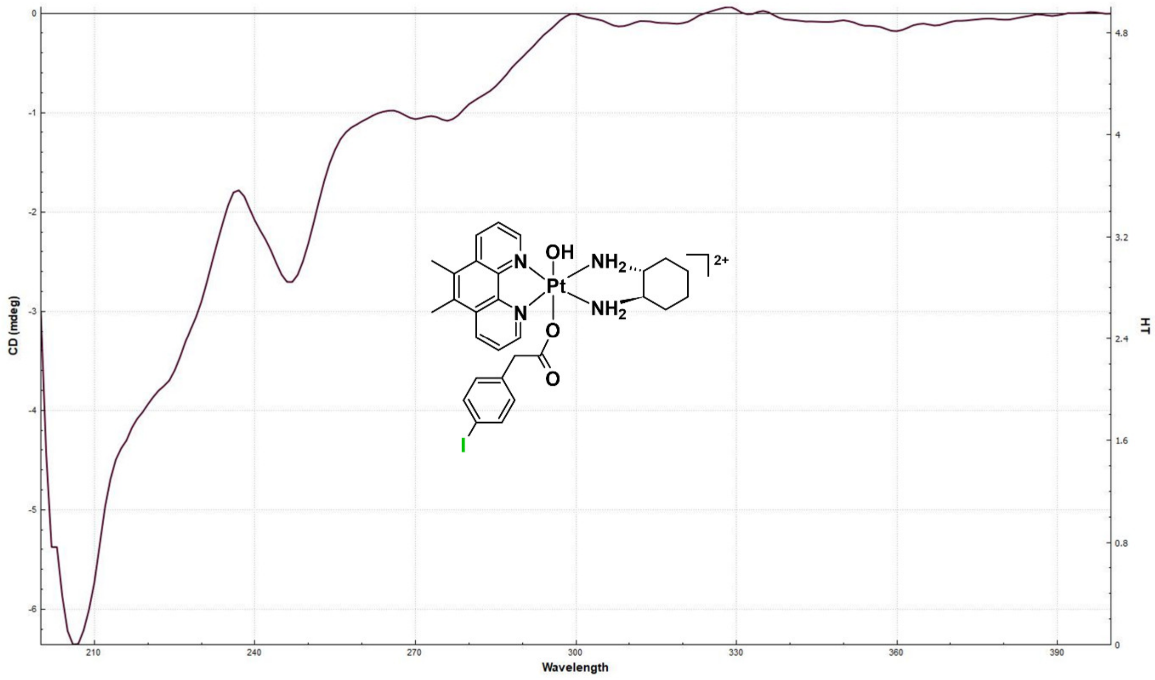

Figure S70: CD spectrum of **8** obtained at 298 K. Inset: structure of **8**.

ESI-MS spectra of 1 – 8

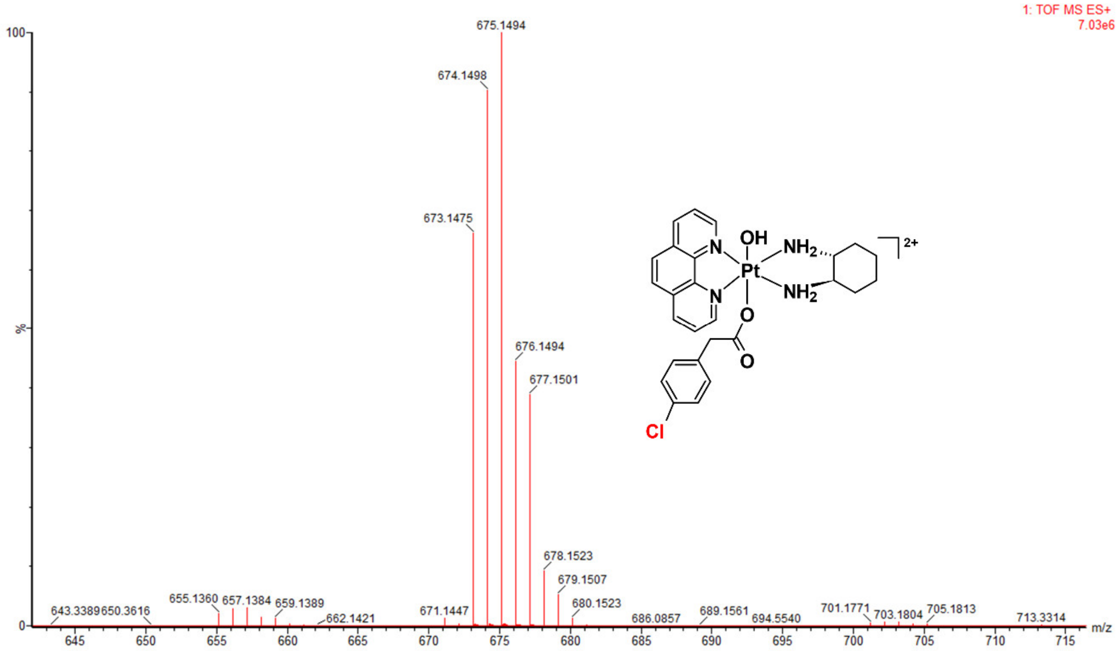

Figure S71: ESI-MS spectrum of **1** obtained at 298 K, using a Waters SYNAPT G2-Si quadruple time-of-flight (QTOF) HDMS. Inset: structure of **1**.

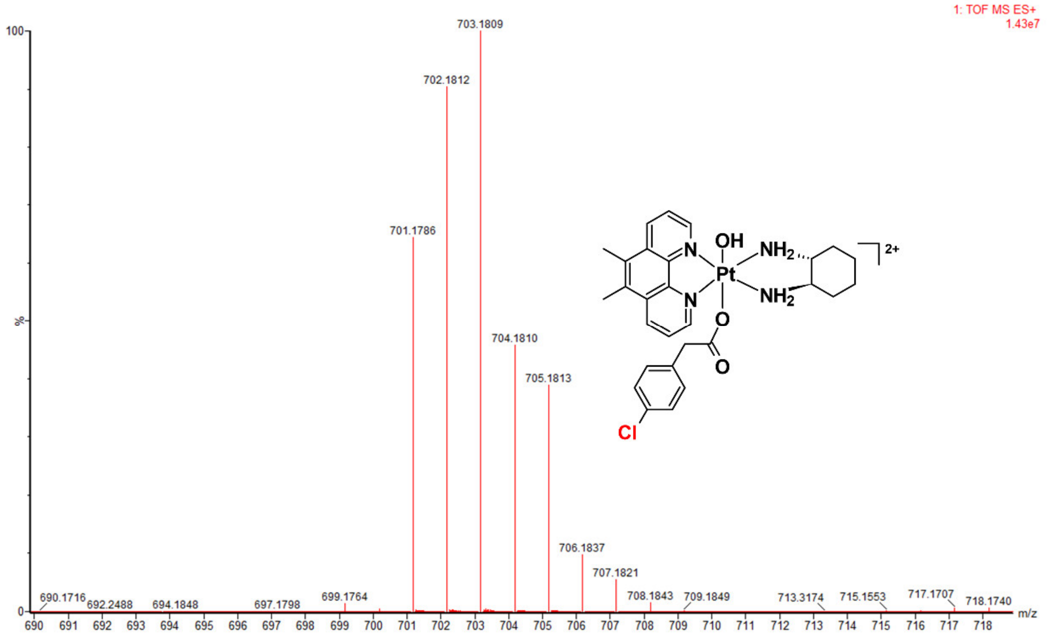

Figure S72: ESI-MS spectrum of **2** obtained at 298 K, using a Waters SYNAPT G2-Si quadruple time-of-flight (QTOF) HDMS. Inset: structure of **2**.

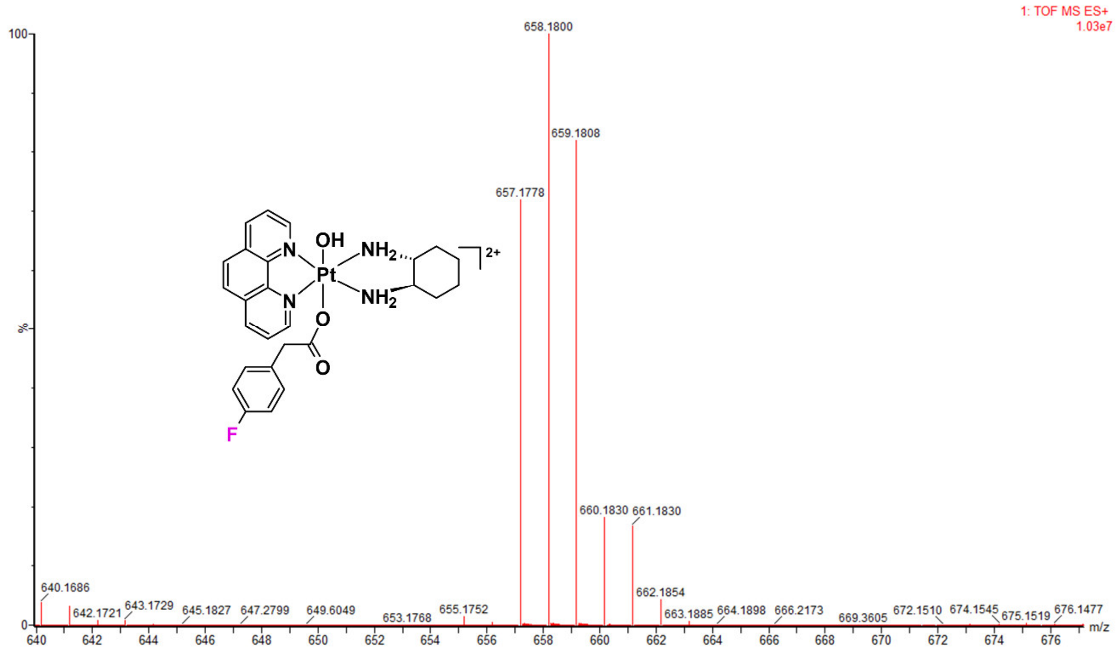

Figure S73: ESI-MS spectrum of **3** obtained at 298 K, using a Waters SYNAPT G2-Si quadruple time-of-flight (QTOF) HDMS. Inset: structure of **3**.

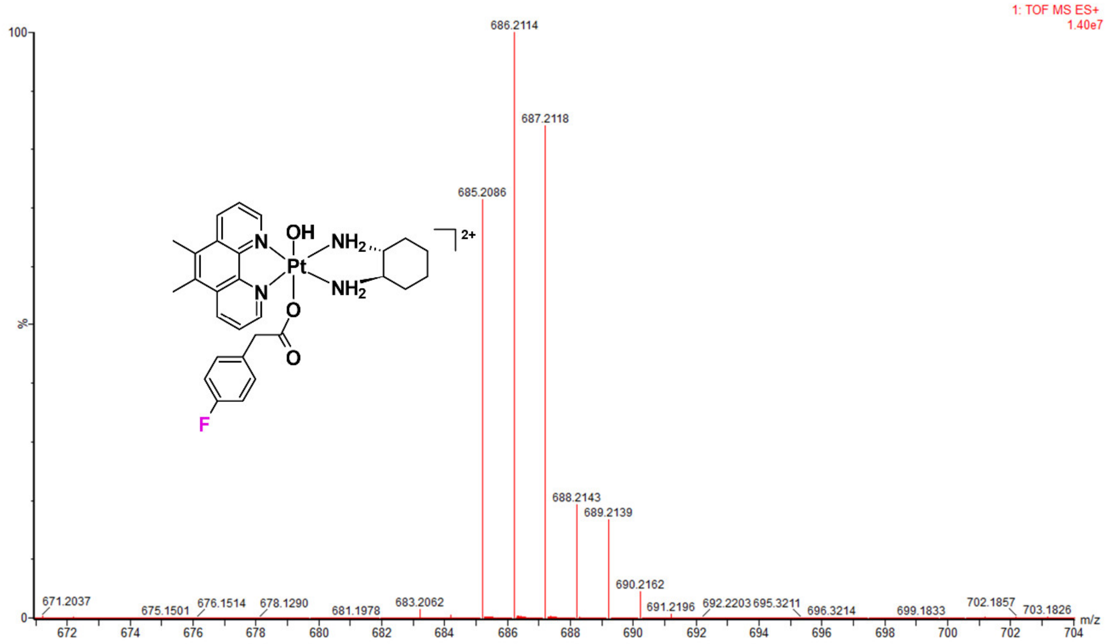

Figure S74: ESI-MS spectrum of **4** obtained at 298 K, using a Waters SYNAPT G2-Si quadruple time-of-flight (QTOF) HDMS. Inset: structure of **4**.

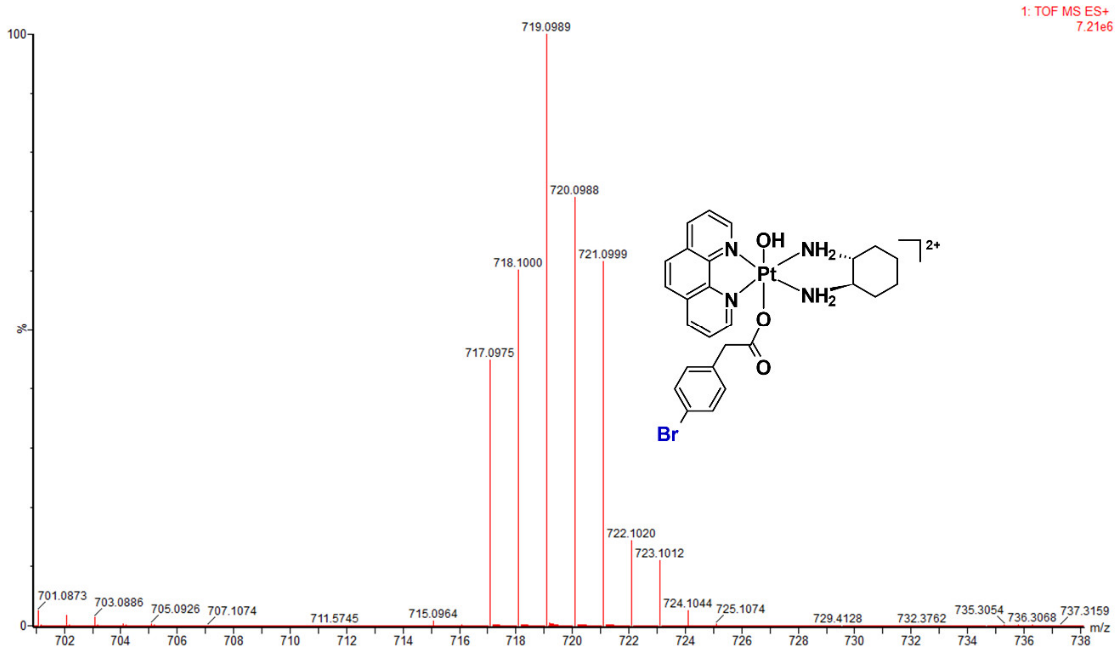

Figure S75: ESI-MS spectrum of **5** obtained at 298 K, using a Waters SYNAPT G2-Si quadruple time-of-flight (QTOF) HDMS. Inset: structure of **5**.

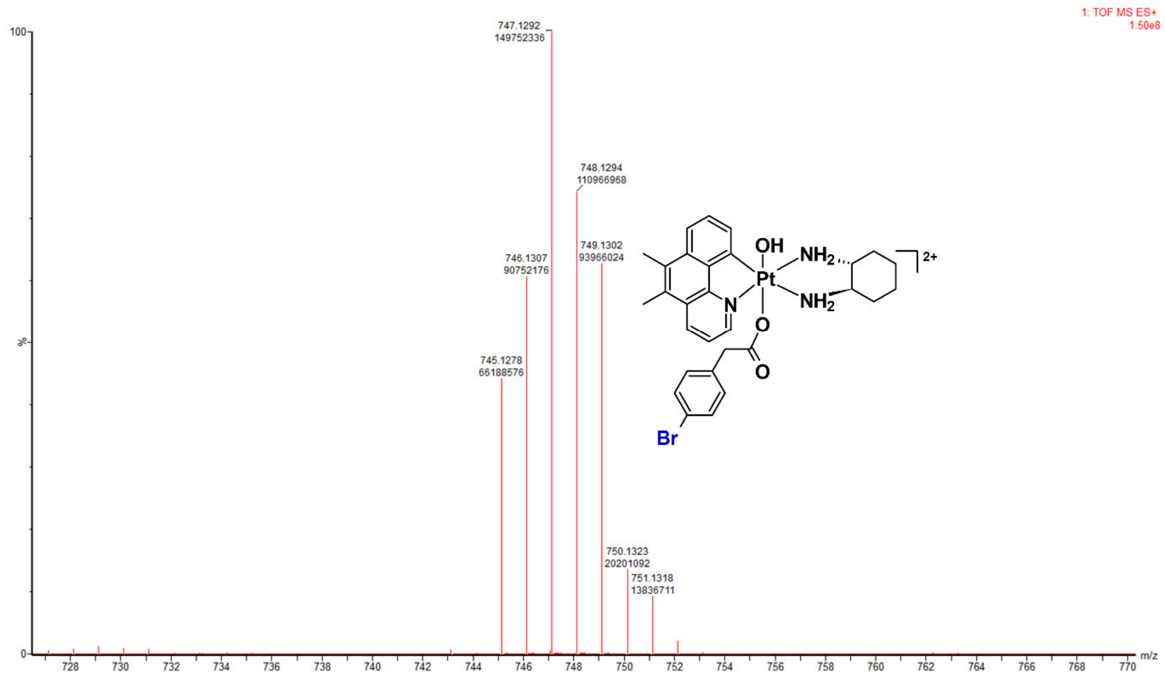

Figure S76: ESI-MS spectrum of **6** obtained at 298 K, using a Waters SYNAPT G2-Si quadruple time-of-flight (QTOF) HDMS. Inset: structure of **6**.

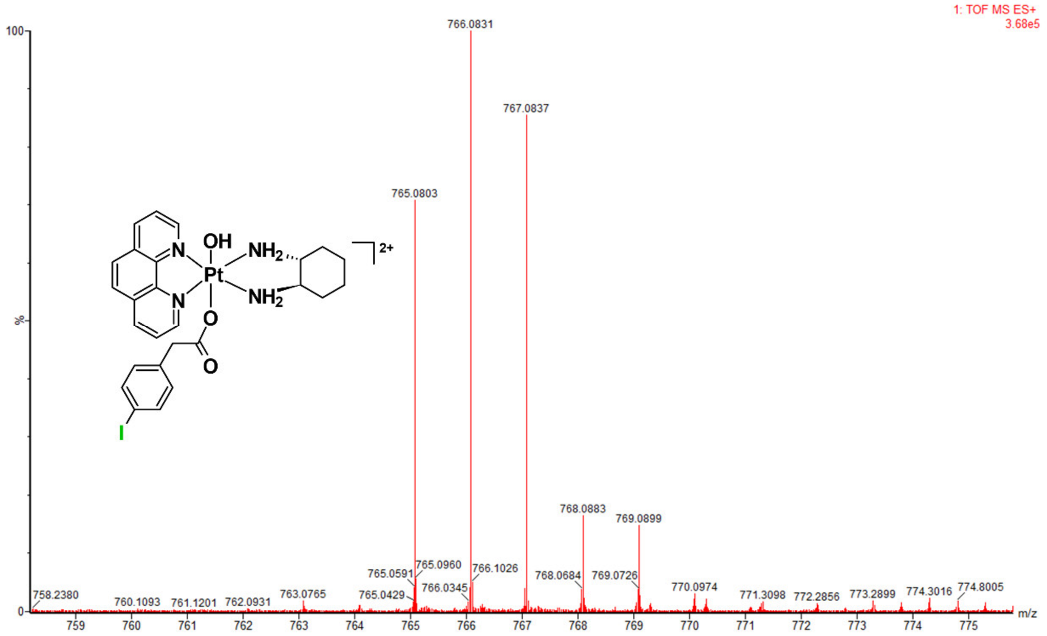

Figure S77: ESI-MS spectrum of **7** obtained at 298 K, using a Waters SYNAPT G2-Si quadruple time-of-flight (QTOF) HDMS. Inset: structure of **7**.

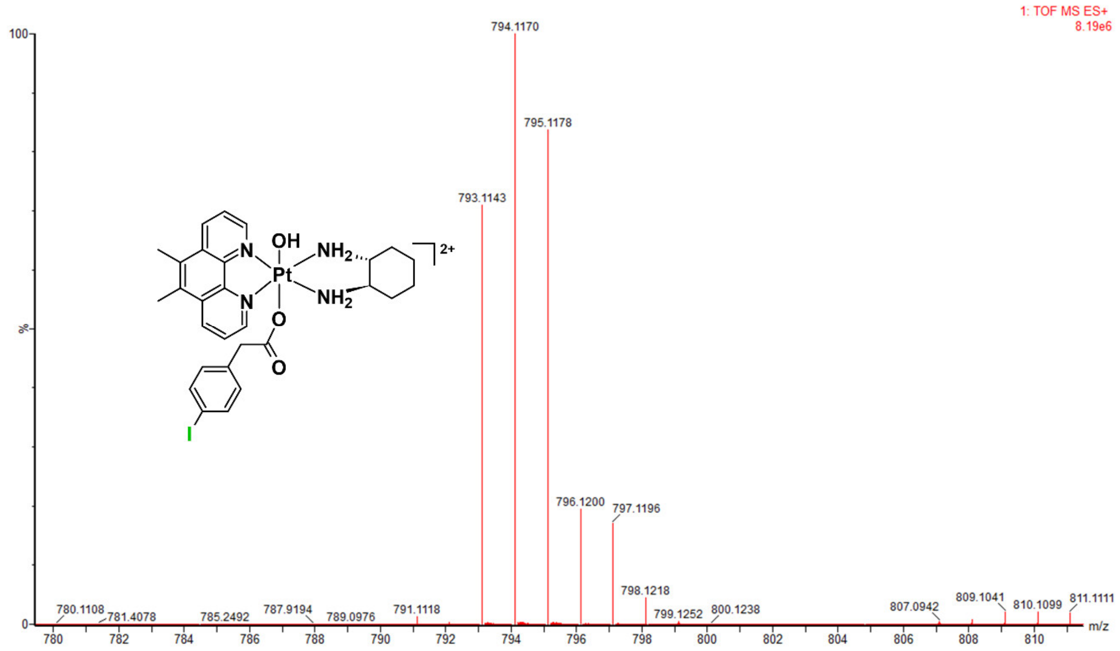

Figure S78: ESI-MS spectrum of **8** obtained at 298 K, using a Waters SYNAPT G2-Si quadruple time-of-flight (QTOF) HDMS. Inset: structure of **8**.

Stability studies

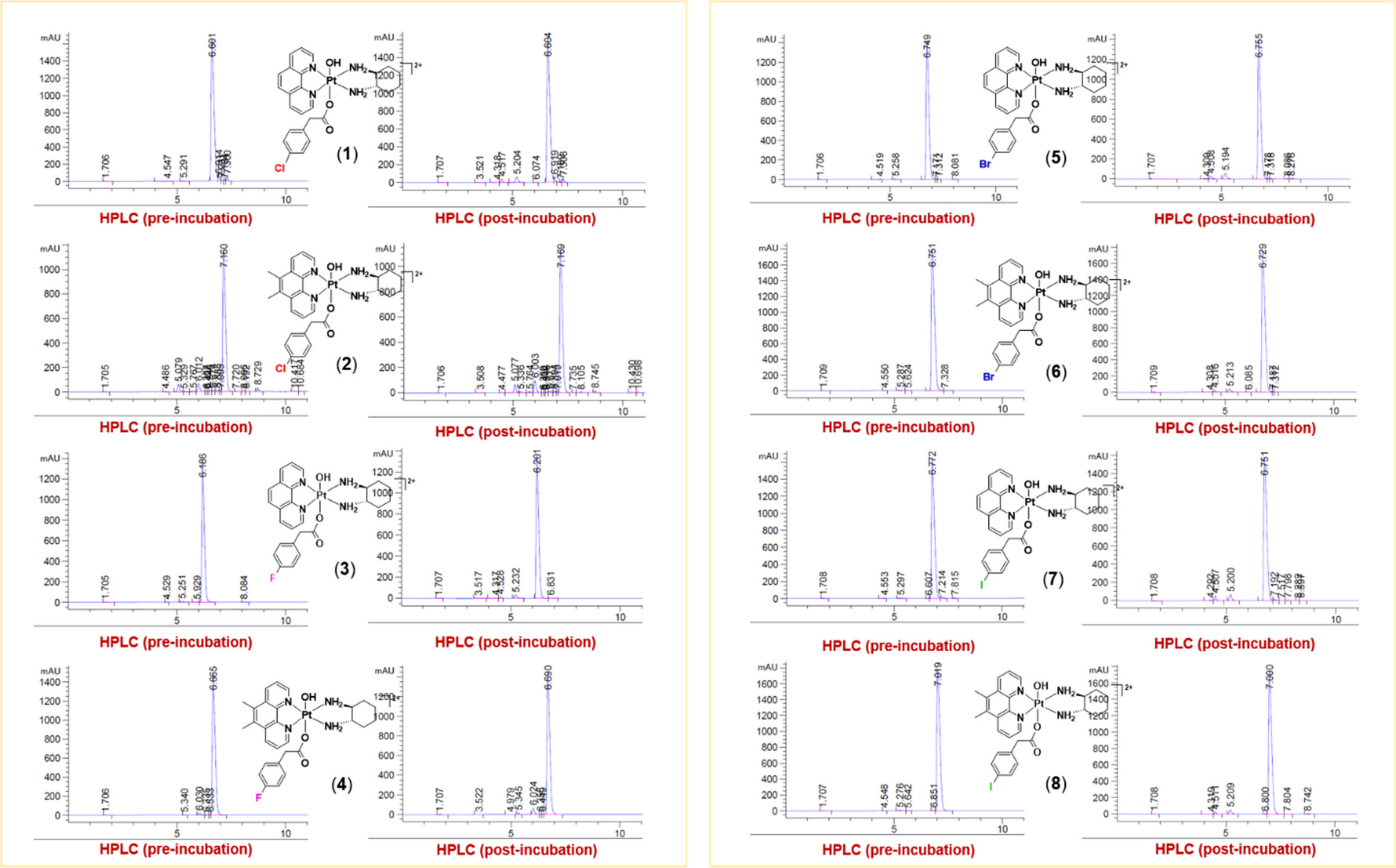

Figure S79: HPLC chromatograms of **1–8** in 10 mM PBS (~7.4 pH) PBS pre- and post-incubation (36 h) obtained at 298 K. Inset: structures of **1–8**.

Lipophilicity measurements

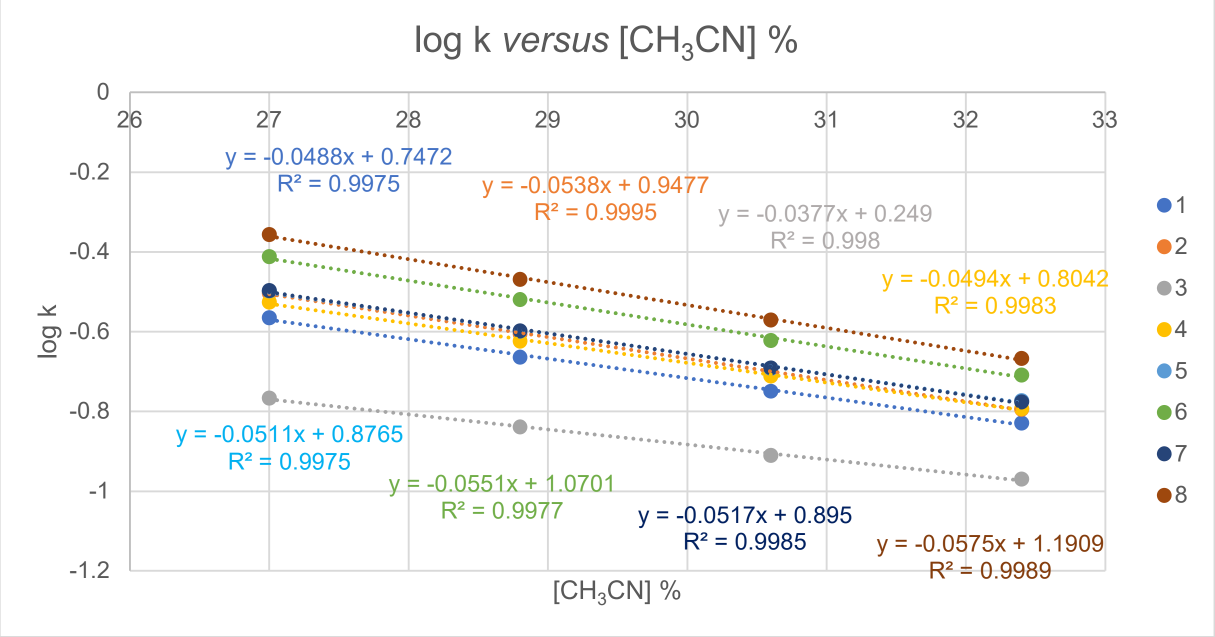

Figure S80: Generated plot curves of log k versus concentration of organic solvent, CH_3CN to determine the chromatographic lipophilicity index, log k_w of **1**–**8**.

Reduction reaction experiments

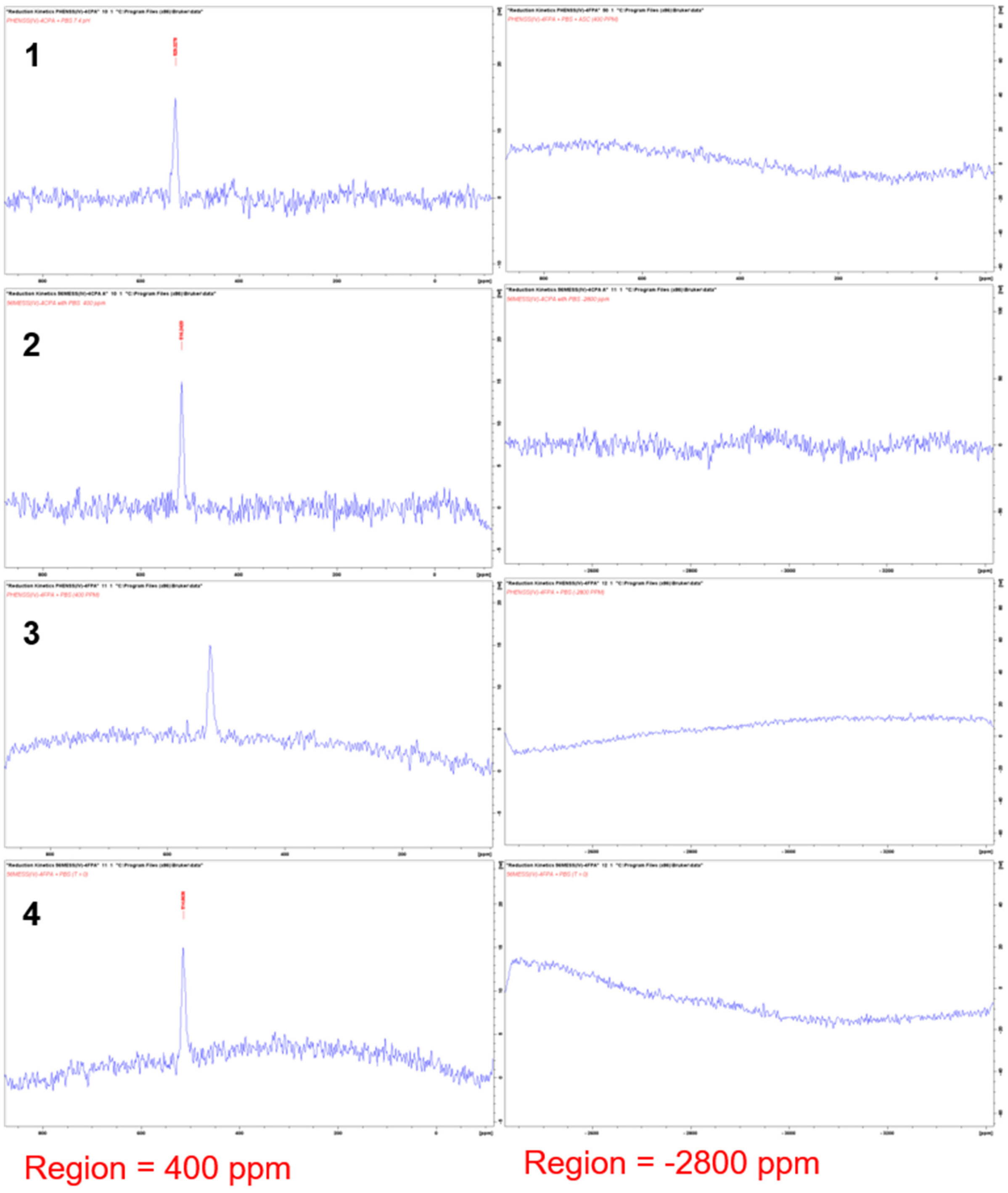

Figure S81: Preliminary 1D-^{195}Pt-NMR spectra of **1**, **2**, **3** and **4** in 10 mM PBS (~7.4 pH) within the regions of -2800 and 400 ppm at 37 ºC.

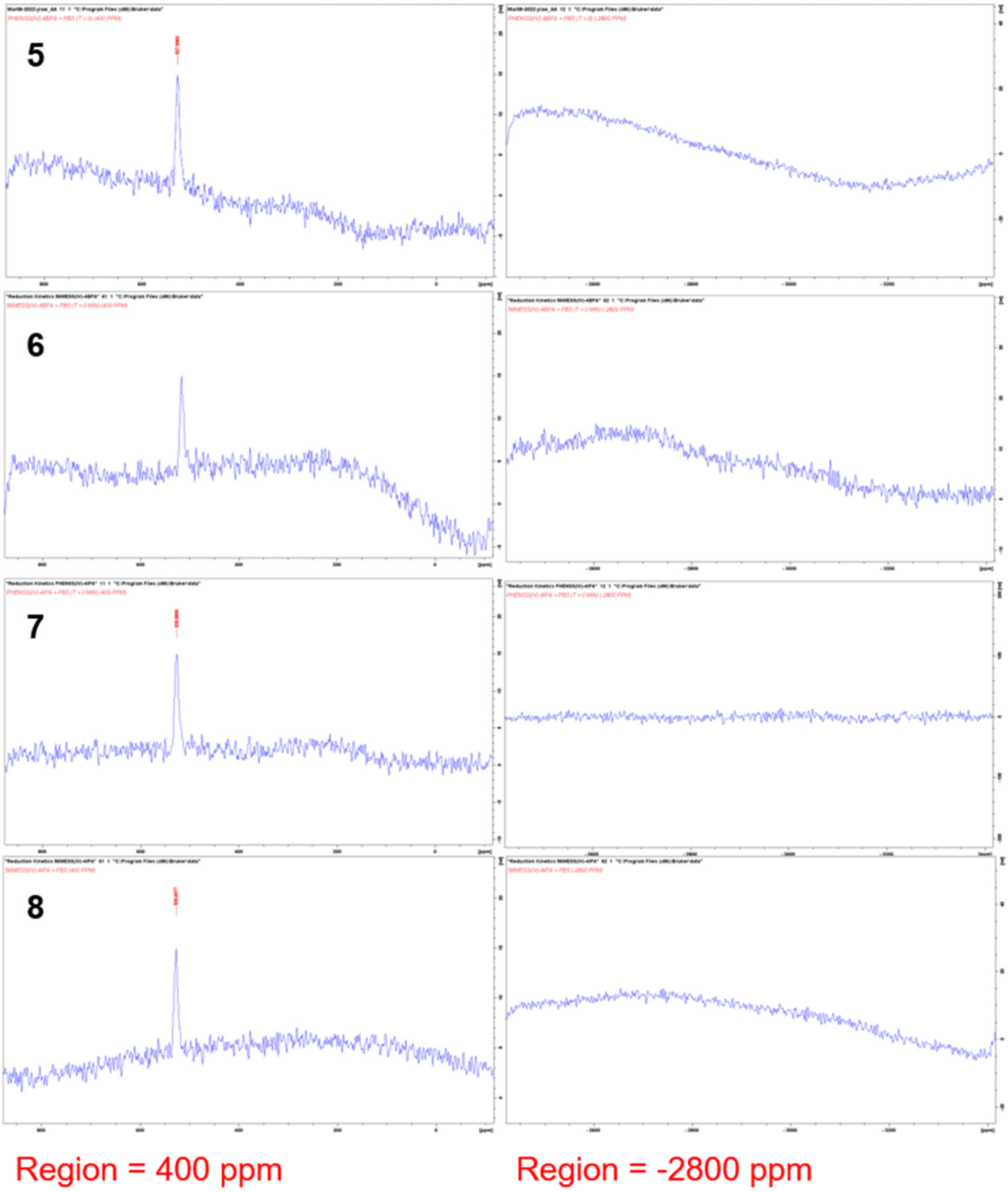

Figure S82: Preliminary 1D-^{195}Pt-NMR spectra of **5**, **6**, **7** and **8** in 10 mM PBS (~7.4 pH) within the regions of -2800 and 400 ppm at 37 ºC.

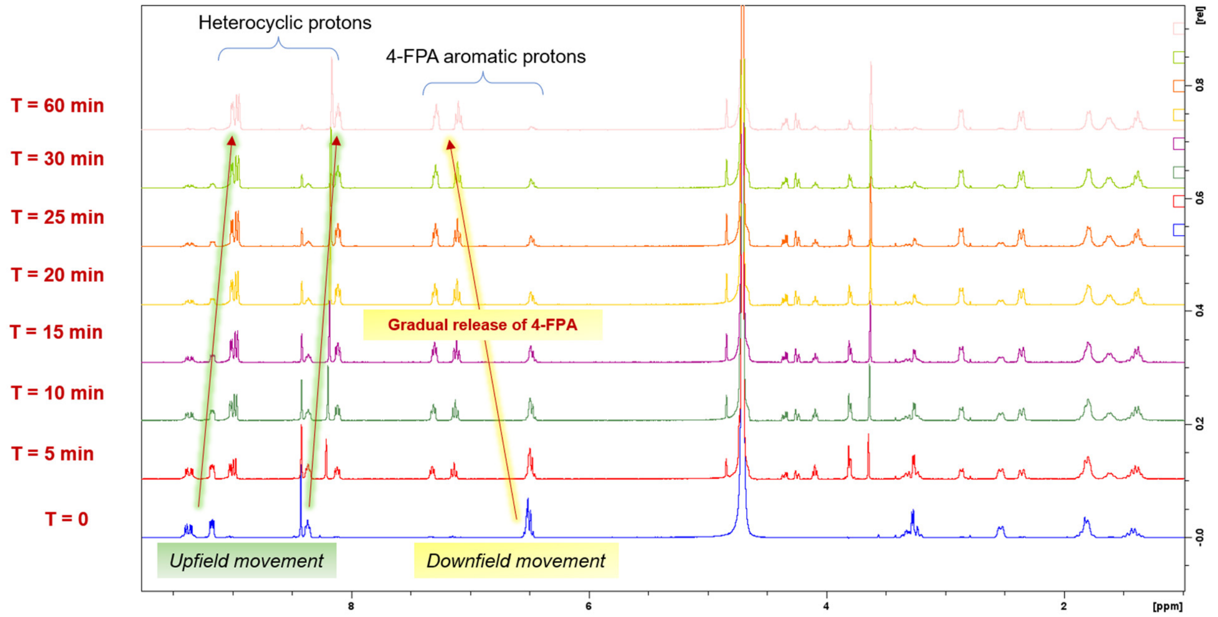

Figure S83: ^{1}H-NMR spectra of **3** with 10 mM PBS (~7.4 pH) and AsA in D_2O at 310.15 K, in different time intervals, highlighting the movement of resonances from the phenanthroline protons and the aromatic protons of the 4-FPA ligand as indicated by the red arrows. **T** represents time in min.

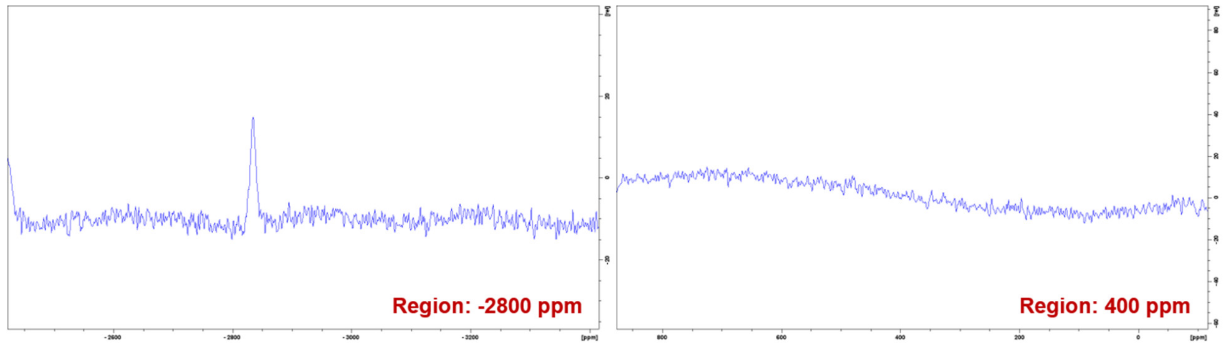

Figure S84: 1D-^{195}Pt-NMR spectra of **3** with 10 mM PBS (~7.4 pH) and AsA in D_2O, within the regions of -2800 and 400 ppm at 310.15 K, highlighting its complete reduction after 1 h from the final ^{1}H-NMR experiment.

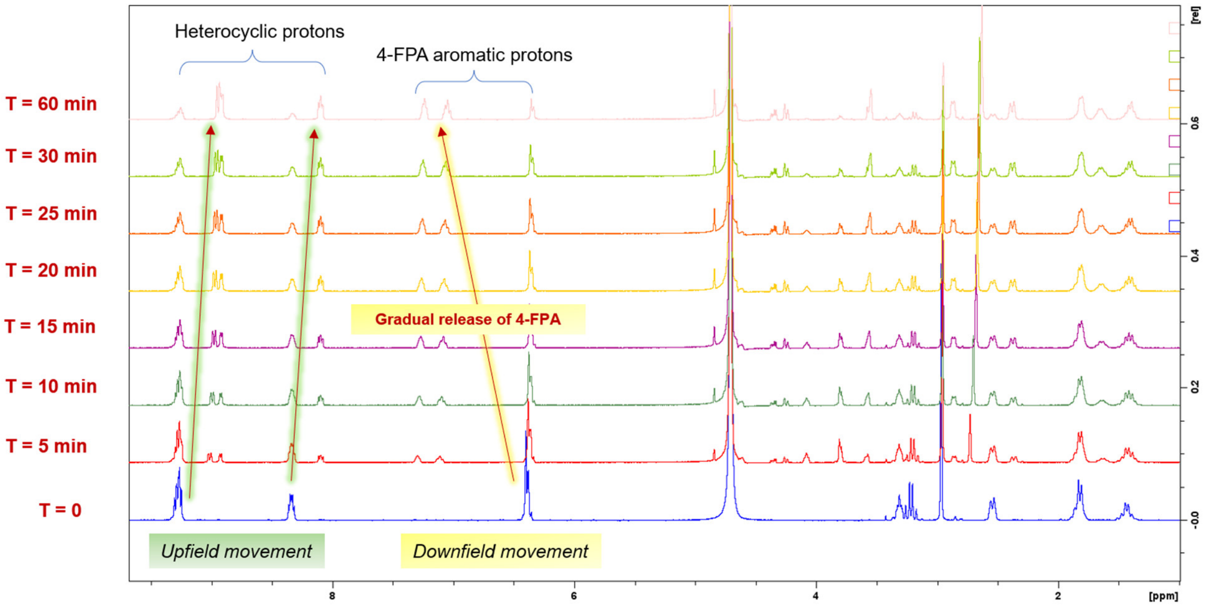

Figure S85: ^{1}H-NMR spectra of **4** with 10 mM PBS (~7.4 pH) and AsA in D_2O at 310.15 K, in different time intervals, highlighting the movement of resonances from the phenanthroline protons and the aromatic protons of the 4-FPA ligand as indicated by the red arrows. **T** represents time in min.

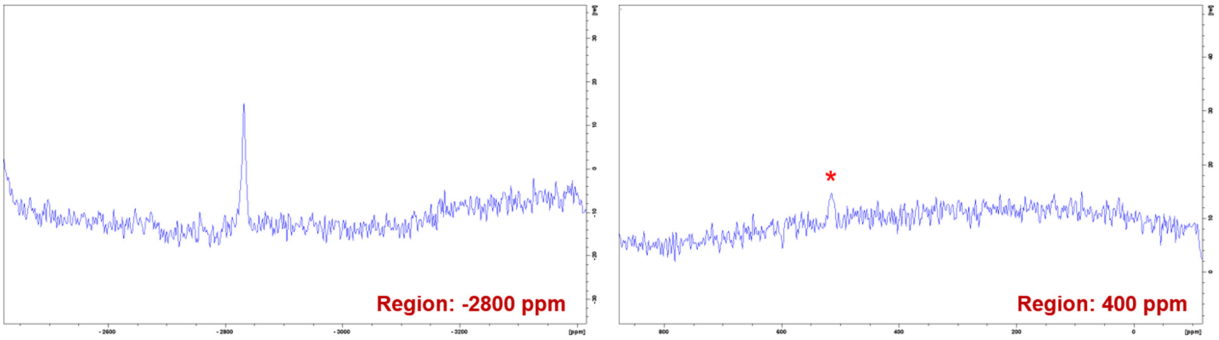

Figure S86: 1D-^{195}Pt-NMR spectra of **4** with 10 mM PBS (~7.4 pH) and AsA in D_2O, within the regions of -2800 and 400 ppm at 310.15 K, highlighting its partial reduction after 1 h from the final ^{1}H-NMR experiment as indicated by *.

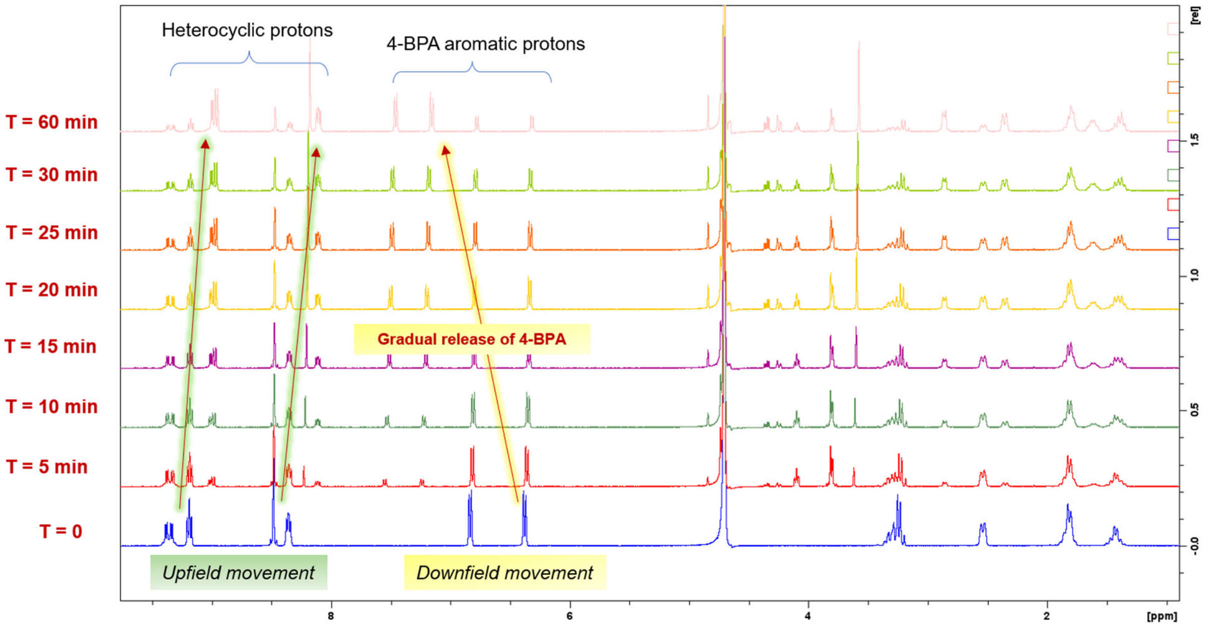

Figure S87: ^{1}H-NMR spectra of **5** with 10 mM PBS (~7.4 pH) and AsA in D_2O at 310.15 K, in different time intervals, highlighting the movement of resonances from the phenanthroline protons and the aromatic protons of the 4-FPA ligand as indicated by the red arrows. **T** represents time in min.

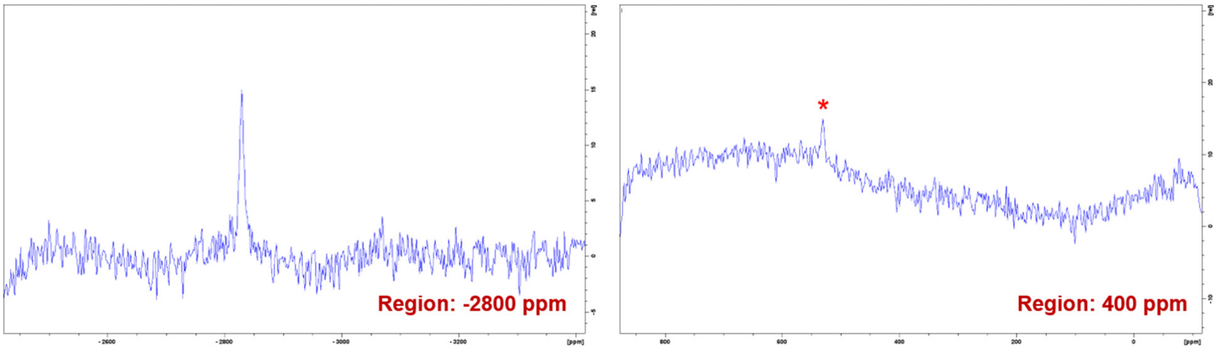

Figure S88: 1D-^{195}Pt-NMR spectra of **5** with 10 mM PBS (~7.4 pH) and AsA in D_2O, within the regions of -2800 and 400 ppm at 310.15 K, highlighting its partial reduction after 1 h from the final ^{1}H-NMR experiment as indicated by *.

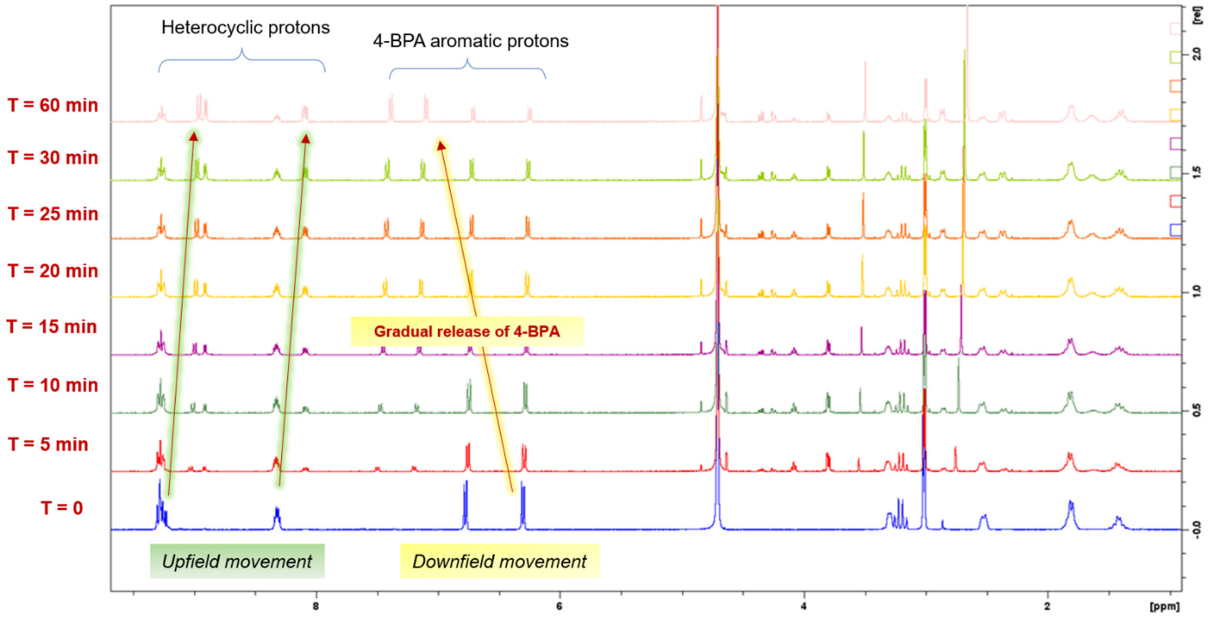

Figure S89: ^{1}H-NMR spectra of **6** with 10 mM PBS (~7.4 pH) and AsA in D_2O at 310.15 K, in different time intervals, highlighting the movement of resonances from the phenanthroline protons and the aromatic protons of the 4-FPA ligand as indicated by the red arrows. **T** represents time in min.

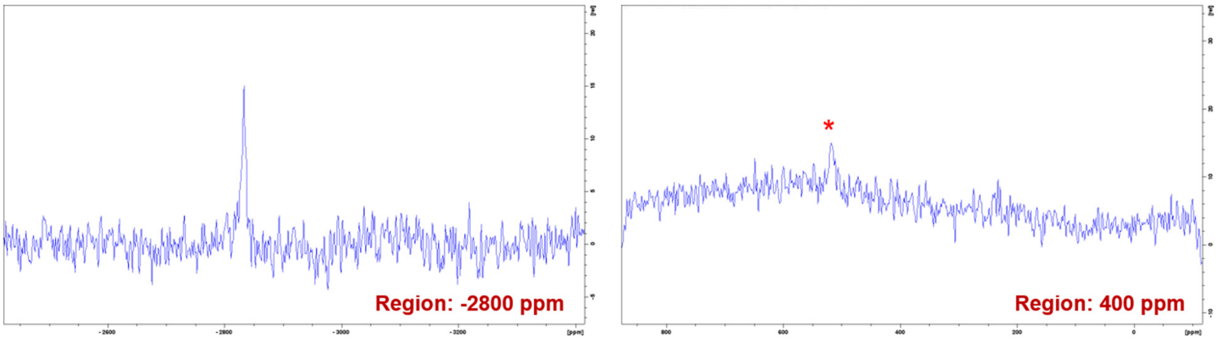

Figure S90: 1D-^{195}Pt-NMR spectra of **6** with 10 mM PBS (~7.4 pH) and AsA in D_2O, within the regions of -2800 and 400 ppm at 310.15 K, highlighting its partial reduction after 1 h from the final ^{1}H-NMR experiment as indicated by *.

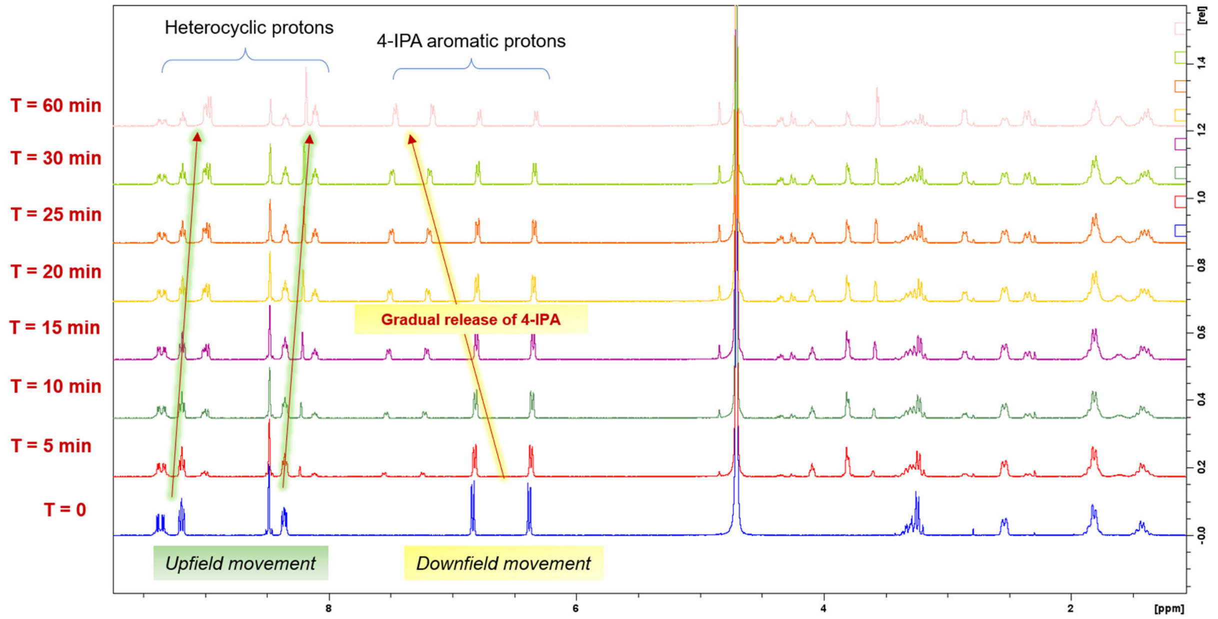

Figure S91: ^{1}H-NMR spectra of **7** with 10 mM PBS (~7.4 pH) and AsA in D_2O at 310.15 K, in different time intervals, highlighting the movement of resonances from the phenanthroline protons and the aromatic protons of the 4-FPA ligand as indicated by the red arrows. **T** represents time in min.

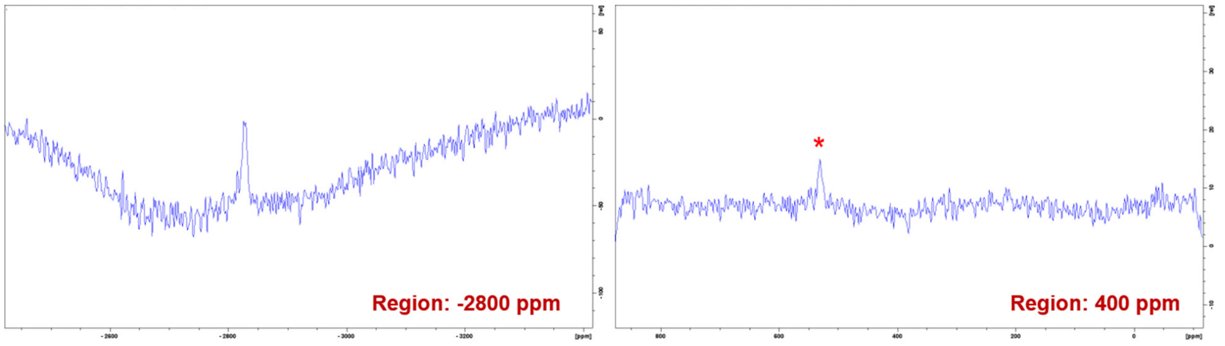

Figure S92: 1D-^{195}Pt-NMR spectra of **7** with 10 mM PBS (~7.4 pH) and AsA in D_2O, within the regions of -2800 and 400 ppm at 310.15 K, highlighting its partial reduction after 1 h from the final ^{1}H-NMR experiment as indicated by *.

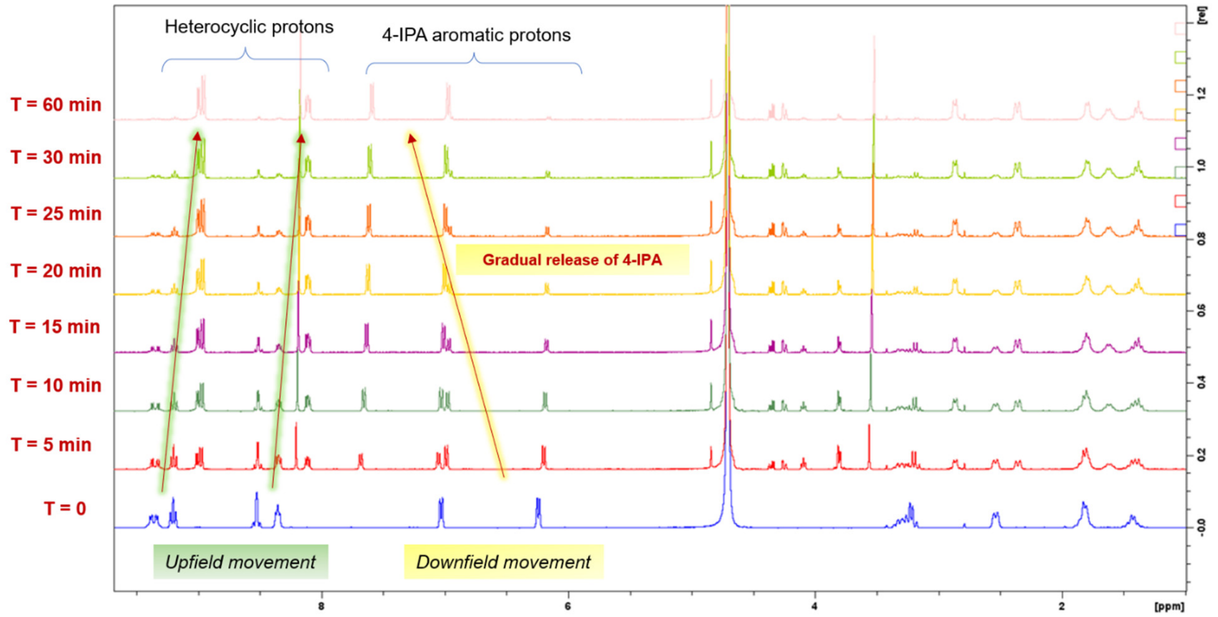

Figure S93: ^{1}H-NMR spectra of **8** with 10 mM PBS (~7.4 pH) and AsA in D_2O at 310.15 K, in different time intervals, highlighting the movement of resonances from the phenanthroline protons and the aromatic protons of the 4-FPA ligand as indicated by the red arrows. **T** represents time in min.

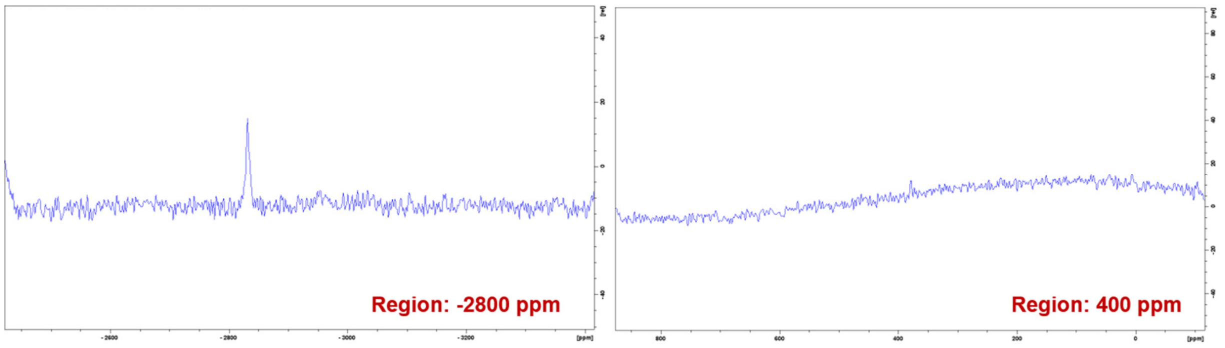

Figure S94: 1D-^{195}Pt-NMR spectra of **8** with 10 mM PBS (~7.4 pH) and AsA in D_2O, within the regions of -2800 and 400 ppm at 310.15 K, highlighting its complete reduction after 1 h from the final ^{1}H-NMR experiment.

ROS experiments

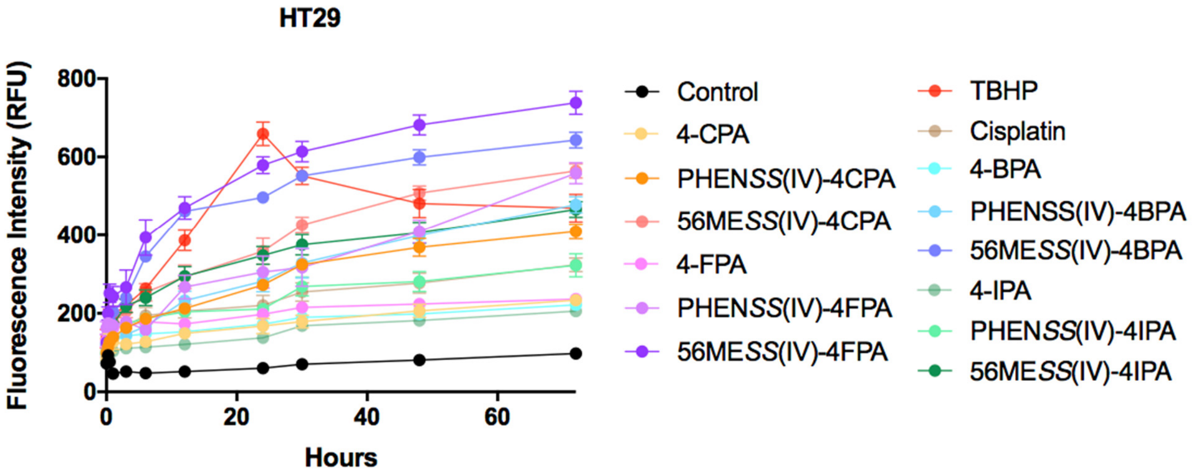

Figure S95: ROS production upon treatment with **1**–**8**, 4-CPA, 4-FPA, 4-BPA, 4-IPA and cisplatin in HT29 colon cells at 0, 0.25, 0.5, 1, 3, 6, 12, 24, 48 and 72 h. **PHEN*SS*(IV)-4CPA** (**1**), **56ME*SS*(IV)-4CPA** (**2**), **PHEN*SS*(IV)-4FPA** (**3**), **56ME*SS*(IV)-4FPA** (**4**), **PHEN*SS*(IV)-4BPA** (**5**), **56ME*SS*(IV)-4BPA** (**6**), **PHEN*SS*(IV)-4IPA** (**7**), **56ME*SS*(IV)-4IPA** (**8**). TBHP: t-butyl hydroperoxide. Data points denote mean ± SEM. *n* = 3 from three independent experiments where samples were run in triplicates.
